# Pan-Ebolavirus nanoparticle vaccine provides protection in rodents from lethal infection by Zaire and Sudan viruses

**DOI:** 10.1038/s41467-026-76114-1

**Published:** 2026-08-08

**Authors:** Connor Weidle, Natalie Brunette, Samuel P. Wrenn, Brooke Fiala, Rashmi Ravichandran, Kenneth D. Carr, Samantha E. Zak, Elizabeth E. Zumbrun, Russell R. Bakken, Michael Murphy, Zhitong Peng, Sidney Chan, Rebecca Skotheim, Lauren Carter, Colin E. Correnti, John M. Dye, David Baker, Neil P. King, Andrew J. Borst, Lance J. Stewart

**Affiliations:** 1 Department of Biochemistry, University of Washington, Seattle, USA; 2 Institute for Protein Design, University of Washington, Seattle, USA; 3 United States Army Medical Research Institute of Infectious Diseases (USAMRIID), Fort Detrick, USA; 4 Clinical Research Division, Fred Hutch Cancer Center, Seattle, USA; 5 Howard Hughes Medical Institute, University of Washington, Seattle, USA; 6 Present Address: Archon Biosciences, Seattle, USA; 7 Present Address: Bonum Therapeutics, Seattle, USA; 8 Present Address: Gates Medical Research Institute, Cambridge, USA

**Keywords:** Cryoelectron microscopy, Protein vaccines, Viral infection, Viral proteins

## Abstract

Both Zaire *ebolavirus* (EBOV) and Sudan *ebolavirus* (SUDV) are members of the genus Ebolavirus and cause outbreaks marked by high fatality rates and repeated spillover from animal reservoirs. Filoviral glycoproteins (GPs) are the primary targets of neutralizing antibodies and form the basis of current vaccines. Here we describe the design, structural characterization, and evaluation of two-component self-assembling icosahedral I53-50 nanoparticles displaying prefusion EBOV or SUDV GP antigens, either individually or in cocktail and mosaic multivalent formats. EBOV-GP-I53-50 and SUDV-GP-I53-50 nanoparticles elicited strong homologous protection in mice and guinea pigs. In the mouse-adapted EBOV model (maEBOV), mosaic and cocktail formulations produced weak survival below that of the matched EBOV-GP-I53-50 GP immunogen. In contrast, in the gpaSUDV guinea pig model (gpSUDV), cocktail and mosaic nanoparticles elicited robust protection against gpSUDV, and detectable antibody responses to both SUDV and EBOV GPs. These findings demonstrate that multivalent GP-I53-50 nanoparticle immunogens can protect rodents from death, severe clinical signs of disease, and weight loss in relevant models. Together with the established clinical safety of the I53-50 platform, these results support continued efforts toward the development of a pan-ebolavirus vaccine.

## Abstract (web summary)

This study describes the design and structural characterization of two-component self-assembling nanoparticles co-displaying prefusion Zaire ebolavirus (EBOV) and Sudan ebolavirus (SUDV) glycoprotein antigens with protective capacity.

## Introduction

The *Ebolavirus* genus is a member of the single-stranded negative-sense RNA virus family *Filoviridae* and includes both *Zaire ebolavirus* (EBOV) and *Sudan ebolavirus* (SUDV). The study of filoviruses and the development of vaccines against this family have been difficult, in part due to the high biosafety level required for research. Ebolaviruses were first discovered after 1976 outbreaks in southern Sudan (now South Sudan) and Zaire (now the Democratic Republic of Congo) and have remained a major public health issue. The most common Ebola virus disease (EVD^1^) outbreaks are caused by EBOV, with over 20 outbreaks in Sub-Saharan Africa having case-fatality rates ranging from 40%–70%^2,3^. The largest ever outbreak of EVD occurred in West Africa from 2013–2016, where EBOV resulted in over 28,000 cases and over 11,000 deaths. Additionally, in Uganda from 2000–2001, SUDV caused 425 cases of Sudan virus disease (SVD), with 224 reported deaths–the largest known SUDV outbreak to date^4^. In 2018–2020 EBOV re-emerged causing 3481 cases with 2299 deaths^5^. 2022 saw SUDV also re-emerge, causing more than 160 confirmed or probable cases^6^ and with 55 confirmed deaths^7^. SUDV re-emerged yet again in 2025 with 4 deaths (2 confirmed and 2 probable) and 12 confirmed and 2 probable cases^8^. Considering the life-threatening, repeating, and disruptive nature of EVD and SVD outbreaks, there is a tremendous need for effective vaccines that provide broad-spectrum prophylactic protection against both EBOV and SUDV.

Several species-specific EBOV^9,10^ and SUDV^11,12^ vaccine candidates have been shown to protect against lethal infection in non-human primates (NHPs). Currently, the two licensed Ebola vaccines are approved only for prevention of EVD caused by EBOV^13^. Merck’s Ervebo® was the first and remains the only EVD vaccine to be approved by the Food and Drug Administration (FDA) in the United States (November 2019). It was developed by the Public Health Agency of Canada and later licensed to Merck for further development and manufacturing. The vaccine, also known as rVSV-ZEBOV, is a replication-competent, live-attenuated recombinant vesicular stomatitis virus (VSV) vaccine that expresses the main trimeric GP from *Zaire ebolavirus*^10,14^. In 2015, over two thousand close contacts of confirmed Ebola patients were ring-vaccinated with rVSV-ZEBOV on an emergency basis during the outbreak in West Africa to reduce the spread of the infection, with an observed vaccine efficacy of 100%^15^. Following a large-scale ring vaccination effort in 2019 with 91,492 vaccinated individuals, the World Health Organization (WHO) published preliminary results stating that the rVSV-ZEBOV vaccine was 97.5% effective at preventing transmission of the virus^16^. A final analysis showed 10 days post vaccination that a low EVD onset rate of 0.16 per 1000 (in 32 of 194,019 participants) was observed. Additionally, mortality among vaccinated individuals who developed EVD decreased with increasing time between vaccination and symptom onset: vaccinees already experiencing EVD (23% mortality; 106 deaths of 462 cases), onset 0–9 days (26% mortality; 99 deaths of 385 cases), onset 10–29 days (14% mortality; 5 deaths & 35 cases), onset +30 days (5% mortality; 2 deaths & 42 cases), as compared to a total unvaccinated outbreak mortality rate of 66% (2287 deaths of 3470 cases)^17^. For SUDV, ring vaccinations trials were performed to combat the 2025 SUDV outbreak, using SUDV adapted rVSV^18,19^, which may have shortened the severity and length of the recent outbreak, though the direct impact of this campaign has yet to be officially reported.

The current success of VSV ring vaccinations highlights a promising approach for effectively combating ongoing *ebolavirus* pandemics. However, this approach requires a species-specific response. Numerous studies have shown that obtaining cross-protection against both EBOV and SUDV with a single GP based vaccine is challenging. In humans infected with either EBOV or SUDV, or vaccinated against EBOV with 1 or 2 doses of the rVSV-ZEBOV vaccine, antibody cross-reactivity between stabilized EBOV and SUDV GP trimers^20^ occurs only occasionally and typically with markedly reduced binding and neutralization titers^21^. Additionally, a phase 1 clinical trial using only ZEBOV GP encoded by the viral vector chimpanzee adenovirus (ChAd3) generated no detectable SUDV GP specific antibodies at 28 days post dose, despite generating strong levels of EBOV neutralizing antibodies^22^. Anti-SUDV GP antibodies could only be observed in individuals following a boost with a modified Vaccinia Ankara (MVA) vector (MVA-BN Filo - Mvabea^13^), which includes the same ZEBOV GP as ChAd3, plus the GPs of SUDV and Marburg virus (MARV), and the nucleoprotein of Taï Forest Ebola virus (TAFV). In repurposed non-human primates (NHPs) from a successful rVSV-EBOV vaccine efficacy study following EBOV challenge, there was little cross-protection to SUDV challenge, even though animals were found to develop some cross-reactive humoral responses to SUDV^23,24^. The lack of heterologous cross-viral EVD protection from GP based vaccines is likely due to the significant amino acid sequence differences between EBOV and SUDV GPs, which have 55% amino acid sequence identity over their full open reading frames (Supplementary Fig. 1).

Ring vaccinations offer a much less protective response as a post-exposure prophylaxis if patients have already been exposed to *ebolavirus;* an unfortunate feature of these campaigns as they combat an epidemic in real-time. For these reasons, a multivalent vaccine that could generate a broad and protective *ebolavirus* response is of great interest as a preventative measure for at-risk communities. One such multivalent vaccine regimen is the two-dose Zabdeno/Mvabea (Ad26.ZEBOV/MVA-BN-Filo); approved by European Medicines Agency (EMA) for prevention of EVD caused by *Zaire ebolavirus*. Zabdeno/Mvabea uses a prime with Ad26.ZEBOV (Zabdeno; human adenovirus serotype 26 encoding Zaire Mayinga EBOV-GP) followed 8 weeks later by a heterologous boost with MVA-BN-Filo (Mvabea)^13^. Although the vaccine regimen employs a heterologous boost, the extended 8-week interval between doses reduces its practicality during outbreaks. For these reasons, the world health organization (WHO) advised not using this vaccine to combat the 2022 SUDV outbreak^25^. Furthermore, a 2025 human clinical trial looked at different Ad26.ZEBOV MVA-BN-Filo boost strategies to see if broad protection against *ebolavirus* was possible with this vaccine^26^. Plasma collected post immunization showed vaccine-induced humoral responses that almost exclusively targeted EBOV GP with infrequent antibody recognition of SUDV GP or *Reston ebolavirus* (RESTV) GP. Another bivalent vaccine with EBOV and SUDV GP (ChAdOx1 biEBOV)–a non-replicating single-adenoviral vaccine–was considered to combat the 2022 SUDV outbreak. However, it was too slow to be implemented, as the vaccine shipped after the outbreak had been resolved^26^. A later study showed the vaccine did not protect NHPs from SUDV challenge^27^. Additionally, a phase 1 clinical trial revealed that while anti-SUDV GP antibodies were elicited in medium-dose and high-dose groups, no SUDV neutralizing antibodies were detected in a SUDV pseudo-neutralization assay^28^. Thus, an approved multivalent vaccine that can protect broadly against *ebolavirus* species is still needed.

Although neither of the two licensed EBOV vaccines nor the leading clinical candidates provide broad EVD protection, several preclinical vaccine platforms have demonstrated cross-ebolavirus and even broader filovirus protection in NHP studies. A bivalent single complex adenovirus-based viral vector (CAdVax) that expresses EBOV and SUDV GP “EBO7”, provided protection to NHPs with a single dose, with 3/3 and 2/3 NHPs surviving an aerosol challenge with EBOV or SUDV respectively without any signs of clinical disease^29^. Furthermore, a third group of NHPs that received a prime boost immunization with EBO7 saw a 100% survival rate with 3/3 NHPs surviving an aerosol challenge with SUDV^29^. In another study, three separate VSV vaccines expressing EBOV, SUDV and MARV GPs were blended together^30^. With a single dosage of the VSV cocktail, 9 NHPs total in 3 groups of 3 NHPs survived challenges with EBOV SUDV or MARV^30^. These two examples demonstrate the strong pre-clinical potential for achieving broad EVD protection using either a bivalent or blended vector-based vaccine. This result has also been demonstrated with a protein subunit-based vaccine using full length GPs of EBOV, MARV, SUDV purified from insect cells, which generated a strong protective heterologous *Filoviridae* response in NHPs with three doses with CoVaccine HT™ adjuvant. NHPs vaccinated with a combination of EBOV and MARV GPs had full protection from MARV challenge, NHPs vaccinated with EBOV and SUDV survived a SUDV challenge, and NHPs vaccinated with just EBOV were protected from an EBOV challenge^31^. A follow-up NHP prime-boost study using co-lyophilized SUDV plus MARV GPs afforded protection against both SUDV and MARV challenges^32^.

In addition to these vaccine platforms, protein-based nanoparticle (NP) and virus-like particle (VLP) systems have gained traction in modern vaccine design, as the repetitive antigen array on these particles enhances antigen-presenting cell uptake and drives stronger B-cell activation through avidity effects^33,34^. As an example, SKYCovione™, a self-assembling NP vaccine, was invented in 2020^35–37^ during the COVID-19 pandemic, utilizing the computationally designed I53-50 nanoparticle^38^. I53-50 is constructed from 20 trimeric antigen-displaying “I53-50A” and 12 pentameric “I53-50B” components that can be separately produced and subsequently mixed in solution to cooperatively self-assemble into a uniform 120-subunit icosahedral nanoparticle (NP)^39^. Other promising examples of nanoparticle-based Ebola GP vaccines include: VLPs co-expressed with VP40^40^, ferritin NPs^41^, hepatitis C virus E2 core (E2p) based NPs^42–44^, or the computationally designed I3-01 NP^42,45^.

Since multivalent NP immunogens can induce heterotypic prophylaxis^46–49^, they offer the potential to achieve broad-spectrum protection from vaccination. Because the antigen-displaying I53-50A nanoparticle component exhibits cyclic C3 symmetry, it is well suited for genetic fusion to class I trimeric viral fusion glycoproteins. This is typically achieved by connecting the C terminus of the glycoprotein to the N terminus of I53-50A through a flexible peptide linker^47,48,50^. The improved solution behavior of trimeric glycoprotein fusions to the I53-50A component affords a facile manufacturing paradigm where ratios of different immunogens can in principle be rapidly formulated with the I53-50B pentameric subunit into a single mosaic NP vaccine product for broad-spectrum vaccine formulation. Here, we apply the I53-50 NP platform to generate mosaic and cocktail nanoparticles for co-displaying EBOV and SUDV GPs and assess whether multivalent display can drive cross-reactive anti-GP antibody responses and protection in two rodent models.

## Results

### Prefusion EBOV glycoprotein immunogen design, production and characterization

**Fig. 1:**
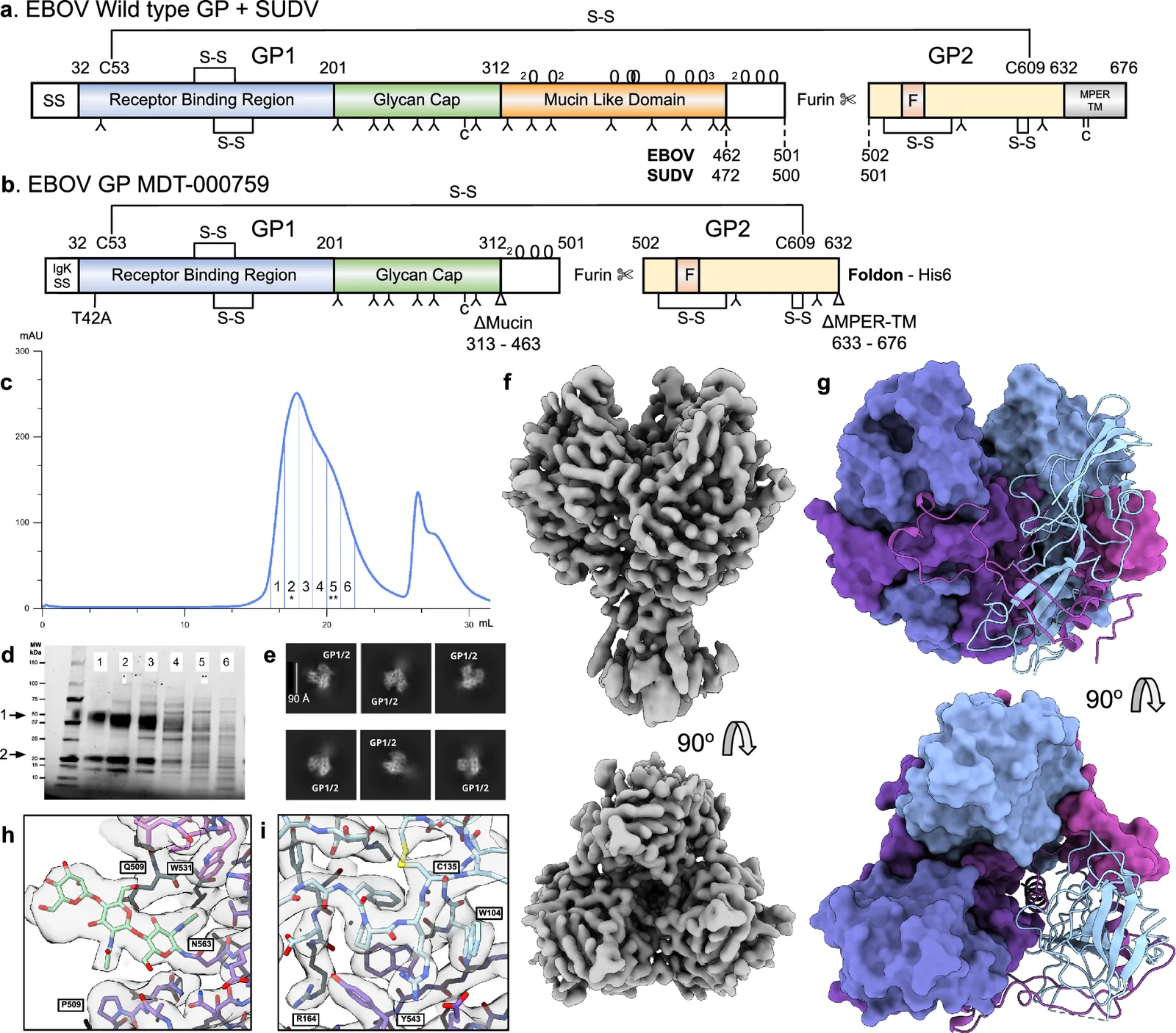
Domain architecture of mature ebolavirus GP and cryoEM structural characterization of the EBOV-GP-foldon construct. **a** The wild-type domain protein architecture for mature EBOV and SUDV GPs are illustrated with residue numbers and main domain boundaries noted. Glycosylation only shown for EBOV^55^ **b** Using the same nomenclature as in (**a**) the EBOV-GP-Foldon construct (MDT-000759) domain architecture is illustrated with its deletion of the mucin-like (∆Muc) and the MPER-TM (∆MPER-TM) domains with C-terminal genetic fusion to the foldon domain followed by six histidine residues (His6) to facilitate IMAC purification. This construct also has the T42A mutation to knock out the first N-linked glycosylation similar to (PDB ID: 5JQ3) for EBOV^63^. Nomenclature for (**a**, **b**) is as follows; secretion signal peptide (SS) or IgK SS which is removed during translocation through the endoplasmic reticulum, the receptor binding region (blue), the glycan cap (green), and the mucin-like domain (orange) in GP1 which is separated from GP2 (yellow) by a furin cleavage site with fusion (F) domain (pink), membrane proximal external region (MPER) followed by the transmembrane (TM) (gray) and covalent modifications of disulfide bonds (S-S), N-linked (upside down Y), O-linked (O) (2,3 notation denote multiple neighboring O glycan sites), or C-linked glycosylation (**c**)^55,114^. **c** EBOV-GP-Foldon was produced as a secreted protein from the pCMV/R vector by transient transfection of ExpiHEK293F cells and purified by IMAC and then SEC (see “Methods”) **d** SDS polyacrylamide gel electrophoresis (SDS-PAGE), where fractions 1 and 2 were combined and used for cryoEM studies; sample is boiled and reduced^115^. Band 1 is GP1-∆Mucin-like domain. Band 2 is GP2-(∆MPER-TM)-Foldon. Molecular weight marker in kilodaltons, MW kDa, left. SEC Purification and SDS-PAGE Analysis were performed twice. **e** CryoEM 2D class averages for EBOV-GP-Foldon. **f** The 3.05 Å cryoEM map, non-uniform refinement map CryoSPARC, DeepEMhancer sharpened. **g** Refined cryoEM structure GP2 (3 pink/purple chains) GP1 (3 blue/purple chains). **h** Built structure fit to density on the external region only, showing a resolved glycan at N563. **i** Built structure fit to density on the internal region (Supplementary Fig. 4).

Informed by previous studies using prefusion viral glycoprotein trimers fused to the trimeric component of the I53-50 nanoparticle platform, we employed a stepwise approach to the design and characterization of EBOV and SUDV GP immunogens. We first considered the design of the EBOV and SUDV GP antigens on their own, prior to fusing them to the I53-50A trimer component of the I53-50 NP. In this endeavor, we considered the GP sub-domains that should or should not be incorporated into the final GP antigenic constructs. Notably, the post-Golgi full-length Ebolaviral GP ( ~ 160 kDa) contains both N- and O-linked carbohydrate modifications which are further processed by furin proteolysis at the plasma membrane^51^ before finally presenting on the surface of enveloped virions as trimers of heterodimers, GP1 ( ~ 140 kDa) and GP2 ( ~ 27 kDa) (Fig. 1a). The GP1 and GP2 units are linked together through a disulfide bond formed between cysteine residues C53 on GP1, which contains the receptor binding region (RBR), and C609 on GP2^52^, which contains the protein machinery responsible for the fusion of the viral and host cell membranes^53^.

Since the heavily glycosylated mucin-like domain (muc) is thought to act as a “glycan shield” or decoy epitope for non-neutralizing antibodies^54,55^, and may even serve as an epitope for antibody-dependent enhancement of infection^56^, we first sought to design “∆muc” deleted versions of the GPs that lacked the mucin-like domain. Furthermore, an antibody response against the mucin domain may be non-neutralizing for viral infection since the mucin domain is proteolytically removed from the virion surface during the infection process^57,58^. In addition, to ensure secretion of the constructs from mammalian cells, we removed the membrane proximal external region (MPER) and transmembrane (TM) helix^59^ from the C terminus of GP2 (∆MPER-TM). Finally, rather than stabilize prefusion GPs with designed point mutations^20^ or using the inherently less stable Mano River 2014 strain sequence^60^ (which is known to compromise prefusion GP integrity^61^) (Supplementary Fig. 2), we sought to preserve as much as possible the native ancestral (Zaire Mayinga or Sudan Gulu) sequences of our ∆muc, ∆MPER-TM GP constructs. We stabilized them in the trimeric, prefusion conformation through C-terminal fusion to the “foldon” domain of T4 fibritin, which is a commonly used strategy for stabilization of class-I viral fusion proteins^62,63^. Finally, guided by available X-ray crystallographic studies^63–65^, we included a T42A mutation to eliminate an N-linked glycosylation site at Asn40^55^, which has been shown to increase sample homogeneity^63,64^. This construct is hereafter referred to as both EBOV-GP-Foldon and as MDT-000759 (Fig. 1b).

We ultimately explored two different expression systems for the EBOV-GP-Foldon (MDT-000759) construct: firstly through small-scale production in HEK293 cells by lentiviral transduction^66^ (Supplementary Fig. 3), and secondly by transient transfection of ExpiHEK293F cells with pCMV/R vectors (Fig. 1c, d). CryoEM structural analysis on the  purified protein produced by transient transfection revealed that the EBOV-GP-Foldon construct maintained its intended homo-trimeric architecture (Fig. 1e–g). The structure of EBOV-GP-Foldon construct was determined at 3.05 Å globally with an estimated local resolution range spanning 2.79–3.24 Å, and determined at the physiological of pH 7.5 (PDB ID: 9MHA, EMDB ID: EMD-48271, Supplementary Figs. 4 and 5, and Table S1). To our knowledge, this structure provides the first high-resolution cryoEM structure (<3 Å, locally) of an apo EBOV-GP-Foldon construct, providing a near-native view of its conformation free from potential crystal packing effects (Supplementary Fig. 4–6). Indeed, when compared to prior apo EBOV GP structures solved by X-ray crystallography at pH 5.0–5.2^20,42,63^, our apo-state cryoEM structure reveals deviations within and adjacent to the hydrophobic pocket between GP1 and GP2, where several small-molecule anti-viral lead candidates are known to bind^63,65,67–69^ (Supplementary Fig. 7).

Throughout the cryoEM map, the protein backbone and many amino acid side chains are well defined, except for the glycan cap (residues 224–310), which was unresolved despite being present in the construct sequence (Fig. 1h, i). Additionally, as expected^70^, the GP2 HR2-stalk is only somewhat resolved (residues 598–632) and exhibits a large amount of flexibility, and thus a model was not built for this region (Fig. 1f). A single, highly conserved glycan is resolved at position N563, while other conserved glycan sites are located on flexible peptides or domains that did not resolve, including the glycan at N618^71^ (Fig. 1h). Finally, our EBOV-GP-Foldon (MDT-000759) structure aligns closely with a high-resolution crystal structure of the same amino acid sequence (PDB ID: 5JQ3; all-atom RMSD = 0.918 Å), albeit with differences in an anti-viral binding pocket, as noted above. Together, these data indicate that the EBOV-GP-Foldon (MDT-000759) construct produced in this study adopts a well-defined and homogeneous prefusion trimer, providing confidence in its suitability for subsequent nanoparticle display and downstream immunogenicity studies.

### GP-I53-50A design, production, and characterization

**Fig. 2:**
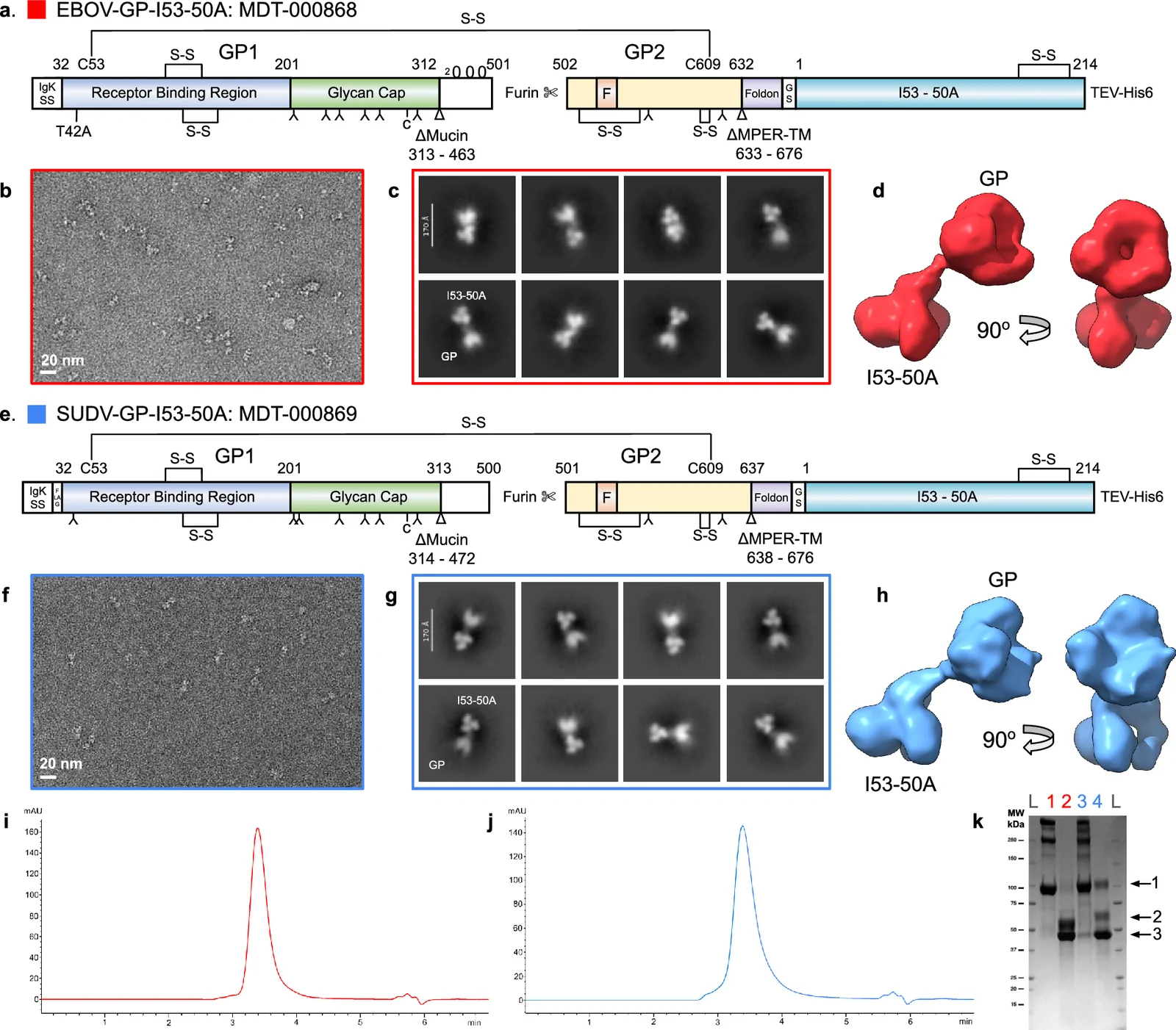
Production and nsEM structural characterization of EBOV-GP-I53-50A and SUDV-GP-I53-50A antigenic constructs. Secreted proteins were expressed by transient transfection in ExpiHEK293F cells with pCMV/R vectors and purified from serum free medium by IMAC and SEC and then characterized by nsEM, analytical SEC HPLC, and SDS-PAGE gels (see “Methods”). **a**–**d** EBOV-GP-I53-50A: MDT-000868 (red) (**a**) Protein architecture (**b**) NsEM micrograph (**c**) nsEM 2D class averages (**d**) 3D reconstruction, in C1 without symmetry applied. **e**–**h** SUDV-GP-I53-50A: MDT-000869 (blue) (**e**) Protein architecture (**f**) nsEM micrograph (**g**) nsEM 2D class averages (**h**) 3D reconstruction, in C1 without symmetry applied. **i**, **j** Final SEC pure proteins were characterized by analytical SEC HPLC (AdvanceBio SEC 300 Å) (**i**) EBOV-GP-I53-50A: MDT-000868 (red) (**j**) SUDV-GP-I53-50A: MDT-000869 (blue) (**k)** SDS-PAGE under non-reducing (NR, lanes 1 and 3) and reducing (R, lanes 2 and 4) conditions prior to being used for nanoparticle assemblies and animal immunization studies. Molecular weight markers in ladder (L) are shown; lanes 1 & 2 EBOV-GP-I53-50A MDT-000868 (red); lanes 3 & 4 SUDV-GP-I53-50A MDT-000869 (blue); Band 1: GP1-(∆Mucin)-GP2-(∆MPER-TM)-I53-50A; Band 2: GP1-(∆Mucin); Band 3: GP2-(∆MPER-TM)-I53-50A. Molecular weight marker in kilodaltons, MW kDa, left. Experiments completed once for each of these samples.

Next we focused on adapting the I53-50 nanoparticle to display the antigen for vaccination. We generated constructs encoding EBOV-GP-Foldon as an N-terminal fusion to the trimeric nanoparticle component I53-50A via a short flexible Gly-Ser linker. The descriptive name for this construct is “EBOV-GP-I53-50A” (MDT-000868, Fig. 2a). We initially produced and characterized this construct using small-scale lentiviral transduction in HEK293 cells^66^ and observed secretion of a uniform trimer that we purified by immobilized metal affinity chromatography (IMAC) and size exclusion chromatography (SEC) (Supplementary Fig. 8a, b). Using the same construct design strategy as EBOV, we next generated an I53-50A SUDV construct called SUDV-GP-I53-50A (MDT-000869, Fig. 2e). SUDV-GP-I53-50A was also produced and characterized using small-scale lentiviral transduction in HEK293 cells^66^. The secreted SUDV-GP-I53-50A was found to be a reasonably uniform trimer after purification by IMAC and SEC (Supplementary Fig. 8c,d). Negative stain EM (nsEM) characterization of both EBOV-GP-I53-50A and SUDV-GP-I53-50A trimers revealed the expected tertiary and quaternary geometries for the two-domain homotrimeric proteins. 2D and 3D refinements (refined in C1; i.e., without an applied symmetry) revealed the clear presence of GP trimers attached to I53-50A trimers. Importantly, no evidence of monomeric or unfolded trimeric species was observed, and despite the resolution limitations of nsEM, the GPs (∆muc, ∆MPER-TM, Foldon) of both EBOV and SUDV appeared well-behaved as genetic fusions to I53-50A (Supplementary Figs 8 and 9). See (Supplementary Data 1) for all protein sequences.

### I53-50 nanoparticle immunogen production and structural characterization

**Fig. 3:**
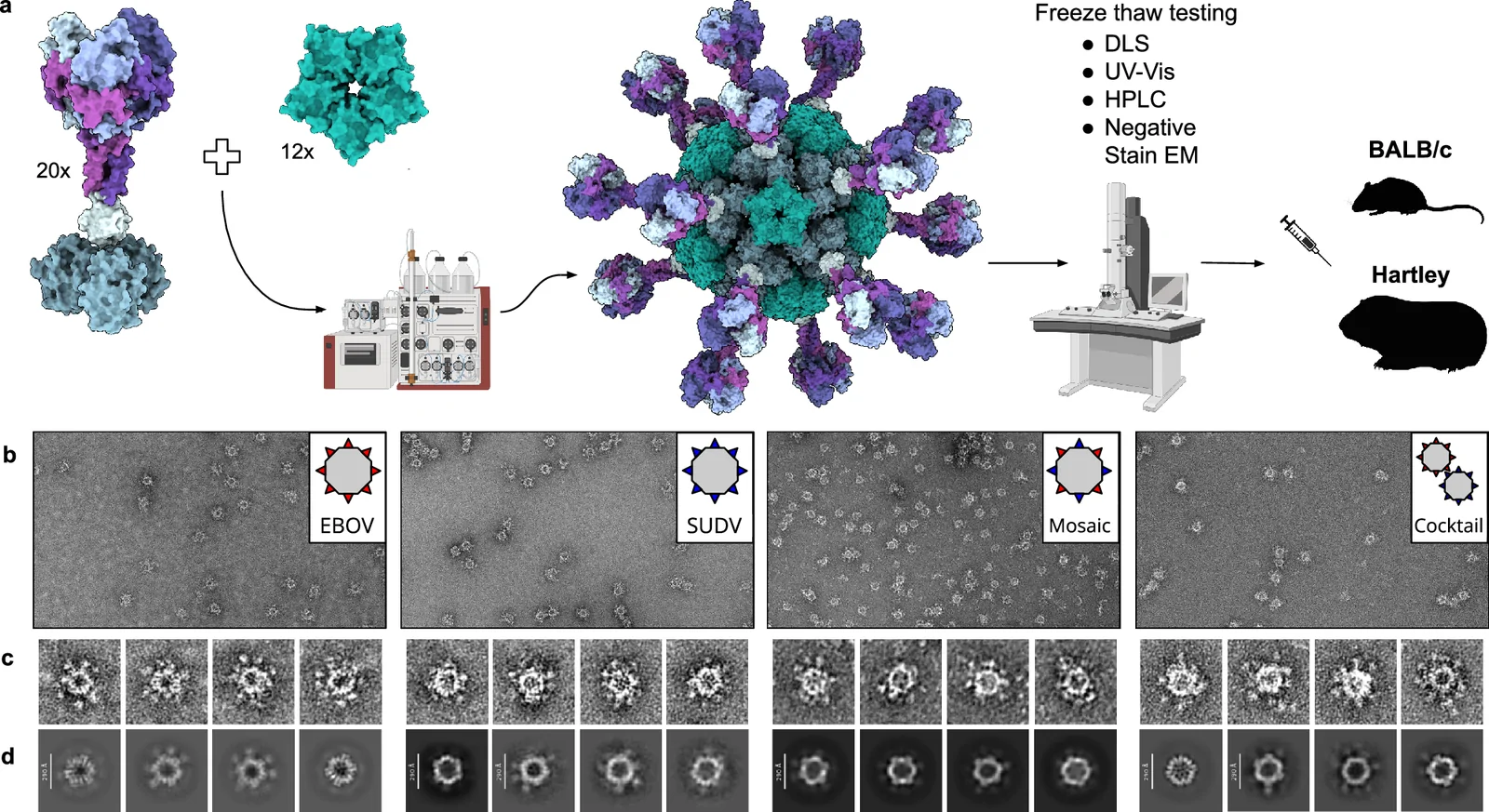
Production and nsEM structural characterization of EBOV- and SUDV-GP-I53-50A antigens as assembled I53-50 nanoparticles. **a** The 3D molecular representations of the structure of EBOV- and SUDV-GP-I53-50A trimeric antigens along with the pentameric I53-50B NP component. When mixed in solution at ~1:1 stoichiometric ratio, the proteins self-assemble into two-component icosahedral nanoparticles with molecular structure illustrated with the copy numbers noted. The assembled NP immunogens are then purified by SEC. Peak fractions are then aliquoted, flash frozen, and tested for particle uniformity both pre- and post-freeze thaw by dynamic light scattering (DLS), UV-Vis, analytical SEC HPLC, and nsEM. NsEM screening is performed at least twice for each of these samples (Supplementary Figs 11–13, 19, 24–26) prior to immunization studies in rodents. Additional 2D and 3D analyses (Supplementary Fig. 9b) were completed once for each of these samples (Created in BioRender. Weidle, C. https://BioRender.com/1cafznv and ChimeraX). **b**–**d** are arranged in columns and rows with: EBOV (red), SUDV (blue), Mosaic: EBOV & SUDV (red and blue), Cocktail: EBOV (red) & SUDV (blue) NP immunogens illustrated. **Row b**. The negative stain EM micrographs. **Row c** The 5Å Low pass filtered single particle images extracted from representative micrographs. **Row d** Representative 2D class averages generated from all selected particles in each dataset. The I53-50 icosahedral NPs are well resolved with visibly flexible GP components extending outwardly from the NP surface. **Column 1**, EBOV-GP-I53-50 NP. **Column 2**, SUDV-GP-I53-50 NP. **Column 3**, Mosaic NP where EBOV-GP-I53-50A and SUDV-GP-I53-50A are co-assembled with I53-50B. **Column 4**, Cocktail of EBOV-GP-I53-50 NP and SUDV-GP-I53-50 NP mixed post purification of individual nanoparticles. See (Supplementary Fig. 9b) for additional details on nsEM of these NP immunogens.

We next scaled up expression and purification of EBOV-GP-I53-50A and SUDV-GP-I53-50A by transient transfection of ExpiHEK293F cells using the pCMV/R vector, generating >10 milligrams of each construct following purification by IMAC and SEC (Supplementary Fig. 10). Both purified protein constructs had uniform analytical SEC-HPLC profiles and the expected sodium dodecyl sulfate-polyacrylamide gel electrophoresis (SDS-PAGE) band patterns (Fig. 2i–k). NsEM analysis confirmed that each I53-50A construct displayed well-folded, stable, trimeric prefusion GP glycoproteins following scaled-up production (Fig. 2a–h). Following previously established protocols for two-component nanoparticle assembly^39^, we mixed the GP-I53-50A trimers with pentameric I53-50B pentamer purified from *E. coli* to produce icosahedral NP immunogens displaying 20 GP trimers, followed by a final round of SEC purification to remove unassembled components (Fig. 3a). In total, we produced and characterized four types of GP-I53-50 NP immunogens in this way: EBOV-GP-I53-50 NP (Supplementary Fig. 11), SUDV-GP-I53-50 NP (Supplementary Fig. 12), a mosaic I53-50 NP co-displaying EBOV- and SUDV-GP that was assembled by adding I53-50B to an equimolar mixture of the EBOV-GP-I53-50A and SUDV-GP-I53-50A components (Supplementary Fig. 13), and a 1:1 “cocktail” mixture of EBOV-GP-I53-50 NP + SUDV-GP-I53-50 NP (Supplementary Fig. 14). Importantly, we have previously shown that the antigenic content of purified mosaic NPs displaying trimeric viral glycoproteins (influenza HA) matched the ratios of antigen-bearing components in the in vitro assembly reaction^48^. Each assembled NP sample in this study was further characterized by nsEM, DLS, UV-Vis, and HPLC, with nsEM showing individual nanoparticles with clearly visible prefusion GP trimers protruding flexibly from the particle surface. Furthermore, 2D class averages similarly showcased the expected I53-50 nanoparticle morphology with outward-facing flexible GP extensions (Fig. 3b–d; Supplementary Figs 11–14). Finally, 3D ab initio reconstructions and subsequent 3D refinements were performed for each sample, with the designed I53-50A nanoparticle design model agreeing well with each corresponding 3D density map following rigid-body docking (Supplementary Fig. 9b).

To assess thermal solution stability relevant to vaccine formulation and physiological temperature exposure, we conducted differential scanning fluorimetry under a thermal ramping regime while measuring the intrinsic tryptophan fluorescence of the protein samples (Supplementary Fig. 15). The I53-50A component remained stable with no detectable unfolding even at the upper temperature limit, whereas the I53-50B component showed a gradual transition beginning near 80 °C. However, when I53-50A and I53-50B were assembled into the full nanoparticle, no unfolding transitions were observed for either component, indicating enhanced stability of the assembled I53-50 NP relative to the free components. An initial melting transition (Tm₁ ≈ 55 °C) was observed for all GP and GP-containing NP immunogens, including EBOV-GP-Foldon (MDT-000759), EBOV-GP-I53-50A (MDT-000868), SUDV-GP-I53-50A (MDT-000869), EBOV-GP-I53-50 NP, SUDV-GP-I53-50 NP, EBOV/SUDV-GP-I53-50 Mosaic NP, and EBOV + SUDV I53-50 Cocktail NP. Constructs with the foldon trimerization domain displayed an additional secondary transition near 80 °C, while addition of I53-50A shifted this secondary event upward to ~88 °C. This biphasic behavior was retained upon nanoparticle assembly with I53-50B. We interpret these transitions as representing sequential destabilization of the GP trimer at ~55 °C^20,43^ and potential unfolding of either a postfusion state or individual GP1/GP2 monomers at higher temperature^72,73^ (Supplementary Fig. 15). These results demonstrate that all EBOV and SUDV constructs retained structural integrity at temperatures well above physiological levels–results consistent with previous studies of GP and nanoparticle displayed GP immunogens^41,43^–indicating that they likely remain structurally stable during immunization.

### Prophylactic immunization and challenge with mouse-adapted EBOV

**Fig. 4:**
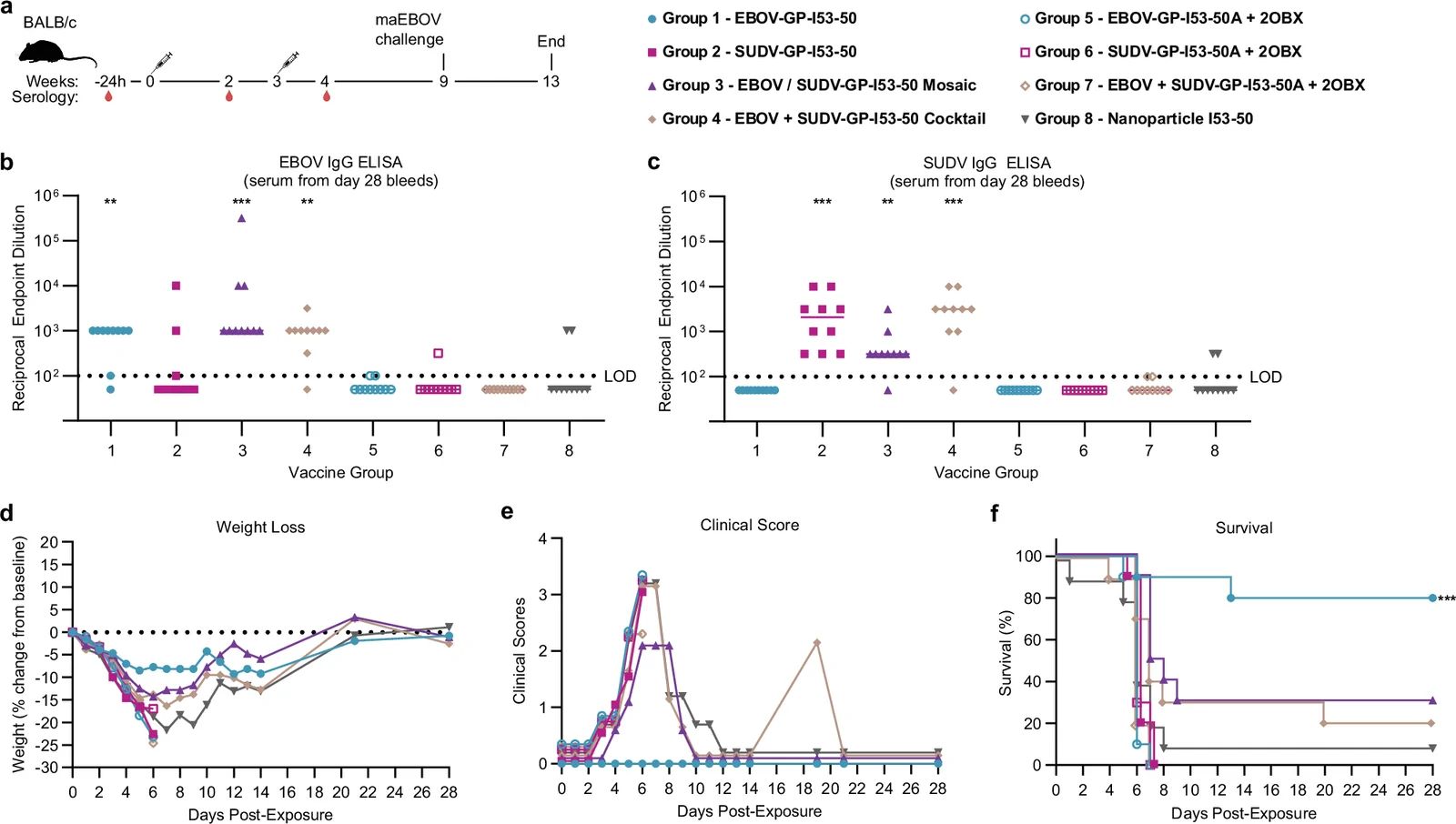
Prophylactic immunization and challenge with the mouse adapted EBOV. **a** The study protocol is illustrated. Serum was collected from mice the day prior to the prime dose, two weeks following the prime dose, and one week following the boost dose at week 3. At week 9, animals were transferred to BSL-4 facilities and challenged with 100 pfu of maEBOV by intraperitoneal (IP) route, after which all animals were monitored for moribundity and weight loss as a sign of EVD for 28 days following viral challenge. Test article descriptions are noted in the legend with colorized dots for each group (10 animals per group) (Created in BioRender. Weidle, C. https://BioRender.com/1cafznv). **b** Anti-EBOV GP or **c** anti-SUDV GP IgG antibody titers from day 28 post-boost blood collections were measured by ELISA following serial dilution to establish the Y-Axis, Log10 plot of the reciprocal endpoint of dilution needed to observe signal in the assay relative to baseline pre-immunization serum signals. **d** The average daily percentage of weight loss or gain over time was measured for each group of animals following viral challenges. Mean values with +/− SEM and N sample size in source data file. **e** Average daily clinical disease scores were monitored for each group of animals following viral challenges. See methods for clinical disease score definitions. Mean values with +/− SD and N sample size in the source data file. **f** Kaplan–Meier survival curves, where moribund mice were promptly euthanized when they met predefined euthanasia criteria, a score of 4; defined as bleeding, unresponsive, weak, or unable to walk. Legend for Groups is indicated in the upper right. For survival, *P* values are indicated for comparisons against the respective bare I53-50 control Group 8, based on log-rank Mantel-Cox statistical analyses, *P < 0.05; **P < 0.01; ***P < 0.001.

Having confirmed the antigenic structural integrity of our EBOV- and SUDV-GP-I53-50 NP immunogens, we next conducted a prime-boost mouse immunization study followed by challenge with maEBOV^74^ in the USAMRIID BSL-4 facilities (Fig. 4a). The study included 8 groups (10 mice per group), and all test articles were formulated with an equal volume of AddaVax™, a squalene-based oil-in-water adjuvant, prior to subcutaneous injection. The four main test groups included EBOV-GP-I53-50 (Group 1), SUDV-GP-I53-50 (Group 2), the mosaic EBOV / SUDV-GP-I53-50 NP (Group 3), and the 1:1 cocktail of EBOV-GP-I53-50 and SUDV-GP-I53-50 NPs (Group 4). For each group, 3.4 µg of total protein was injected equating to 1.9 µg of GP antigen. To investigate the importance of nanoparticle assembly in eliciting anti-GP antibody responses, we also included matched “non-assembling” control Groups 5–7 using 2.5 µg of GP-I53-50A trimer (1.6 µg GP antigen) mixed with a pentameric protein called “2OBX” (0.8 µg)^75,76^. 2OBX is nearly identical to the I53-50B pentamer yet is unable to drive assembly of I53-50 NPs, as it lacks the designed protein-protein interface required for interaction with I53-50A^38^ (Supplementary Figs 16–18). As a negative control immunogen (Group 8), we also injected animals with bare I53-50 NP (2.5 µg) lacking any GP antigen (Supplementary Fig. 19). 100 µL volume was injected for all groups.

Serological analysis at day 28 (7 days post-boost) showed strong antibody titers to EBOV GP in Group 1 and to SUDV GP in Group 2 (Fig. 4b, c), as measured by ELISAs using full-length GP ectodomains containing the mucin-like and MPER regions to mimic the native antigenic surface encountered during infection (Supplementary Fig. 20). Little or no cross-reactive antibody responses were observed in this assay, with only 3 out of 10 animals that received EBOV-GP-I53-50 demonstrating any observable anti-SUDV-GP antibodies. Furthermore, none of the ten animals that received SUDV-GP-I53-50 had any appreciable anti-EBOV-GP antibodies. The EBOV and SUDV GP components of our NP immunogens share only 68% sequence identity (Supplementary Figs 21 and 22), which in this maEBOV mouse model, appears to be insufficient to elicit strong cross-reactive antibody responses. Notably, animals immunized with either the mosaic (Group 3) or cocktail (Group 4) immunogens elicited strong antibody responses against both EBOV-GP and SUDV-GP, with only one and zero of the animals in Groups 3 or 4, respectively, lacking an immune response to either EBOV-GP or SUDV-GP. As evidence that antigen display in the form of a nanoparticle is important for eliciting potent immune responses, the non-assembling controls of EBOV-GP-I53-50A with 2OBX (Group 5), SUDV-GP-I53-50A with 2OBX (Group 6), as well as the equal mixture of EBOV- and SUDV-GP-I53-50A with 2BOX (Group 7) did not elicit strong antibody responses (e.g., compare Group 1–5 or Group 2–6). Sera from two of the Group 8 mice receiving the bare I53-50 nanoparticle had low but detectable anti-EBOV-GP and anti-SUDV-GP IgG titers, which we suspect may represent a reaction with the His tags present on each construct.

To determine if the immunogens were capable of protecting animals from maEBOV challenge^77^, five weeks after the booster vaccination, all animals were exposed to 100 plaque forming units (pfu) of maEBOV by intraperitoneal (IP) injection, and then monitored daily for signs of disease (morbidity is defined clinical disease score (see: methods)). Kaplan–Meier analysis showed that mice immunized with EBOV-GP-I53-50 (Group 1) had a statistically significant survival advantage (*p* < 0.001), with reduced weight loss, improved clinical scores, and higher survival compared with all other groups (Fig. 4d–f). Some of the mice receiving the mosaic EBOV/SUDV-GP-I53-50 NP (Group 3) or cocktail EBOV-GP-I53-50 + SUDV-GP-I53-50 (Group 4) were also protected from challenge, although the differences were not statistically significant compared to the negative control immunogen (bare I53-50 NP; Group 8). Poor survival was observed in mice receiving only SUDV-GP-I53-50 (Group 2) or the non-assembling control immunogens (Groups 5–7), with all animals succumbing by day 8 post-challenge. One animal in the bare I53-50 control group (Group 8) survived, but it was not among the two mice with detectable anti-EBOV GP antibodies (Fig. 4b). The mosaic (Group 3) and cocktail (Group 4) formulations generated statistically significant anti-EBOV or anti-SUDV GP IgG responses relative to bare-NP controls (Fig. 4b, c), yet only three and two animals survived, respectively. Although these survival outcomes were not significantly different from bare NP control group (Group 8), they were statistically significant relative to the SUDV-GP-I53-50 group (Group 2) for both mosaic (*p* < 0.001) and cocktail (*p* < 0.01) immunizations (Supplementary Fig. 23a). Collectively, these findings show that EBOV-GP displayed on the I53-50 nanoparticle is capable of eliciting protective anti-EBOV immunity in mice, whereas the non-assembling trimers do not. In contrast, although SUDV-GP-I53-50 induced strong anti-SUDV antibody responses, it did not provide cross-protection when mice were challenged with maEBOV. Moreover, the mosaic and cocktail formulations, while achieving statistically significant survival relative to the SUDV-GP-I53-50 group, provided unexpectedly mild protection compared to the strain-matched EBOV-GP-I53-50 nanoparticle in mice.

### Prophylactic immunization and challenge with the Guinea Pig-adapted SUDV

**Fig. 5:**
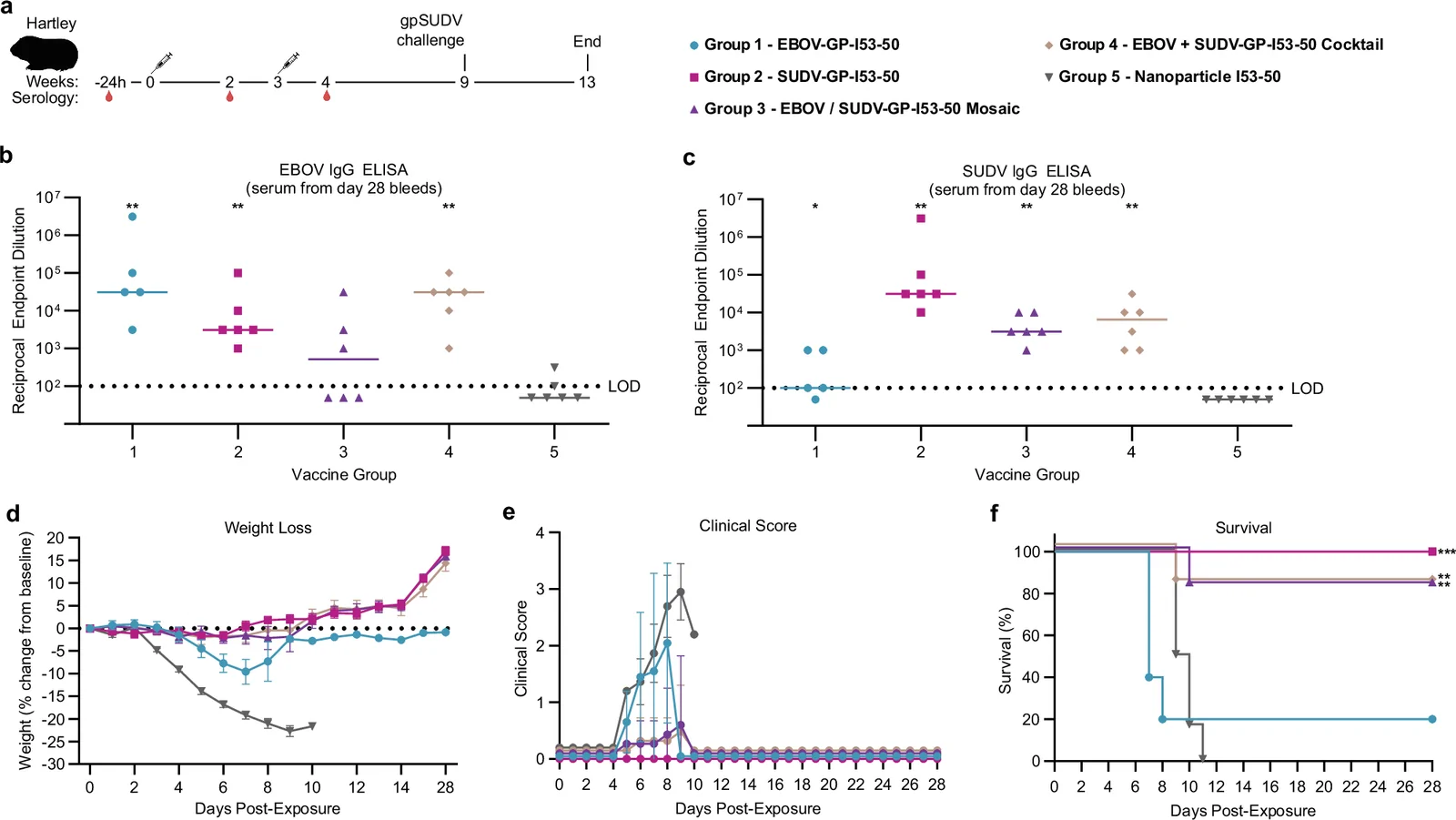
Prophylactic immunization and challenge with the guinea pig adapted SUDV. **a** The study protocol is illustrated. Serum was collected from guinea pigs the day prior to a prime dose, two weeks following the prime dose, and one week following the boost dose at week 3. At week 9, animals were transferred to BSL-4 facilities and challenged with 100 pfu of gpaSUDV by IP route after which animals were monitored for weight loss as a sign of EVD for 28 days following viral challenge. Test article descriptions are noted in the legend with colorized dots for each group (6 animals per group, except for Group 1, which had 5 animals) (Created in BioRender. Weidle, C. https://BioRender.com/1cafznv). **b** Anti-EBOV GP or (**c**) anti-SUDV GP IgG antibody titers from day 28; post-boost blood collections were measured by ELISA following serial dilution to establish the *Y*-Axis, Log10 plot of the reciprocal endpoint of dilution needed to observe signal in the assay relative to baseline pre-immunization serum signals. **d** Average daily percentage of weight loss or gain over time following challenges (weights for each animal were measured daily). Mean values with +/− SEM and N sample size in source data file. **e** Average daily clinical disease scores over time following viral challenges (clinical scores for each animal were measured daily); see “Methods” for clinical disease score definitions. Mean values with +/− SD and N sample size in the source data file. **f** Kaplan–Meier survival curves, where moribund guinea pigs were promptly euthanized when they met predefined euthanasia criteria, a score of 4; defined as bleeding, unresponsive, weak, or unable to walk. Legend for Groups is indicated in the upper right. For survival, *P* values are indicated for comparisons against the respective bare I53-50 control Group 5, based on log-rank Mantel-Cox statistical analyses, *P < 0.05; **P < 0.01; ***P < 0.001.

Having established that the EBOV-GP-I53-50 nanoparticle immunogen can elicit protective immune responses in the maEBOV model, we conducted a follow-up prophylactic prime-boost immunization study with Hartley guinea pigs, including challenge with guinea pig-adapted SUDV (gpaSUDV)^78^ in the USAMRIID BSL-4 facilities (Fig. 5). Groups of 6 animals (except for Group 1 which had 5 animals) were immunized with newly prepared and characterized nanoparticle immunogens, receiving 2.4 µg of GP antigen (4.3 µg total protein) (Supplementary Figs 24–26). Since the prior maEBOV study had already demonstrated poor immune response with the non-assembling 2OBX control groups, we did not include the non-assembling 2OBX groups in this gpaSUDV study. As the negative control immunogen, we injected animals with 2.4 µg of bare I53-50 NP lacking any GP antigen (Group 5). All test articles were formulated with an equal volume of AddaVax™ prior to subcutaneous injection, to maintain continuity with the prior mouse study parameters. 250 µL injection volume was used for all groups.

As observed in the previous mouse study, serological responses in guinea pigs at 7 days post boost (day 28) demonstrated strong vaccine-matched IgG antibody titers to EBOV-GP in Group 1 or SUDV-GP in Group 2 (Fig. 5a–c). EBOV-GP-I53-50 immunization generated statistically significant low-level cross-reactive IgG antibody responses to SUDV-GP in two guinea pigs, as compared to no cross-reactivity in mice (Fig. 4c, Fig. 5c). In contrast, the SUDV-GP-I53-50 NP (Group 2) was able to produce reliable cross-reactive antibody responses to EBOV-GP in guinea pigs (Fig. 5b), a finding consistent with prior multivalent GP vaccine studies using HPIV3 vectors expressing EBOV, SUDV, and BDBV GPs^79^. The mosaic (Group 3) and cocktail (Group 4) immunogens both elicited anti-GP IgG responses to both EBOV-GP and SUDV-GP, although anti-EBOV-GP IgG titers were only detectable in three of the six Group 3 animals. None of the Group 5 animals receiving the bare I53-50 negative control had any appreciable anti-GP IgG.

At five weeks following the booster immunization, all animals were transferred to BSL-4 and dosed with IP injections of 1000 plaque-forming units (pfu) of gpaSUDV. Animals immunized with any I53-50 NP bearing the SUDV-GP antigen (Groups 2–4) had an 83–100% survival rate at 28 days post-infection (Fig. 5d–f); morbidity is defined by clinical disease score (see: methods)). Furthermore, these animals remained asymptomatic or demonstrated only mild symptoms, as measured by temperature (Supplementary Fig. 27) and overall clinical disease score (Fig. 5e). By contrast, animals immunized with EBOV-GP-I53-50 (Group 1) or the bare I53-50 NP (Group 5) had limited survival rates and showed high levels of clinical disease. All of the Group 5 control animals succumbed by day 11, and only one of the guinea pigs in Group 1 recovered 9 days after infection. Comparing Kaplan–Meier survival curves indicated that a statistically significant protective advantage was afforded to the animals receiving SUDV-GP-I53-50 (Group 2; *p* < 0.001), the mosaic EBOV/SUDV-GP-I53-50 NP (Group 3; *p* < 0.01), or the EBOV-GP-I53-50 + SUDV-GP-I53-50 NP cocktail (Group 4; *p* < 0.01) compared to the control group treated with bare I53-50 (Group 5). The mosaic (Group 3) and cocktail (Group 4) guinea pigs displayed a statistically significantly higher anti-EBOV GP or anti-SUDV GP IgG response relative to bare I53-50 controls (Fig. 5b, c). Five of the six guinea pigs in each of these groups respectively survived gpaSUDV challenge, a significantly better survival for mosaic (Group 3, *p* < 0.05) or cocktail (Group 4, *p* < 0.01) when compared to bare I53-50 NP control group (Group 5) or the EBOV-GP-I53-50 vaccinated animals (Group 1) (Supplementary Fig. 23b). Overall, survival outcomes in the gpaSUDV guinea pig model were markedly better than those observed in the maEBOV mouse challenge (Fig. 4f, Fig. 5f). Mosaic and cocktail EBOV/SUDV-GP-I53-50 NP immunogens protected 5 of 6 animals in each group (83% survival), and the strain-matched SUDV-GP-I53-50 NP conferred complete protection; all substantially outperforming the 20–30% survival seen in the maEBOV study’s mosaic and cocktail GP-I53-50 groups. These findings highlight the strong protective capacity of SUDV-GP–bearing, mosaic, and cocktail I53-50 NP immunogens in a stringent BSL-4 guinea pig model and underscore their potential value as broadly protective filovirus vaccine candidates (Supplementary Fig. 23).

## Discussion

In this study we sought to design and test the efficacy of I53-50 NP vaccines for multivalent display of either EBOV-GP or SUDV-GP, as well as mosaic and cocktail versions of the NP immunogens. A growing body of prophylactic vaccination data from both animal models and in people has previously shown that vaccines with only EBOV-GP or SUDV-GP immunogens alone are not capable of eliciting cross-Ebolavirus anti-GP immune responses, and therefore do not offer broad-spectrum EVD protection^20,21^. Using a prime-boost vaccination strategy, our individual EBOV-GP-I53-50 or SUDV-GP-I53-50 NP immunogens, formulated with AddaVax™ adjuvant, provided protection from death and weight loss, which correlated with detectable anti-GP IgG serum responses. This was true for homologous viral challenge in BSL-4 models for both mouse-adapted EBOV (maEBOV) and with guinea pig-adapted SUDV (gpSUDV). In our maEBOV study, we compared the antibody responses and protection provided by the GP-I53-50 NP immunogens to identical quantities of soluble trimeric GP-I53-50A mixed with a non-assembling 2OBX pentamer. The weak antibody responses and lack of survival in the groups of mice receiving the non-assembling 2OBX control immunogens demonstrate that multimeric NP display of the GP antigen aids in to eliciting a strong and protective antiviral immune response, as has been observed for influenza, SARS-CoV2, RSV, EBV and HIV viral antigens^34,48,50,80–82^. Cocktail and mosaic EBOV/SUDV GP I53-50 nanoparticles generated strong antibody responses and provided protection in both BSL-4 rodent models, although their performance relative to strain-matched single-antigen immunogens varied considerably by challenge system, with the cocktail and mosaic formulations performing more favorably in the guinea pig model. Ultimately, strain-matched immunogens gave the highest survival in both models, and antibody cross-reactivity following single-antigen immunization was observed only in guinea pigs, not in mice. Together, these findings highlight that the guinea pig model demonstrates broader cross-reactivity and more robust heterologous protection than the maEBOV mouse model, aligning more closely with immunogenicity patterns observed for E2p NP EBOV GP trimers in immunized rabbits^42^.

While multivalent protein-based Ebola vaccine candidates have been reported using full-length GP purified from insect cells as rosette aggregates^83,84^, or as single-component nanoparticle vaccine candidates based on I3-01^42,75^, E2p^42,43^, or ferritin^41^, these nanoparticle systems lack the ability to easily co-display multiple antigens. However, this can be readily accommodated by the two-component I53-50 NP. Mosaic nanoparticles can alternatively be produced using the SpyTag/SpyCatcher system^85,86^. A SARS-CoV-2 vaccine based on the two-component nanoparticle scaffold used in this study was licensed for use in both the United Kingdom and South Korea and was granted Emergency Use Listing by the World Health Organization in 2023^36,37,87^. The I53-50 NP has a growing safety profile with multiple clinical trials completed for SKYCovione™ and RSV/HMPV^50^, demonstrating that scalable manufacturing of I53-50-based vaccines is not likely to present a roadblock to further clinical development of the EBOV- and SUDV-GP-I53-50 vaccines. In our hands, the production EBOV-GP-I53-50A and SUDV-GP-I53-50A components was about four-fold lower (~7.3 and 7.6 mg/L by transient transfection, respectively (Supplementary Fig. 10) than that observed for SARS-CoV-2 RBD-I53-50A fusion proteins^35^. In addition, the GP-containing components of our EBOV-GP-I53-50A and SUDV-GP-I53-50A immunogens are thermostable up to an initial melting transition of 55 °C. Further thermostability could potentially be achieved through stabilizing mutations in GP^42,88^, or via drosophila-based expression systems and lyophilization^15,89^. Collectively, these results position the EBOV- and SUDV-GP-I53-50 nanoparticle immunogens as potentially promising candidates for the continued development of broadly protective ebolavirus vaccines.

In this study, we generated a high-resolution cryoEM structure of the pre-fusion trimeric EBOV GP in the absence of any binding partners; offering new insights which may help support ongoing antiviral protein minibinder design and small-molecule drug discovery. While previous EBOV GP structures have been solved in the apo state by X-ray crystallography^20,42,63^, all reported structures were generated under similar crystallization conditions. These include the use of similar buffers (PEG, sodium citrate), crystal packing in space groups H32 and P321 (with a shared crystal packing interface near inhibitor binding site), and a pH range spanning 5.0–5.2 (Supplementary Fig. 7a)^20,42,63^. These conditions consistently produced crystal contacts proximal to a known small-molecule binding pocket located between the GP1 and GP2 subunits. In prior apo X-ray structures, this pocket is occupied by F193 and F194, which are displaced in GP1–GP2 complexes with small-molecule inhibitors known to block viral entry and thermally destabilize GP^63,65,67–69^. In our apo-state cryoEM structure of EBOV GP at pH 7.5, we also observe F193 and F194, but their spatial configurations and the surrounding pocket differ from those seen in prior apo crystal structures (Supplementary Fig. 7). These structural differences in the cryo-EM model likely reflect the solution-state conformation of the EBOV GP small-molecule binding pocket under physiological conditions, an observation which is potentially noteworthy for any drug design efforts targeting this site.

Additionally, the glycan cap (residues 224–310) was unresolved in our cryoEM structure. This was an interesting observation, as this domain is resolved in the aligned crystal structure sharing the same sequence as (PDB ID: 5JQ3). We hypothesize that this was likely due to stabilizing crystal packing effects for these domains. An alternative hypothesis for a lack of observable glycan cap density in our map could be the presence of a cathepsin cleavage site at Lys190, which removes the glycan cap and mucin domains to facilitate viral entry^90–92^. However, clear density in our map for residues 191–195 and 216–223 indicates that no cleavage had occurred at this site – a result consistent with our SDS–PAGE analysis (Fig. 1d). Therefore, the absence of glycan cap density in the cryoEM maps likely reflects the weak attachment and high degree of flexibility of this region relative to the GP core domains, as previously hypothesized by other groups^70,93^ that also failed to observe glycan cap density in their antibody-bound cryoEM structures. Notably, the absence of the mucin-like domain may further contribute to presumed flexibility of the glycan cap in our structure, with the mucin-like domain having been previously resolved to low resolution on virions and VLPs using cryoEM^94,95^.

There are several limitations to the studies reported here, mainly due to limited sera and the logistical constraints associated with conducting repeat BSL-4 studies. Our serological analyses of anti-GP IgG responses were based purely on ELISA assays. Furthermore, we have not examined the epitope specificities or structures of monoclonal or polyclonal antibodies elicited by our NP immunogens and therefore do not know if similar antibodies are induced by the mosaic and cocktail versions of our GP-I53-50 NP immunogens. In addition, our NP immunogens lacked the MPER region of GP, which is known to be targeted by broadly neutralizing antibodies^96–98^. However, display of an MPER-based epitope scaffold on the computationally designed two-component I53-40 nanoparticle failed to elicit neutralizing antibodies after rabbit immunization^99^. It remains to be determined if inclusion of the MPER domains in prefusion-stabilized trimeric GPs displayed on nanoparticles would further improve cross-reactive vaccine-elicited antibody responses. Future research would also benefit from an exploration of the nature of T cell responses to our GP-I53-50 NP immunogens^35^, the removal of His tags from the GP-NP immunogens, and dose range or prime-boost optimization of the mosaic or cocktail NP immunogens (versus their non-NP/assembled counterparts) for improved broad spectrum EBOV - SUDV protection.

Our results show that rationally designed EBOV and SUDV GP I53-50 nanoparticle immunogens form well-behaved, properly folded prefusion trimers that assemble efficiently and elicit strong protective immunity in homologous challenge models. The mosaic and cocktail formulations induced cross-reactive antibody responses in both mice and guinea pigs and provided clear protection against gpSUDV challenge in guinea pigs, supporting continued development of multivalent GP I53-50 nanoparticles for broader filovirus coverage. Additional refinement of antigen composition, adjuvant formulation, immunization strategy, and evaluation in nonhuman primate models will further inform and strengthen the development of EBOV and SUDV GP I53-50 vaccines.

## Methods

### Ethics statement

All animal studies were conducted under IACUC-approved protocols in compliance with the Animal Welfare Act, Public Health Service Policy, and other applicable federal statutes and regulations relating to animals and experiments involving animals. The facility where these studies was conducted (USAMRIID) is accredited by the Association for Assessment and Accreditation of Laboratory Animal Care, International and adheres to principles stated in the Guide for the Care and Use of Laboratory Animals, National Research Council, 2011. The animal study protocol was approved by the Institutional Animal Care and Use Committee of USAMRIID (protocol number AP-19-005, 05 February 2019). Enrichment was provided as recommended by the Guide for the Care and Use of Laboratory Animals. Research was conducted under Institutional Animal Care and Use Committee (IACUC) approved protocols in compliance with the Animal Welfare Act, PHS Policy, and other Federal statutes and regulations relating to animals and experiments involving animals. The facility where this research was conducted is accredited by AAALAC International and adheres to the principles stated in the Guide for the Care and Use of Laboratory Animals, National Research Council, 2011. All efforts were made to minimize pain and distress. Sample size was selected based on statistical analysis of the animal number needed to produce a statistically significant result paired with animal welfare consideration of reduction and cost considerations.

### Production of EBOV and SUDV glycoprotein immunogens by lentiviral transduction

EBOV-GP ManoRiver (MDT-000253); EBOV-GP-I53-50A (MDT-000868); SUDV-GP-I53-50A (MDT-000869); and EBOV-GP-Foldon (MDT-000759) constructs (see detailed sequences in Supplementary Data 1) were expressed as a soluble secreted proteins by lentiviral transduction using the Daedalus expression method^66^ using HEK293F (Invitrogen) cells grown in the presence of kifunensine to limit N-glycan heterogeneity. The secreted proteins were captured from conditioned media using a HisTrap FF crude column and eluted in PBS containing 250 mM imidazole. Each protein was then polished using a Superdex 200 Increase 10/300 GL column on an AKTA Pure M (GE) using a phosphate buffered saline (PBS) mobile phase. SDS-PAGE analysis was performed using NuPAGE 4–12% Bis-Tris gels run under non-reducing or reducing conditions where samples are treated with 1 mM dithiothreitol (DTT) prior to electrophoresis using manufacturer’s protocols. Final production yields were calculated based on absorbance determined by measuring absorbance at 280 nm using a UV-Vis spectrophotometer (Agilent Cary 8454) and calculated extinction coefficients^100^.

### Production of EBOV and SUDV glycoprotein immunogens by transient transfection

Proteins were produced with pCMV/R vectors^101^ with Xba1 and AvrII restriction sites using Gibson assembly^102^ using endotoxin-free DNA in ExpiHEK293F cells grown in suspension using Expi293F expression medium (Life Technologies) at 33 °C, 70% humidity, 8% CO_2_ rotating at 150 revolutions per minute (rpm). The cultures were transfected using PEI-MAX (Polyscience) with cells grown to a density of 3.0 million cells per mL and cultivated for 3 days. Supernatants were clarified by centrifugation (5 min at 4000 × *g*), addition of Poly(diallyldimethylammonium chloride) (PDADMAC) solution to a final concentration of 0.0375% (Sigma Aldrich, #409014), and a second centrifugation (5 min at 4000 × *g*). Similar methods as noted above for purification of constructs by IMAC and SEC were applied to the purification of GP immunogens from culture medium following the transient transfections. Final production yields were calculated based on absorbance determined by measuring absorbance at 280 nm using a UV-Vis spectrophotometer (Agilent Cary 8454) and calculated extinction coefficients^100^.

### Microbial plasmid construction, protein expression and purification of Tagless I53-50B.4PT1 Tagless

I53-50B.4PT1 plasmid^39^ was synthesized by GenScript in pET29b between the NdeI and XhoI restriction sites with a double-stop codon. Protein was expressed in Lemo21(DE3) cells (NEB) in LB (10 g Tryptone, 5 g Yeast Extract, 10 g NaCl) grown in a 10 L BioFlo 320 Fermenter (Eppendorf). At inoculation, impeller speed was set to 225 rpm, sparge per liter per minute (SPLM) set to 5 with O_2_ supplementation as part of the dissolved-oxygen aeration cascade, and the temperature set to 37 °C. At the onset of a dissolved oxygen (DO) spike (OD600 ~ 12), the culture was supplemented with a bolus addition of 100 mL of 100% glycerol and induced with 1 mM Isopropyl β-D-1-thigalactopyranoside (IPTG). During this time, the culture temperature was reduced to 18 °C, and O_2_ supplementation ceased, with expression continuing until an OD600 ~ 20. The culture was harvested by centrifugation, and the pellets were resuspended in Phosphate Buffered Saline (PBS), homogenized, and then lysed by microfluidization using a Microfluidics M110P at 18,000 pounds per square inch (psi) using 3 discrete passes. Following sample clarification by centrifugation (24,000 *g* for 30 min), the supernatant was discarded, and protein was extracted from the inclusion bodies. First, the pellet was washed with PBS and supplemented with 0.1% Triton X-100, pH 8.0. After this initial wash and sample clarification by centrifugation, the pellet was washed with PBS supplemented with 1 M NaCl, pH 8.0. Following the second wash, the protein was extracted from the pellet into PBS supplemented with 2 M urea and 0.75% CHAPS (3-[(3-Cholamidopropyl)dimethylammonio]-1-propanesulfonate), pH 8.0. Following extraction, the sample was applied to a DEAE Sepharose FF column (Cytiva) on an ӒKTA Avant150 FPLC system (Cytiva). After sample binding, the column was washed with 5 column volumes (CV) of PBS supplemented with 0.1% Triton X-100, pH 8.0, followed by a 5 CV wash with PBS supplemented with 0.75% CHAPs, pH 8.0 in series. The protein was eluted with 3 CV of PBS supplemented with 500 mM NaCl, pH 8.0. After purification, fractions were pooled and concentrated in 10 K molecular weight cutoff (MWCO) centrifugal filters (Millipore), sterile filtered (0.22 μm), and tested to confirm low endotoxin levels before use in nanoparticle assembly.

### Microbial plasmid construction, protein expression and purification His6 Tagged I53-50B.4PT1

The gene for I53-50B.4PT1 with a C-terminal hexa-histidine affinity tag was synthesized by GenScript in pET29b between the NdeI and XhoI restriction sites with a double-stop codon, and its microbial production and purification was identical to that of the 2OBX non-assembling pentamer.

### Microbial plasmid construction, protein expression and purification 2OBX Non-Assembling Pentamer

2OBX plasmid was synthesized by GenScript in pET29b between the NdeI and XhoI restriction sites in frame of the C-terminal polyhistidine tag^38^. The 2OBX^76^ pentamer was expressed in Lemo21(DE3) (NEB) in LB (10 g Tryptone, 5 g Yeast Extract, 10 g NaCl) grown in 2 L baffled shake flasks. Cells were grown at 37 °C to an OD600 ∼0.8 and then induced with 1 mM IPTG. Expression temperature was reduced to 16 °C and the cells were shaken for ∼16 h. The cells were harvested and lysed by microfluidization using a Microfluidics M110P at 18,000 psi in 25 mM Tris, 500 mM NaCl, 30 mM imidazole, 1 mM phenylmethylsulfonyl fluoride (PMSF), 0.01 mg/mL DNAse, and 0.75% CHAPS. Lysates were clarified by centrifugation at 24,000 g for 30 min and applied to 5 mL of NiNTA resin (Qiagen) for purification by gravity IMAC. Protein of interest was eluted using 25 mM Tris pH 8, 500 mM NaCl, 0.75% CHAPS, 500 mM imidazole buffer. The elution was pooled, concentrated in 10 K MWCO centrifugal filters (Millipore), sterile filtered (0.22 μm) and applied to a Superdex 200 Increase 10/300 using 25 mM Tris pH 8, 500 mM NaCl, 0.75% CHAPS buffer. After sizing, the component was tested to confirm low levels of endotoxin before using it for animal vaccination studies as part of test articles.

### Microbial plasmid construction, protein expression and purification of I53-50A

The I53-50A plasmid was synthesized by GenScript in pET29b between the NdeI and XhoI restriction sites in frame of the C-terminal polyhistidine tag^38^. Expression plasmids were transformed into BL21(DE3) E. coli cells. Cells were grown in LB medium supplemented with 50 mg/L of kanamycin (Sigma) at 37 °C until an OD600 of 0.8 was reached. Protein expression was induced by addition of 0.5 mM isopropyl-thio-β-D-galactopyranoside (Sigma) and allowed to proceed for 3 h at 37 °C before cells were harvested by centrifugation^38^. Cells were lysed by sonication (2.5 min total sonicating time in 2 s pulses) in 50 mM Tris pH 8, 500 mM NaCl, 20 mM Imidazole, 0.75% 3-[(3-Cholamidopropyl)dimethylammonio]-1-propanesulfonate (CHAPS), 0.1 mg/mL lysozyme, 0.05 mg/mL DNase, 0.05 mg/mL RNase, 5 mM MgCl_2_ and 1 mM PMSF. Lysate was clarified by centrifugation at 33,000 × g for 20 min. Lysate supernatants were applied to HisTrap HP or FF columns (GE Healthcare) for purification by immobilized metal affinity chromatography (IMAC) on an AKTA Pure FPLC system (GE Healthcare). Protein of interest was eluted over a linear gradient of 20 mM to 500 mM imidazole in a background of 50 mM Tris pH 8, 500 mM NaCl, and 0.75% CHAPS buffer after washing with ∼10 column volumes wash buffer (elution buffer with 20 mM imidazole). Peak fractions were pooled, concentrated in 10 K MWCO centrifugal filters, sterile filtered (0.22 μm) and applied to a Superdex 200 Increase 10/300 GL SEC column (GE Healthcare) using 25 mM Tris pH 8, 500 mM NaCl, 0.75% CHAPS buffer, 1 mM DTT. I53-50A elutes at ∼15–17 mL.

### Assembly production and purification of EBOV and SUDV glycoprotein I53-50 nanoparticles

Total protein concentration of purified individual nanoparticle components was determined by measuring absorbance at 280 nm using a UV/vis spectrophotometer (Agilent Cary 8454) and calculated extinction coefficients^100^. The assembly steps were performed at room temperature with addition in the following order: EBOV-GP-I53-50A or SUDV-GP-I53-50A trimeric glycoprotein, followed by quality sufficient (q.s.) volume filling with buffer as needed to achieve desired final concentration, and finally either tagless or hexa-histidine tagged I53-50B.4PT1 pentameric component (in 50 mM Tris pH 8, 500 mM NaCl, 0.75% w/v CHAPS), with a molar ratio of GP-I53-50A: I53-50B.4PT1 of 1.1:1. q.s. buffer contained 50 mM Tris pH 8, 150 mM NaCl, 5% v/v glycerol. A Superose 6 Increase 10/300 GL column was used for nanoparticle purification with running buffer as 25 mM Tris pH 8, 150 mM NaCl, 5% v/v glycerol for NPs prepared for the maEBOV study (Fig. 4) or 50 mM Tris pH 8, 150 mM NaCl, 5% glycerol, 100 mM L-arginine for NPs prepared for the gpaSUDV study (Fig. 5). Assembled nanoparticles were sterile filtered (0.22 μm) immediately prior to column application and following pooling of SEC fractions. The EBOV-GP-I53-50 and SUDV-GP-I53-50 were prepared as independent productions on at least three separate occasions. See (Supplementary Figs 11–14 & 24–26) for certificate of testing information on the GP nanoparticle immunogens prepared in November of 2019 with hexa-histidine tagged I53-50B.4PT1 for use in the maEBOV study (Fig. 4). See (Supplementary Figs 24–26) for certificate of testing information on the GP nanoparticle immunogens prepared with tagless I53-50B.4PT1 in July of 2021 for use in the gpaSUDV study (Fig. 5). The third preparations of these GP nanoparticle immunogens occurred in August of 2022 with tagless I53-50B.4PT1 (see Supplementary Fig. 9b and Fig. 3 for nsEM of the EBOV-GP-I53-50 prepared in August of 2022).

### Assembly production and purification of bare I53-50 nanoparticles

Bare I53-50 nanoparticles without any GP fusions were produced using I53-50A and I53-50B.4PT1 with hexa-histidine tag for the maEBOV study (Supplementary Fig. 19) or tagless I53-50B.4PT1 for the gpaSUDV study. Total protein concentration of purified individual nanoparticle components was determined by measuring absorbance at 280 nm using a UV-Vis spectrophotometer (Agilent Cary 8454) and calculated extinction coefficients. The assembly steps were performed at room temperature with addition in the following order: I53-50A trimeric protein, followed by additional buffer (50 mM Tris pH 7.4, 185 mM NaCl, 100 mM L-arginine, 4.5% glycerol, and 0.75% w/v CHAPS) as needed to achieve desired final concentration, and finally I53-50B.4PT1 pentameric component (in 50 mM Tris pH 8, 500 mM NaCl, 0.75% w/v CHAPS), with a molar ratio of I53-50A:I53-50B.4PT1 of 1.1:1. All I53-50 in vitro assemblies were incubated briefly at room temperature before subsequent purification by SEC in order to remove residual unassembled I53-50A components. A Superose 6 Increase 10/300 GL column was used for nanoparticle purification. Assembled nanoparticles were purified in 50 mM Tris pH 7.4, 185 mM NaCl, 100 mM Arginine, 4.5% v/v glycerol, and 0.75% w/v CHAPS, and eluted at ∼11 mL on the Superose 6 column. Assembled nanoparticles were sterile filtered (0.22 μm) immediately prior to column application and following pooling of fractions. The “naked” or “bare” I53-50 nanoparticle samples have been produced and purified on numerous occasions^35,38,39,47,48,50^.

### Endotoxin measurements

Endotoxin levels in protein samples were measured using the EndoSafe Nexgen-MCS System (Charles River). Samples were diluted 1:100 in Endotoxin-free LAL reagent water and applied into wells of an EndoSafe LAL reagent cartridge. Charles River EndoScan-V software was used to analyze endotoxin content, automatically back-calculating for the dilution factor. Endotoxin values were reported as EU/mL which were then converted to EU/mg based on UV-Vis measurements. Our threshold for samples suitable for immunization was <50 EU/mg.

### CryoEM sample preparation, data collection, and data processing for EBOV-GP-foldon

To prepare the samples, 2 μL of EBOV-GP-Foldon produced from our pCMV/R vector by transient transfection and purified by IMAC and SEC at a concentration of 1 mg/mL in 300 mM NaCl, 20 mM Tris, pH 7.5, was applied to 2.0/2.0-T C-flat holey carbon grids, glow discharged for 25 s at 15 mA. Vitrification was performed using a Mark IV Vitrobot at 22 °C with 100% humidity for all. Blotting was done using a 7.0 s blot time, a blot force of 0 and a 7.5 s wait time was used before being immediately plunge frozen into liquid ethane. The cryoEM dataset for EBOV-GP-Foldon was collected automatically using Leginon^103^, used to control a ThermoFisher Titan Krios 300 kV microscope was equipped with a K3 direct electron detector^104^ and BioQuantum Gif energy filter and operated in counting mode. The random defocus range spanned between −0.8 and −1.8 μm. 3128 movies with a pixel size of 0.84, total dose of 56.7 e^-^/Å^2^ were recorded with a total exposure of 5 s over 100 frames.

All data processing was carried out in CryoSPARC^105^. Default parameters were used for refinement unless otherwise noted. The video frames were aligned using Patch Motion Correction, and defocus and astigmatism values were estimated using the Patch CTF Estimation. Curate Exposures was used to remove 1007 poor-quality micrographs, leaving 2121 good micrographs. In total, 1,305,686 particles were picked in a reference-free manner using Blob Picker and further refined with Inspect Picks leaving 585,677 particles. 540,665 particles were extracted with a box size of 222 pixels. An initial round of reference-free two-dimensional (2D) classification was performed using 150 classes. The best 13 classes containing 71,628 particles were further classified with 150 classes. The best 12 classes containing 11,519 particles were low pass filtered to 20 Å and used as templates for a second round of particle picking using Template Picker. 1,579,983 particles were picked and refined to 1,579,983 particles with Inspect Picks. 1,407,670 particles were extracted with an extraction box size of 256 pixels. 2D Classification was used with 200 classes, a maximum resolution of 4 Å, number of online-EM iterations set to 75, with number of final full iterations set to 25, batch size per class of 300, and hard classify for last iteration set to true. The best 16 classes containing 190,512 particles were used to generate an Ab-Initio in C1 using 2 classes. The best class used for Non-Uniform Refinement generated a 3.17 Å volume in C3. The map was then used for a Reference Based Motion Correction job. Finally, a Non-Uniform Refinement was used to generate a: 3.05 Å map in C3 and a 3.35 Å map in C1. Final maps were sharpened with DeepEMHancer^106^. Local resolution estimates were made in CryoSPARC using a fourier shell correlation of 0.143. 3D maps for the two half-maps, the final unsharpened map and the final sharpened map were deposited in the Electron Microscopy Data Bank EMD-48271.

### CryoEM structure building and validation

The model (PDB ID:5JQ3) was used as an initial reference for building the final cryoEM structures. UCSF Chimera^107^ was used to fit the model into density. Multiple rounds of relaxation and minimization were performed on the complete structure, which was manually inspected for errors each time using Isolde^108^, Coot^109,110^, and Phenix^111^ real-space refinement. Final model quality was analysed using MolProbity^112^. Figures were generated using UCSF ChimeraX^113^. The final structure was deposited in the protein data bank (PDB ID: 9DZE). For the superposition to 5JQ3, the RMSD calculations were observed in pyMOL using the Align command, with 3945/5825 atoms used for final alignment after 6 cycles.

### Negative stain transmission electron microscopy

Assembled icosahedral I53-50 GP immunogen samples were thawed and diluted (EBOV-GP-I53-50 sample to 0.05 mg/mL, SUDV-GP-I53-50, EBOV/SUDV-GP-I53-50 – Mosaic, and EBOV-GP-I53-50 + SUDV-GP-I53-50 – cocktail samples to 0.10 mg/mL) and (EBOV-GP-I53-50A and SUDV-GP-I53-50A to 0.01 mg/mL) in 25 mM Tris/HCl pH 7.5 150 mM NaCl buffer (MilliporeSigma) and immediately applied to a freshly glow-discharged 10 nm thick carbon film 400 mesh copper grid (Electron Microscopy Sciences). A volume of 3 µL solution was applied to the grid for 30 s before excess liquid was blotted away (Whatman) and immediately replaced with 3 µL 2% Uranyl Formate stain (STI Supplies), which was subsequently blotted after 10 s. Two additional applications and blotting of 2% Uranyl Formate stain were performed, preceding the sample being air dried for 5 min.

Electron microscopy data were collected for each sample using EPU (ThermoFisher) on a ThermoFisher Talos L120C 120 kV transmission electron microscope with a LaB_6_ filament and a 2.49 Å pixel size with a BM-CETA camera at 57,000 times magnification, and a total dose ~50 e/Å^2^. Micrographs were imported into CryoSPARC v4.4.1 (Structura), and Patch CTF Estimation was performed with a minimum resolution of 40 Å and a maximum resolution of 8 Å. Micrographs for assembled NPs were blob picked with a minimum diameter of 250 Å and a maximum diameter of 350 Å. Particles were then inspected, and those that were accepted were extracted at a box size of 280 pixels (697.2 Å) and binned into 50 2D classes with a batch size of 400 and CTF correction turned off. The best classes for each sample were selected to serve as templates for template picking (with an estimated particle diameter of 280 Å), an additional round of particle inspection, extraction, and an additional round of 2D binning. All jobs in the second round, aside from the picking job, used the same parameters as they did during the first round. Micrographs for GP-I53-50A (EBOV & SUDV) components were blob picked with a minimum diameter of 250 Å and a maximum diameter of 350 Å. Accepted picks were extracted with a box size of 160 pixels and classified into 50 2D class averages for EBOV-GP-I53-50A and 150 2D class averages for SUDV-GP-I53-50A. For all samples, the best classes for each sample were selected for 2D analysis and 3D reconstruction. 3D reconstructions used Ab Initio to generate an initial map and non-uniform refinement with a maximum alignment resolution of 20 Å and 12 Å to generate the final maps, for NP’s and GP-I53-50A components, respectively.

### Mouse immunizations and maEBOV viral challenge

At 10–15 weeks of age, 10 female BALB/c mice (Jackson Laboratories) per dosing group were vaccinated by the subcutaneous (s.c.) route with a prime immunization, and three weeks later mice were boosted with a second vaccination s.c. The subcutaneous route was selected following guidance from IACUC, to avoid potential pain at the injection site that could ensue from an intramuscular route. Prior to inoculation, immunogen suspensions were gently mixed 1:1 (vol/vol) with AddaVax adjuvant (Invivogen, San Diego, CA). Final doses were as follows: Groups 1–4 received 1.9 µg of GP antigen (3.4 µg total protein) in a 100 µL injection volume, corresponding to a final GP antigen concentration of 0.019 mg/mL. Groups 5–7 received 1.6 µg of GP antigen (2.5 µg of GP-I53-50A trimer and 0.8 µg of “2OBX”) in a 100 µL injection volume, corresponding to a final GP antigen concentration of 0.016 mg/mL. Group 8 received 2.5 µg of I53-50 nanoparticles in a 100 µL injection volume, with no GP antigen (0 mg/mL). To obtain sera, all mice were bled two weeks after prime and boost immunizations. Blood was collected by the submandibular or retro-orbital route and rested in 0.8 mL serum separator tube (SST) tube at room temperature for 10 min to allow for coagulation. Serum was separated from red blood cells via centrifugation at 10,000 × *g* for 10 min. Serum was stored at 4 °C or 80 °C until use. For viral challenge, mice were acclimated to ABSL-4 for at least 7 days and were injected by the intraperitoneal (IP) route with a 100 pfu target dose of maEBOV^74^ in a volume of 200 µL. Post-exposure, mice were observed daily for weight and clinical signs of disease for up to 28 days. Upon onset of clinical signs of disease, mice were observed at least twice daily. Moribund mice, with maximum clinical disease scores of 4 (defined as bleeding, unresponsive, weak, unable to walk) or surviving through day 28 post exposure were euthanized. The full scoring/euthanasia criteria was as follows: 0 = normal (smooth coat; eyes/nose clear); 1 = reduced grooming/ruffled fur; 2 = subdued response, but normal when stimulated; 3 = lethargic; hunched posture; subdued response, even when stimulated; 4 = (euthanize) unresponsive when provoked, weak, unable to walk. Throughout the study, animals were housed in cages and rooms were maintained on a 12 h:12 h light-dark cycle (lights on at 0600 h, lights off at 1800 h). Humidity was between 30–70% and temperature kept between 68–76 °F. They were fed ad libitum with a species-appropriate Teklad Global diet (Harlan Teklad), and water ad libitum via an automatic watering system.

### Guinea pig immunizations and gpSUDV viral challenge

Groups of 6 female Hartley guinea pigs (Charles River) per dosing group (except for Group 1 which had 5 animals due to one loss in preparation), weighing 350–500 grams were vaccinated by the subcutaneous (s.c.) route with a prime immunization, and three weeks later guinea pigs were boosted with a second vaccination by the s.c. route. The s.c. route was selected following guidance from IACUC to avoid potential pain at the injection site that could ensue from an intramuscular route. Prior to inoculation, immunogen suspensions were gently mixed 1:1 vol/vol with AddaVax adjuvant (Invitrogen, San Diego, CA). Final doses were as follows: Groups 1–4 received 2.4 µg of GP antigen (4.3 µg total protein) in a 250 µL injection volume, corresponding to a final GP antigen concentration of 0.0096 mg/mL. Group 5 received 2.5 µg of I53-50 nanoparticles in a 250 µL injection volume, with no GP antigen (0 mg/mL). To obtain sera all guinea pigs were bled two weeks after prime and boost immunizations. Blood was collected via submental venous puncture and rested in 0.8 mL SST tube at room temperature for 10 min to allow for coagulation. Serum was separated from red blood cells via centrifugation at 10,000 g for 10 min. Complement factors and pathogens in isolated serum were heat-inactivated via incubation at 56 °C for 60 min. Serum was stored at 4 °C or 80 °C until use. At ~8 weeks post-prime vaccination, animals were moved into ABSL-4 for acclimation. At ABSL-4, guinea pigs were exposed by the IP route with 1000 pfu of gpaSUDV^78^ in a volume of 400 µL. Guinea pig clinical observations, weights, and temperatures (by subcutaneous chip; BDMS) were recorded daily for 28 days. Moribund guinea pigs were promptly euthanized when they met predefined euthanasia criteria, a score of 4; defined as bleeding, unresponsive, weak, or unable to walk. The full scoring/euthanasia criteria was as follows: 0 = normal (smooth coat; eyes/nose clear); 1 = reduced grooming/ruffled fur; 2 = subdued response, but normal when stimulated; 3 = lethargic; hunched posture; subdued response, even when stimulated; 4 = (euthanize) unresponsive when provoked, weak, unable to walk. Throughout the study, animals were housed in cages and rooms were maintained on a 12 h:12 h light-dark cycle (lights on at 0600 h, lights off at 1800 h). Humidity was between 30–70% and temperature kept between 68–76 °F. They were fed ad libitum with a species-appropriate Teklad Global diet (Harlan Teklad), and water ad libitum via an automatic watering system.

### Enzyme-linked immunosorbent assay (ELISA)

Endpoint IgG serum ELISAs to detect EBOV or SUDV antibodies were conducted using the following assay. ELISA plates (Falcon) were coated with recombinant SUDV-GP or recombinant EBOV-GP (NCI, Frederick MD) with 50 µL at 0.1 µg/well in phosphate buffer saline (PBS) (Sigma-Aldrich) and stored at 4 °C overnight. After blocking with PBS/5% Milk (BioWorld) /0.05% Tween 20 (Sigma) (blocking buffer) at room temperature (RT) for 2 h, serum was diluted at 1:100 and then serially in ½ log dilutions with positive and negative control samples, all diluted in blocking buffer, were added to wells in duplicate, 50 µL per well. Following 1 h incubation at RT, plates were washed with PBS/0.05% Tween 20. The secondary antibody, (HRP goat anti mouse IgG KPL: 074-1806 1:2000 dilution, or HRP goat anti guinea pig IgG KPL: 5220-0366 1:1000 dilution) was diluted in the blocking buffer and was added to the plates at RT for 1 h. After washing plates with PBS/0.05% Tween 20, 2,2’-azino-bis(3-ethylbenzothiazoline-6-sulfonic acid (ABTS) (SeraCare) was added for 30 min at RT and absorbance was measured at 405 nm using a SpectraMax® M5 (Molecular Probes). The reciprocal endpoint dilutions were reported.

### SUDV and EBOV GP protein production for ELISA

The EBOV and SUDV GPs (∆TM) used for enzyme-linked immunosorbent assay (ELISA) based serology were produced as follows. *Cloning of Ebola Zaire and Sudan:* Entry clones for GPss-EbGP12-His6 and EbV Sudan-Gulu GP-His6 were created via Gateway methods using PCR-amplified inserts. A carboxy terminal His6 tag was included for purification. Entry clones were transferred to Expression clones by Gateway LR recombination (ThermoFisher Scientific) with pDest-720 (Sabine Geisse, Novartis). Maxiprep DNA was generated for the two constructs using the GenElute HP Plasmid Maxiprep Kit (Sigma). *Transfection of high density HyClone 293E cells at a one-liter volume:* Cells were grown in a Fernbach flask and placed in a 37 °C shaker incubator with 5% CO_2_ at an orbit speed of 100–110 rpm. 24 h prior to transfection, 293E cells were refreshed with an additional 50:50 medium. Cells were counted and enough cells were centrifuged to reach a density of 5 × 106 vc/mL for 500 mLs. Cells were washed with 1x with Gibco FreeStyle Medium (FS). After washing, cells were resuspended in 500 mLs in FS medium in a 2 L Fernbach flask and placed in a 37 °C CO_2_ shaker incubator at an orbit speed of 100–110 rpm. Culture was equilibrated on the shaker before adding DNA/PEI mixture for 10 min. *Preparation of DNA/PEI complexes:* 2 × 2.5 mLs of 5% glucose was added to two 50 mL conicals. 1 mg DNA was added to tube 1 (2 µg DNA per mL of cell culture). PEI is made at 1 mg/mL and the ratio of PEI:DNA is determined with each lot. The calculated volume of PEI was added to Tube 2. The PEI tube contents were added to the DNA tube contents and vortexed for 10 s. The sample was incubated for 5 min, then added to the culture. The culture was incubated for 4 h at 37 °C in a shaker incubator with 5% CO_2_ at an orbit speed of 100–110 rpm. *Post-transfection:* After 4 h, 500 mLs of HyClone HEK293 medium was added to the Fernbach. Culture was harvested 72 h post-transfection by centrifuging at 2375 × *g*/13 min to collect supernatant and remove cells. *Tangential Flow Filtration:* Supernatant was collected and filtered using a Millipak 60, 0.45/0.2 µm or similar filter). The filtrate was concentrated to 500 mL using two 0.1 m^2^ Hydrosart (cellulose), 30 kDa cutoff ultrafiltration slice cassettes in a Sartocon Slice stainless steel holder (Sartorius). To maintain appropriate sample flow, PharMed BPT tubing (Masterflex, size 73) was used in a high-flow peristaltic pump. After concentrating, the protein solution was buffer exchanged with 2.5 liters of 20 mM HEPES and 200 mM NaCl. *Protein Purification:* A 50 mL IMAC column (GE Healthcare Ni Sepharose 6 FastFlow) was equilibrated with 20 mM HEPES pH 7.3, 200 mM NaCl, 35 mM imidazole (equilibration buffer, EB). Buffer-exchanged sample was amended with 35 mM imidazole and applied to the equilibrated IMAC column. A flow rate was chosen to allow a residence time of 35–100 min. The column was washed to baseline with EB and then washed with 10 column volumes (CV) of low salt buffer (EB but with 75 mM NaCl). A 10 mL Q sepharose column is added downstream of the IMAC column. The Q column was equilibrated in EB buffer at 75 mM NaCl prior to downstream addition. Proteins are eluted from the IMAC column (and subsequently captured by the downstream Q sepharose column using 20 mM HEPES, pH 7.3, 75 mM NaCl, 400 mM imidazole buffer (2 IMAC column CVs). The Q column is washed with 20 mM HEPES, pH 7.3, 75 mM NaCl until a steady baseline is reached, and proteins are eluted from the Q column with a 20 CV gradient from 75 mM to 1.0 M NaCl in 20 mM HEPES, pH 7.3 (no imidazole). All steps are monitored by SDS-PAGE/Coomassie-staining, and pools are created based on this assessment of elution fraction purity. The final protein sample was dialyzed to 1x PBS, pH 7.2 and the protein concentration determined by Bradford assay with Bovine Serum Albumin (BSA) as standard (BioRad). Final pooled protein was concentrated 10 K MWCO Millipore Ultra concentrator units to between 0.5 and 1.0 mg/mL and aliquoted in 0.5 mg aliquots.

### Thermal stability assay by intrinsic tryptophan fluorescence

Measurement of intrinsic tryptophan fluorescence is a biophysical technique used to approximate the thermal stability of proteins by measuring intrinsic fluorescence from tryptophan residues which change as the protein unfolds under a controlled thermal ramping. As the temperature increases, the protein begins to unfold, exposing more tryptophan residues of the protein to solvent causing a fluorescence emission shift that can be correlated to the protein melting temperature (Tm, where ~50% of the sample has experienced the change in tryptophan fluorescence). Measurements of intrinsic tryptophan fluorescence were conducted on protein samples at 0.10 mg/mL using an UNcle (nanoDSF instrument from UNchained Labs). Intrinsic tryptophan fluorescence was monitored from 25 °C to 95 °C with an increasing ramp rate of 0.15 degrees C/minute and an acquisition time of 10 s to measure emission shifts or intensity changes as a function of temperature. Values reported are the mean value across 10 acquisitions. Instrument software was used to calculate the Tm values, including for samples that had two main temperature shift induced changes in fluorescence, noted as Tm_1_ and Tm_2_.

### Statistics and reproducibility

Statistical details of experiments can be found in the figure legends. For animal samples analysis, no blinding of the experimenter was done. For animal studies, 10 BALB/c or 6 guinea pigs were used. No statistical methods were used to predetermine sample size. For animal samples analysis, no blinding of the experimenter was done. Geometric mean anti-GP IgG titers were calculated based on absolute ELISA signals with background removed. For the ELISA plot data in the maEBOV study, we ran statistical tests and calculated significance levels by comparison of all individual test article Groups to the bare I53-50 nanoparticle group. We first conducted normality testing using the Shapiro-Wilk test to assess normality for both the current Group and the reference Group 8. If both groups are normally distributed (*p* > alpha), we perform an independent t-test. If not, we use the Mann–Whitney U test as a non-parametric alternative. We assume normality since Shapiro–Wilk requires at least 3 observations, and all groups had more than 3 data points. We determined the significance level based on the *P* value. The level of statistical significance is indicated as: *P < 0.05; **P < 0.01; ***P < 0.001. Kruskal–Wallis survival tests were performed for survival data, and calculated *P* values for statistical significance are indicated for comparisons against the respective naked I53-50 NP control group based on log-rank Mantel-Cox statistical analyses. The level of statistical significance is indicated as: *P < 0.05; **P < 0.01; ***P < 0.001.

The EBOV GP construct was produced in a single expression preparation and purified in two separate batches. Each purification underwent IMAC, SEC, and SDS-PAGE analysis. CryoEM analysis was performed once using material from the first round of purification. Production of all other EBOV and SUDV GP constructs reported was produced at least once using lentivirus production and at least twice using pCMV/R transient transfection. IMAC and SEC purification and analytical HPLC SEC were performed for all constructs and nanoparticle preparations at least once for in vitro characterization and multiple times prior to animal studies. Representative SEC profiles and SDS-PAGE analyses for various constructs were selected for comparison. Production and certificate of testing for all GP-I53-50 nanoparticle immunogens was completed on at least two separate occasions for the mouse and guinea pig studies. Negative-stain EM was performed routinely for all GP-I53-50 nanoparticle assemblies during in-vitro characterization and production of immunogens for animal studies. Full Negative-stain EM analysis including (collecting micrographs ~200 each), 2D class averages, 3D reconstructions was done once for: EBOV and SUDV I53-50a components, both for lentivirus and pCMV/R transient transfection produced material; and once for each nanoparticle formulation. SDS-PAGE analysis was conducted at least once for all constructs produced. All ELISA binding assays were performed in duplicate from fresh aliquots of single serum lot for each animal. Due to the limited availability of purified mouse or guinea pig IgG samples, in vitro viral neutralization assays were not performed.

### Reporting summary

Further information on research design is available in the Nature Portfolio Reporting Summary linked to this article.

## Supplementary information

Supplemental Information

Description of Additional Supplementary File

Supplemental Data 1

Reporting Summary

Transparent Peer Review file

## Source data

Source Data for All Figures

## Data Availability

The datasets generated during and/or analyzed during the current study are available in the source data document. The atomic coordinates and map for our cryoEM structure of the prefusion EBOV-GP-Foldon construct is located at the Protein Data Bank and Electron Microscopy Data Bank under accession codes: (PDB ID: 9MHA EMDB ID: EMD-48271). Source data are provided with this paper.

## Disclaimers

The opinions, interpretations, conclusions, and recommendations presented are those of the authors and are not necessarily endorsed by the U.S. Army or Department of Defense. The funders had no role in the design of the study; in the collection, analyses, or interpretation of data; in the writing of the manuscript; or in the decision to publish the results. The use of trade or manufacturers’ names in this publication does not constitute an official endorsement of any commercial products. This report may not be cited for the purposes of advertisement. Contractor affiliation of some authors does not imply endorsement by the U.S. Government of any contractor.
