# Supplementary material for: Pan-Ebolavirus nanoparticle vaccine provides protection in rodents from lethal infection by Zaire and Sudan viruses
Source: Nat Commun. 2026 Aug 8;17:9541. doi: 10.1038/s41467-026-76114-1 (PMC13547272; doi:10.1038/s41467-026-76114-1)
Supplement: Supplementary file 1 [file 41467_2026_76114_MOESM1_ESM.pdf]

Supplemental Figures and Information

Pan-Ebolavirus Nanoparticle Vaccine Provides Protection in Rodents from Lethal Infection by Zaire and Sudan Viruses

Connor Weidle[1,2,*], Natalie Brunette[1,2,3,*], Samuel P Wrenn[1,2,4], Brooke Fiala[1,2], Rashmi Ravichandran[1,2], Kenneth D Carr[1,2], Samantha E Zak[5], Elizabeth E Zumbrun[5], Russell R Bakken[5], Michael Murphy[1,2,], Zhitong Peng[1,2], Sidney Chan[1,2,3], Rebecca Skotheim[1,2], Lauren Carter[1,2,6], Colin E Correnti[1,2,7], John M Dye[5], David Baker[1,2,8], Neil P King[1,2], Andrew J Borst[1,2†] and Lance J Stewart[1,2,†]

[1]Department of Biochemistry, University of Washington, Seattle, WA 98195, USA

[2]Institute for Protein Design, University of Washington, Seattle, WA 98195, USA

[3]Present address: Archon Biosciences, Seattle, WA 98102, USA

[4]Present address: Bonum Therapeutics, Seattle, WA 98102, USA

[5]United States Army Medical Research Institute of Infectious Diseases (USAMRIID), Fort Detrick, MD, USA

[6]Present address: Gates MRI, Seattle, WA 98102, USA

[7]Clinical Research Division, Fred Hutchinson Cancer Research Center Seattle, WA 98109, USA

[8]Howard Hughes Medical Institute, University of Washington, Seattle, WA, USA

```
AAB81004.1 MGVTGILQLPRDRFKRTSFFLWVIILFQRTFSIPLGVIHNSTLQVSDVDKLVCRDKLSSTNQLRSVGLNLEGNGVA 76
AGL73446.1 MGGLSLLQLPRDKFRKSSFFVWVIILFQKAFSMPLGVVTNSTLEVTEIDQLVCKDHLASTDQLKSVGLNLEGSGVS 76

AAB81004.1 TDVPSATKRWGFRSGVPPKVVNYEAGEWAENCYNLEIKKPDGSECLPAAPDGIRGFPRCRYVHKVSGTGPCAGDFA 152
AGL73446.1 TDIPSATKRWGFRSGVPPKVVSYEAGEWAENCYNLEIKKPDGSECLPPPPDGVRGFPRCRYVHKAQGTGPCPGDYA 152

AAB81004.1 FHKEGAFFLYDRLASTVIYRGTTFAEGVVAFLILPQAKKDFFSSHPLREPVNATEDPSSGYYSTTIRYQATGFGTN 228
AGL73446.1 FHKDGAFFLYDRLASTVIYRGVNFAEGVIAFLILAKPKETFLQSPPIREAVNYTENTSSYYATSYLEYEIENFGAQ 228

AAB81004.1 ETEYLFEVDNLTYVQLESRFTPQFLLQLNETIYTSGKRSNTTGKLIWKVNPEIDTTIGEWAFWETKKNLTRKIRSE 304
AGL73446.1 HSTTLFKIDNNTFVRLDRPHTPQFLFQLNDTIHLHQQLSNTTGRLIWTLDANINADIGEWAFWENKKNLSEQLRGE 304

AAB81004.1 ELSFTVVSNGAKNISGQSPARTSSDPGTNTTTEDHKIMASENSSAMVQVHSQGREAAVSHL----------TTLAT 370
AGL73446.1 ELSFEALSLNETEDDDAASSRITKGRISDRATRQYSDLVPKNPPGMVPLHIPEGETTLPSQNSTEGRRVSVNTQET 380

AAB81004.1 ISTSPQSLTTKPGPDNSTHNTPVYKLDISEATQVEQHHRRTDNDSTASDTPSATTAAGP-PKAENTNT-SKSTDFL 444
AGL73446.1 ITETAATII---GTNGNH--MQISTIGIR-----------P----SSSQIPSSSPTTAPSPEAQTPTTHTSGPSVM 436

AAB81004.1 D-------PATTTSPQNHSETAG-NNNTHHQDTGEESASSGKLGLITNTIAGVAGLITGGRRTRREAIVNAQPKCN 512
AGL73446.1 ATEEPTTPPGSSPGPTTEAPTLTTPENITTAVKTVLPQESTSNGLITSTVTGILGSLGLRKRSRRQTNTKATGKCN 512

AAB81004.1 PNLHYWTTQDEGAAIGLAWIPYFGPAAEGIYIEGLMHNQDGLICGLRQLANETTQALQLFLRATTELRTFSILNRK 588
AGL73446.1 PNLHYWTAQEQHNAAGIAWIPYFGPGAEGIYTEGLMHNQNALVCGLRQLANETTQALQLFLRATTELRTYTILNRK 588

AAB81004.1 AIDFLLQRWGGTCHILGPDCCIEPHDWTKNITDKIDQIIHDFVDKTLPDQGDNDNWWTGWRQWIPAGIGVTGVIIA 664
AGL73446.1 AIDFLLRRWGGTCRILGPDCCIEPHDWTKNITDKINQIIHDFIDNPLPNQDNDDNWWTGWRQWIPAGIGITGIIIA 664

AAB81004.1 VIALFCICKFVF 676
AGL73446.1 IIALLCVCKLLC 676
```

Supplemental Figure 1. Comparative Amino Acid Sequence for EBOV and SUDV Ebolaviral Glycoproteins. The amino acid sequences of the primary Ebolaviral glycoprotein sequences were aligned with UniProt[116] for EBOV GP, Zaire Mayinga AAB81004.1 and SUDV GP, Sudan Gulu AGL73446.1, with sequence identities (blue), gaps (-) and non-identity (grey). There is 55% amino acid sequence identity between the EBOV and SUDV GPs.

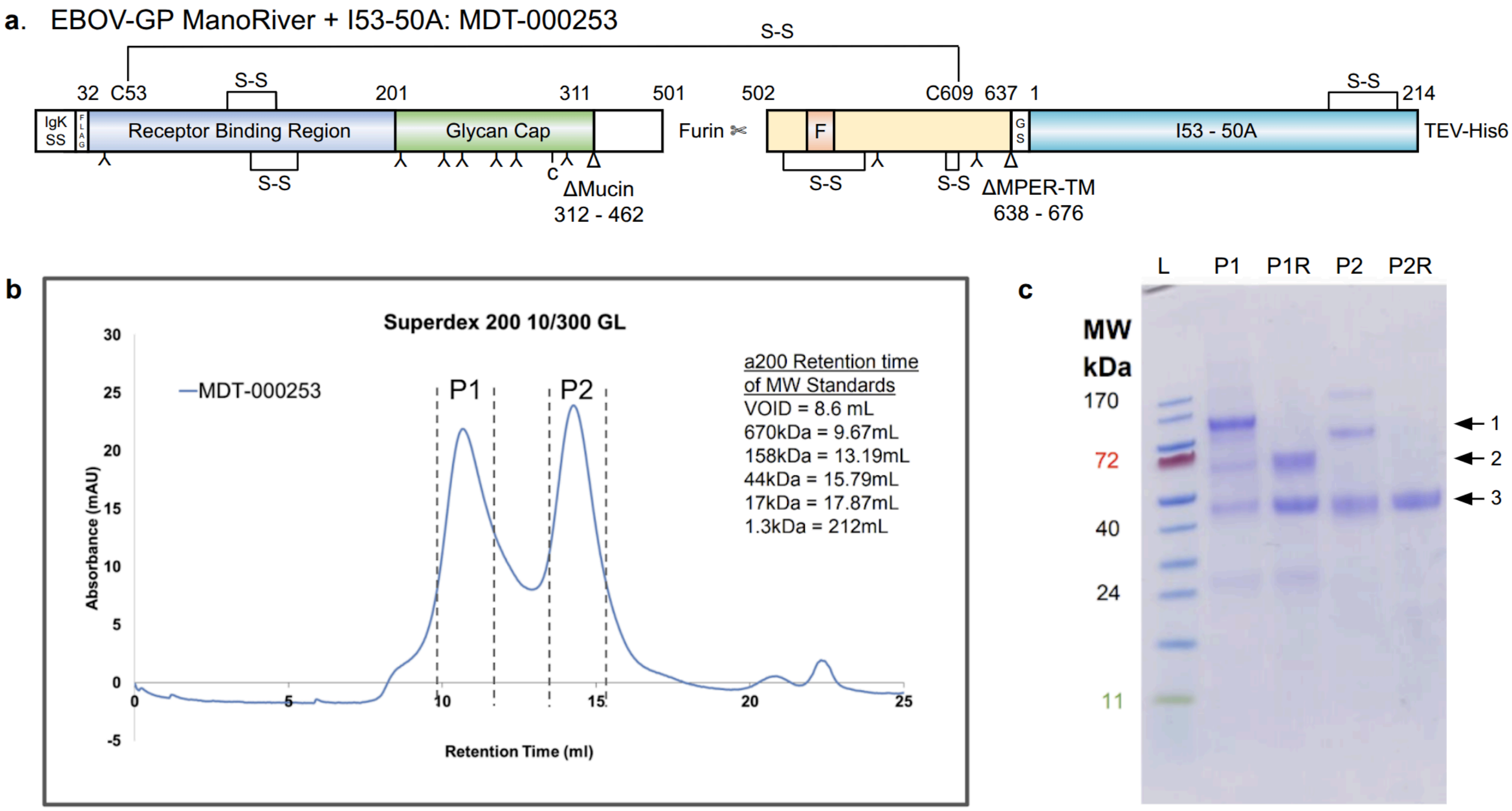

Supplemental Figure 2. Mixed Trimeric and Monomeric Molecular Species for the EBOV-GP Mano River (Δmuc, ΔMPER-TM)-I53-50A-Tev-His6 Secreted from HEK293 by Lentiviral Transduction. Genetic fusion of I53-50A to the C-terminus of Mano River 2014 GP[117] with Δmuc and ΔMPER-TM deletions (MDT-000253, based GenBank AIG96625.1). Secreted HEK293 expression of this construct called "EBOV-GP Mano River (Δmuc, ΔMPER-TM)-I53-50A-Tev-His6" was achieved using a HEK293 lentiviral transduction expression system[66]. However, following purification by IMAC and size-exclusion chromatography (SEC), the secreted protein was produced as a mixture of two different sizes (P1 and P2); likely a mixture of homo-trimer, with dimer and monomer forms as well as forms that lack a disulfide bond between GP1 and GP2. As such, this initial EBOV construct was not suitable for use in the production of self-assembled I53-50 nanoparticles, where there is a strict requirement for a trimeric C3 structure in the I53-50A component which is mixed with purified pentameric I53-50B protein to form icosahedral nanoparticles[40]. This result was not unexpected, since it had previously been reported that secreted expression of EBOV GP ectodomains missing the mucin domain (Δmuc) and transmembrane anchor domain (ΔTM) will form a mixture of molecular species (see Figure S8 in[64] with masses of ~75 kDa (monomers), ~150 kDa (dimers) and ~225 kDa (trimers)). It has also been reported that the Mano River strain sequence contains the A82V mutation known to reduce GP stability[61]. **a.** MDT-000253 protein architecture. The GP of the Zaire-ManoRiver 2014 strain GenBank AIG96625.1[117], missing the mucin domain (Δmuc) and the MPER-TM domain (ΔMPER-TM) is fused to the I53-50A component (light blue) via a GS linker. **b.** The SEC trace (Superdex 200 Increase 10/300 GL) following IMAC purification **c.** The two primary SEC peaks (P1 and P2) were analyzed by SDS-PAGE (4-20% Tris-Glycine gel, 300V for ~20 minutes stained with Coomassie blue) under non-reducing and reducing (R) conditions. Molecular weight (MW) markers in ladder (L) are shown in lane 1 in kilodaltons (kDa). Arrows indicate expected mass locations for band 1. GP1-(ΔMucin)-GP2-(ΔMPER-TM)-I53-50A (lane 2 & 3); Band 2. GP1-(ΔMucin)(lane 3); Band 3. GP2-(ΔMPER-TM)-I53-50A (lanes 2-4).

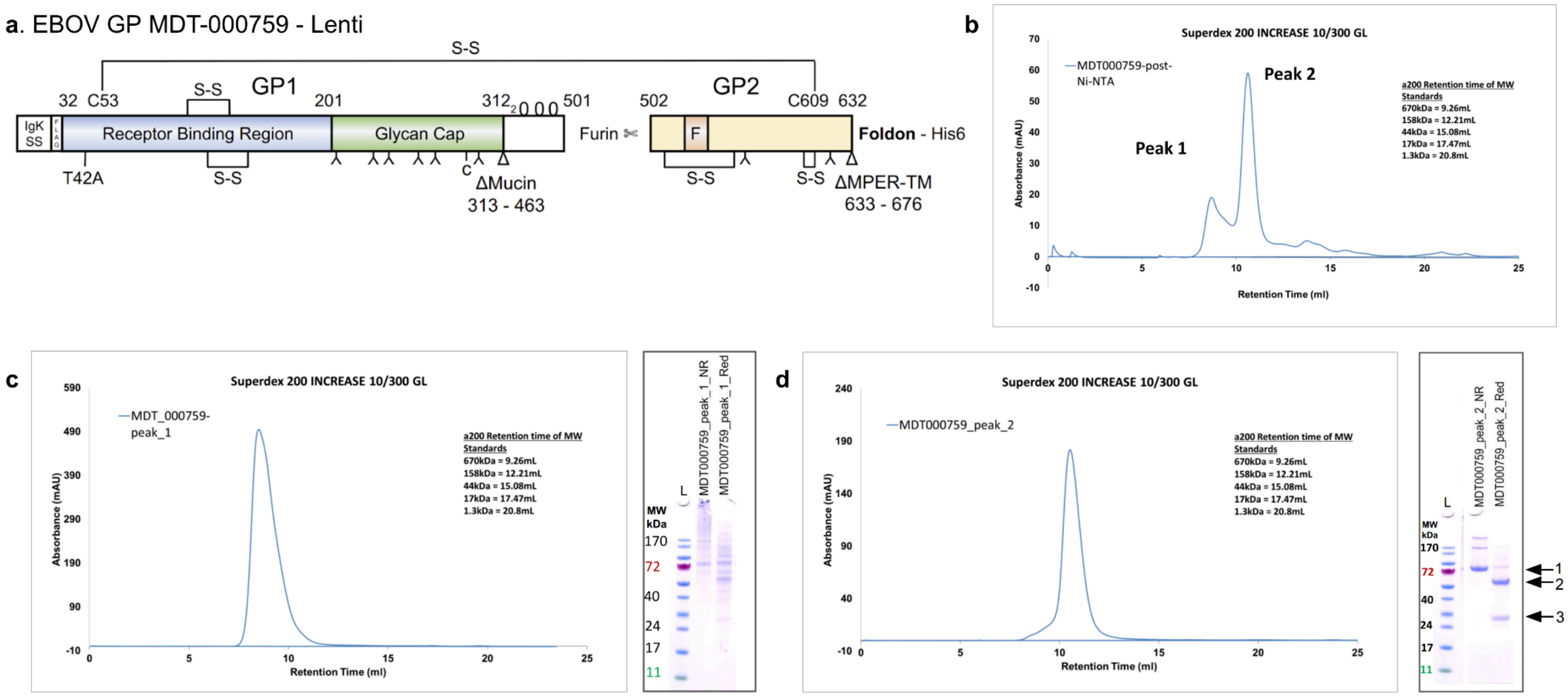

Supplemental Figure 3. Production of EBOV-GP-Foldon in HEK293 by Lentiviral Transduction. a. Protein architecture of EBOV-GP-Foldon, MDT-000759 **b.** This construct was expressed as a soluble secreted protein from HEK293 cells using lentiviral transduction[66], and the secreted protein was purified from culture medium by IMAC and SEC (Superdex 200 Increase 10/300 GL), yielding a leading (Peak 1) fraction along with trailing primary secondary (Peak 2) fraction **c.** SEC Peak 1 was purified again by SEC and analyzed by SDS-PAGE (4-20% Tris-Glycine gel, 300V for ~20 minutes stained with Coomassie blue) under non-reducing (NR) and reducing (Red) conditions. Based on the band pattern of the Peak 1 fraction, it primarily contains aggregates of cell proteins with only a small amount of desired homotrimeric GP. **d.** SEC Peak 2 was purified again by SEC and analyzed by SDS-PAGE (4-20% Tris-Glycine gel, 300V for ~20 minutes stained with Coomassie blue) under non-reducing (NR) and reducing (Red) conditions. Molecular weight (MW) markers are shown in the first lanes in kilodaltons (kDa). Based on the SDS-PAGE band pattern of the Peak 2 fraction, it is the fraction that primarily contains the homotrimeric construct of interest. Full sequence details are provided in Supplemental Data 1. Arrows indicate expected mass locations for band 1. GP1-(ΔMucin)-GP2-(ΔMPER-TM)-I53-50A (lane 2); Band 2. GP1-(ΔMucin) (lane 3); Band 3. GP2-(ΔMPER-TM)-I53-50A (lane 3).

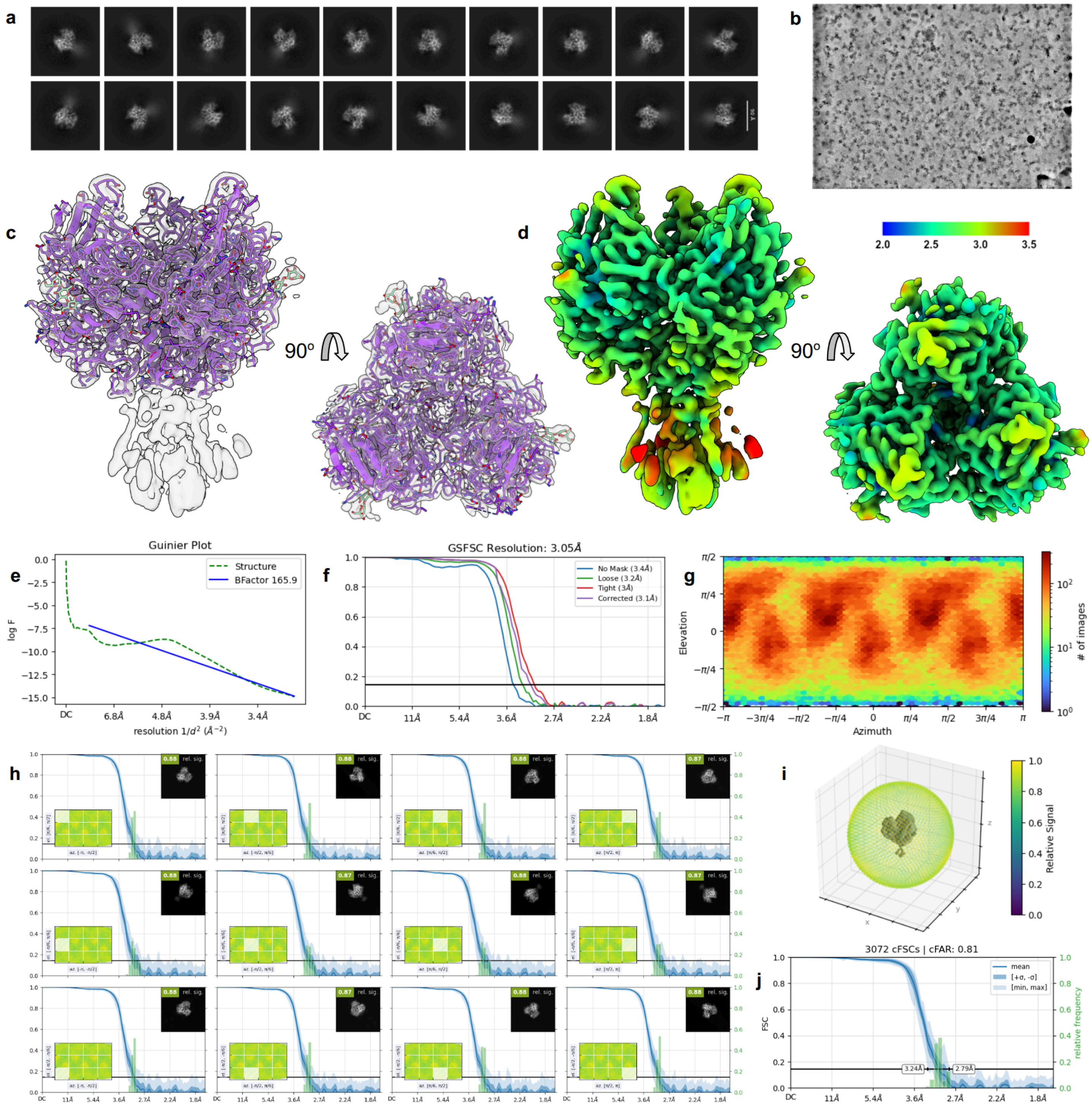

Supplemental Figure 4. CryoEM structure of EBOV-GP: **MDT-000759, Non-Uniform Refinement in C3. a.** 2D class averages **b.** Denoised micrograph, cryoSPARC Micrograph Denoiser **c.** Refined 3.05 Å structure fit into density **d.** Local resolution map Å **e.** Guinier Plot **f.** Global Gold Standard Fourier Shell Correlation (GSFSC) **g.** Orientational distribution plot demonstrating full angular sampling **h.** Orientational diagnostics data **i.** Relative Signal Amount vs. Viewing Direction **j.** Summary of 3072 Conical Fourier Shell Correlations. (PDB ID: 9MHA, EMDB ID: EMD-48271).

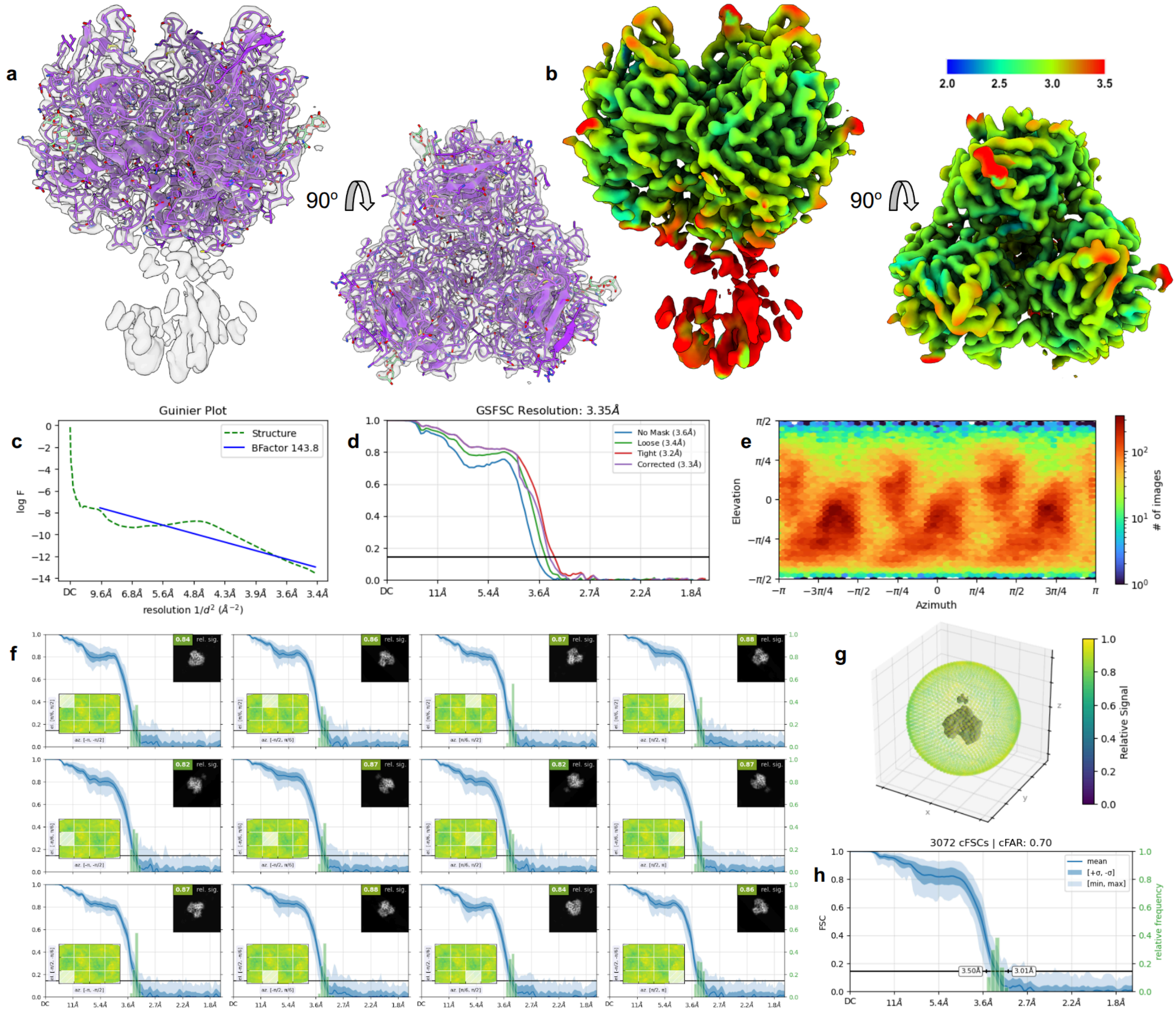

Supplemental Figure 5. CryoEM structure of EBOV-GP: **MDT-000759, Non-Uniform Refinement in C1. a.** 3.35 Å C1 map, with the C3 structure docked into the C1 map **b.** Local resolution map Å **c.** Guinier Plot **d.** Global Gold Standard Fourier Shell Correlation (GSFSC) **e.** Orientational distribution plot demonstrating full angular sampling **f.** Orientational diagnostics data **g.** Relative Signal Amount vs. Viewing Direction **h.** Summary of 3072 Conical Fourier Shell Correlations. (PDB ID: 9MHA, EMDB ID: EMD-48271).

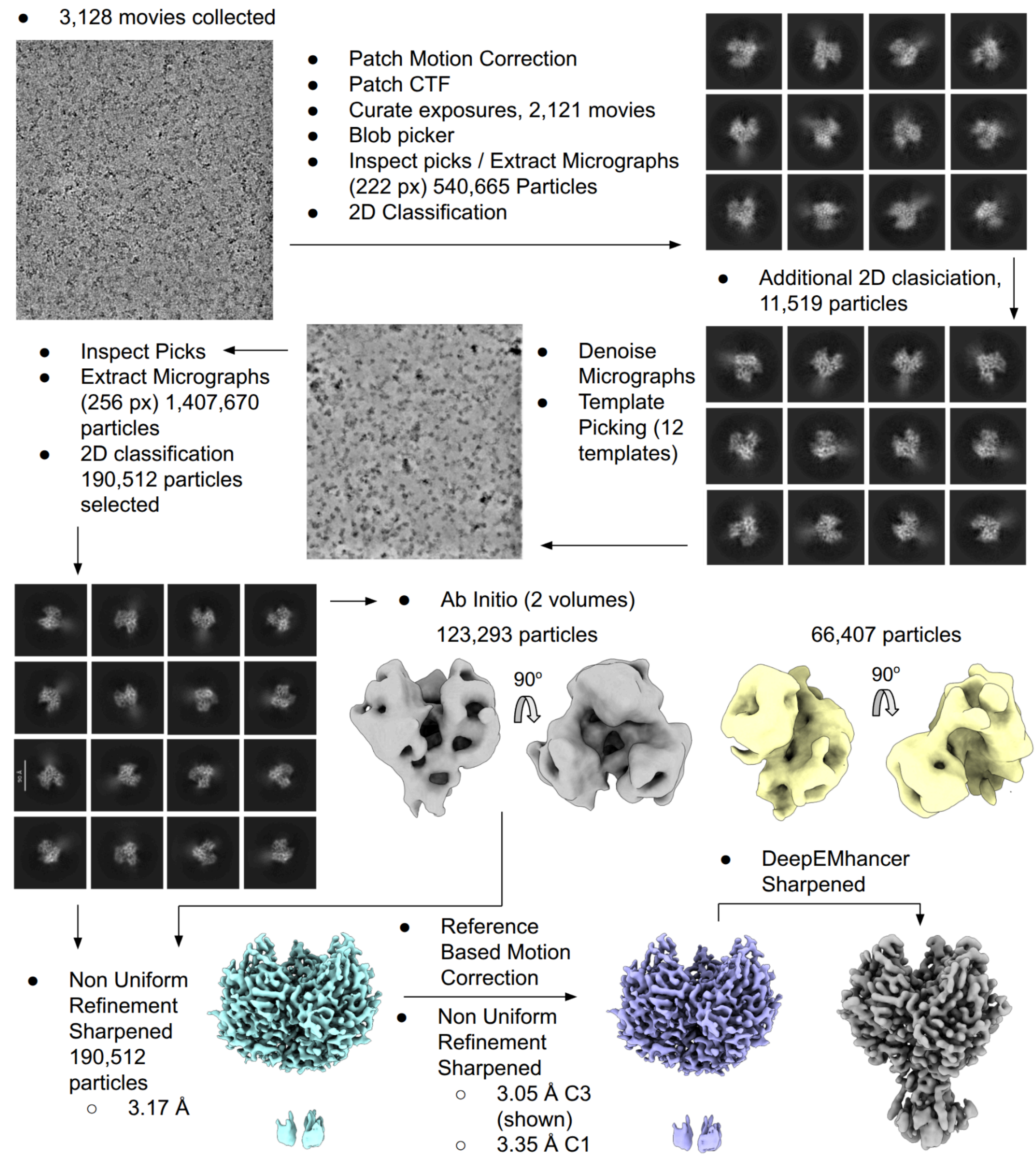

Supplemental Figure 6. CryoEM Data Processing Workflow. All 2D and 3D volumes shown

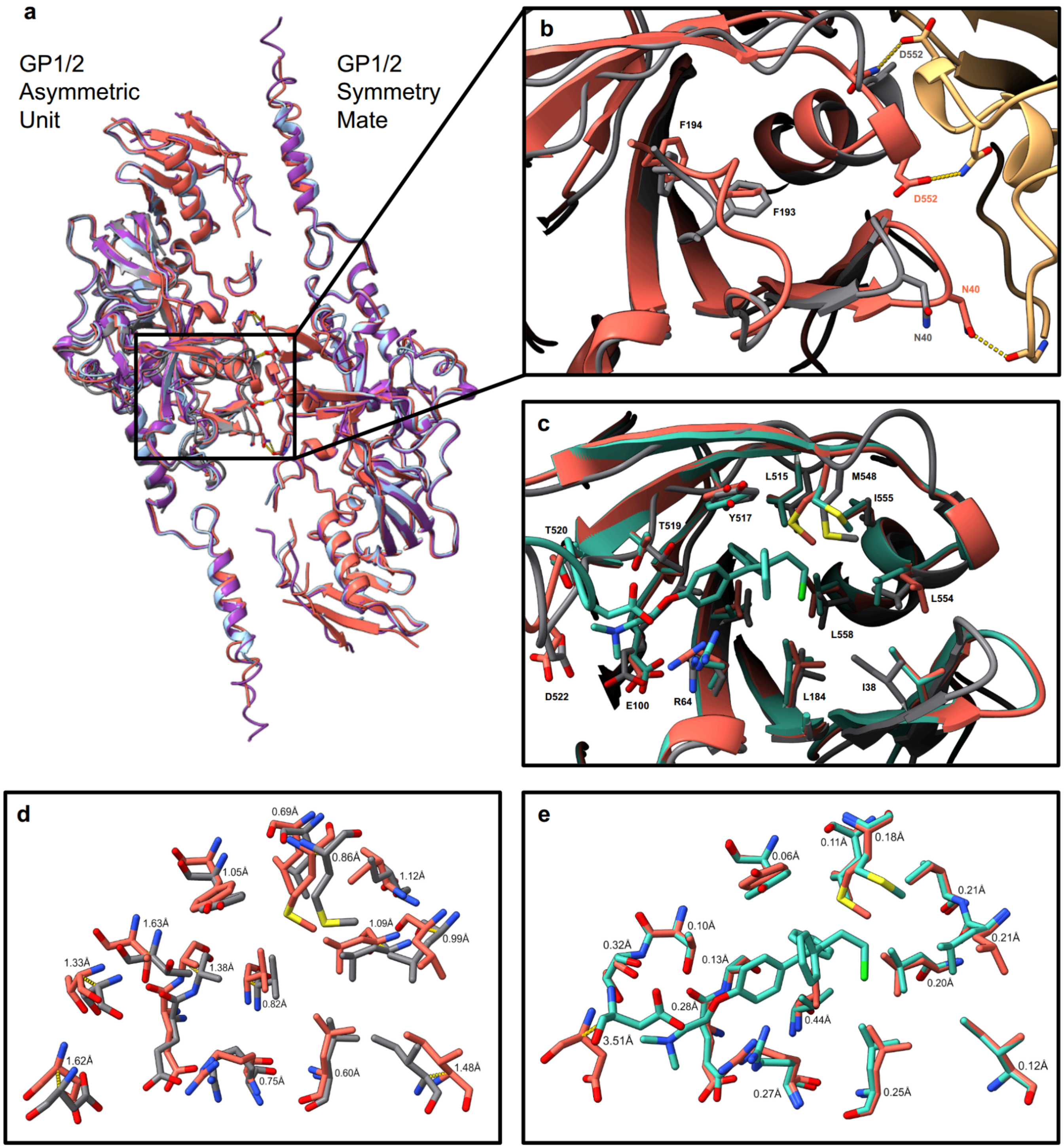

Supplemental Figure 7. Comparison of Apo, Crystal and cryoEM structures and ligand-soaked crystal structure, with a focus on the GP inhibitor binding site. a. The four Ebola GP apo structures (all are trimeric, only ASU shown). Crystal structures share a common crystal packing interface, ASU and symmetry mate shown: Purple: 7JPH, Salmon: 5JQ3, Blue: 7JPI. CryoEM ASU in Gray (PDB ID: 9MHA, EMDB ID: EMD-48271). **b.** Apo crystal structure (Salmon: 5JQ3) aligned with our cryoEM structure in Gray (PDB ID: 9MHA, EMDB ID: EMD-48271) focus on the inhibitor binding site and crystal packing interface, where the tan and salmon chains of 5JQ3 are from neighboring ASUs. In all four apo structures Phe193 & Phe194 occupy the inhibitor binding site. The inhibitor binding site is influenced by crystal packing, notably hydrogen bonds at Asp552 and Asn40 to the symmetry mate in tan. The portions of the cryoEM structure containing these residues adopt a different conformation than that observed in other apo crystal forms. **c.** Alignment of the cryoEM structure (gray), Apo crystal structure with Phe193 & Phe194 not shown for clarity (Salmon: 5JQ3), and soaked crystal structure with Toremifene bound (Teal: 5JQ7; Phe193 & Phe194 are displaced when the inhibitor is soaked in. **d.** Apo cryoEM (gray: 9MHA) and apo crystal structure (Salmon: 5JQ3) compared. Inhibitor binding site residues highlighted with C-alpha carbon distance measured. **e.** Crystal structures, Toremifene soaked (Teal: 5JQ7) vs apo (Salmon: 5JQ3) with C-alpha carbon distance measured.

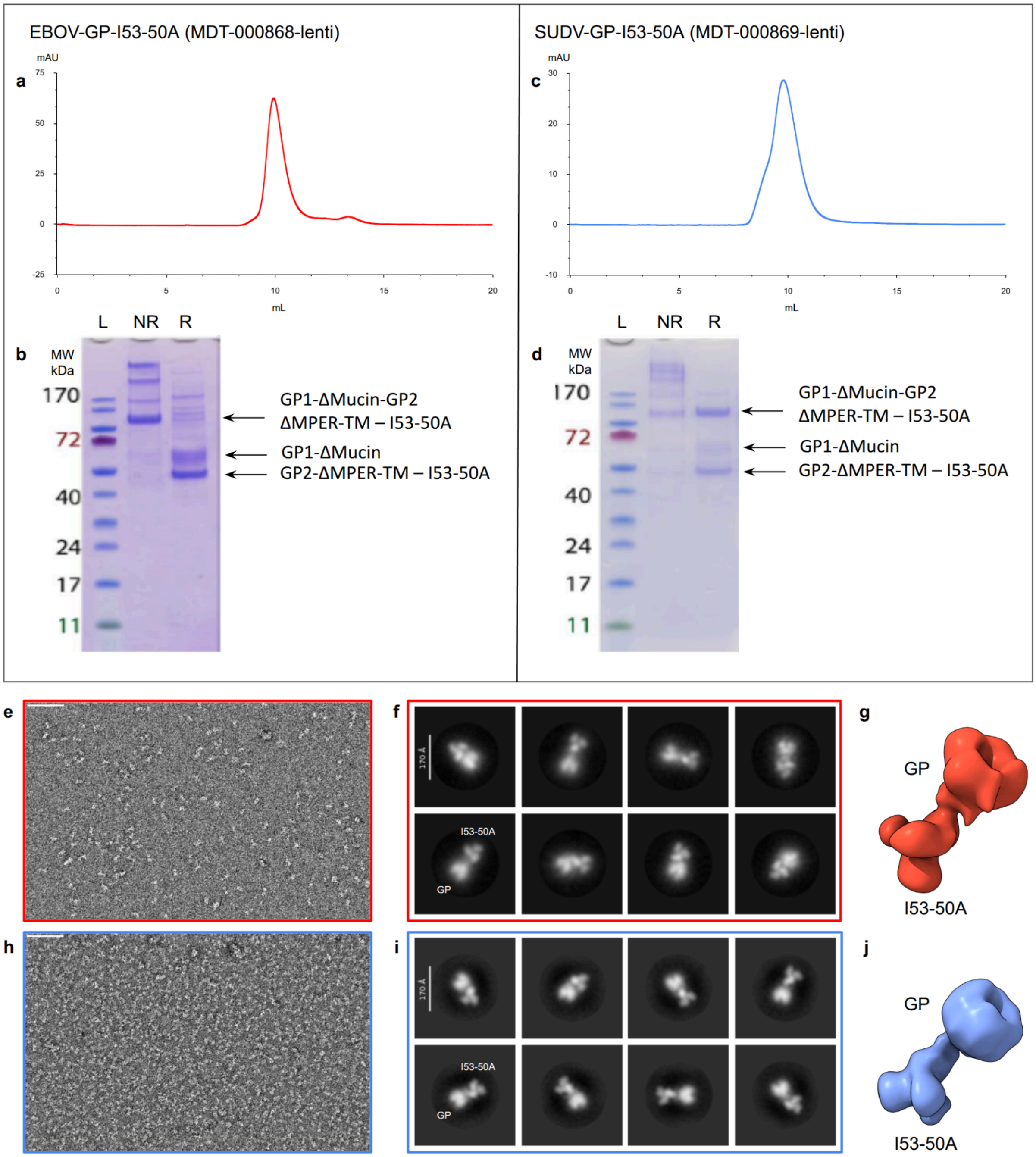

Supplemental Figure 8. EBOV-GP-I53-50A (MDT-000868-lenti) and SUDV-GP-I53-50A (MDT-000869-lenti) expressed in HEK293F cells by Lentiviral Transduction. Constructs were expressed as soluble secreted proteins from HEK293 cells using lentiviral transduction[66], and the secreted protein was purified from culture medium by IMAC and SEC (Superdex 200 Increase 10/300 GL) and then characterized by negative stain electron microscopy (nsEM), and SDS-PAGE gels (4-20% Tris-Glycine gel, 300V for ~20 minutes stained with coomassie blue) under non-reducing (NR) and reducing (R) conditions, molecular weight (MW) markers in kilodaltons (kDa) for ladder (L) shown in the first lanes (see Methods). EBOV-GP-I53-50A (MDT-000868-lenti) was produced in 2 liters of ~$3.7x10^6$ HEK293F cells/mL and produced a total of 7.8 mg of pure protein following IMAC and SEC purification for a total yield of ~4 mg/L of cell culture. SUDV-GP-I53-50A (MDT-000869-lenti) was produced in 2 liter of ~$3.7x10^6$ HEK293F cells/mL and produced a total of 13.4 mg of pure protein following IMAC and SEC purification for a total yield of ~6.7 mg/L of cell culture **a,b.** EBOV-GP-I53-50A (MDT-000868-lenti) **a.** SEC trace **b.** SDS-Page gel **c,d.** SUDV-GP-I53-50A (MDT-000869-lenti) **c.** SEC trace **d.** SDS-Page gel. **e-g.** nsEM analysis of EBOV-GP-I53-50A (MDT-000868-lenti) **e.** nsEM micrograph **f.** 2D class averages **g.** 3D reconstruction, in C1 no symmetry applied **h-j.** (nsEM) analysis of SUDV-GP-I53-50A (MDT-000869-lenti) **e.** nsEM micrograph **f.** 2D class averages **g.** 3D reconstruction, in C1 no symmetry applied.

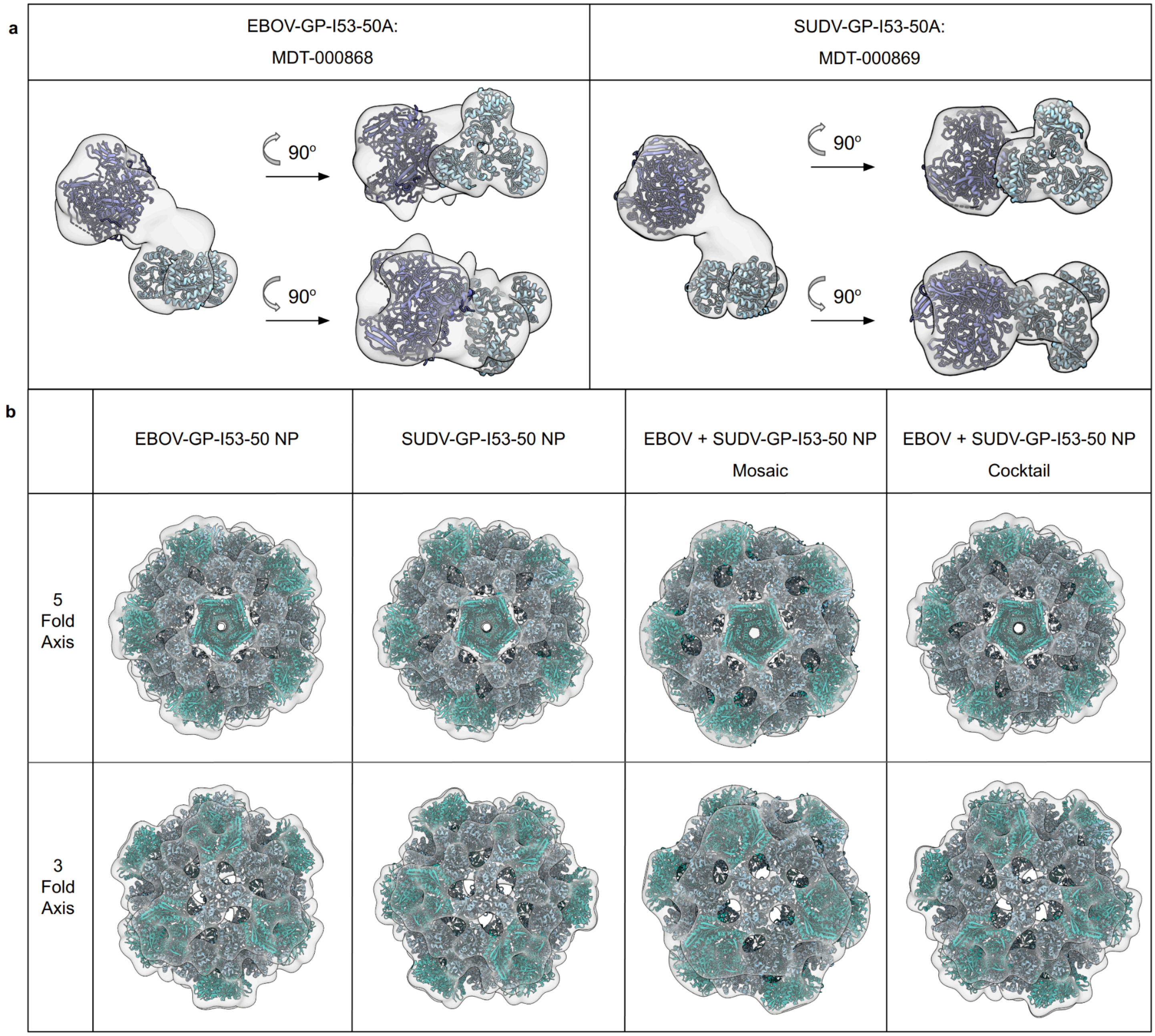

Supplemental Figure 9. Negative Stain 3D Refinement for Figures 2 and 3 with Protein Data Bank (PDB) Models Fit to Density. **a.** GP-I53-50A components fit to negative stain semi-transparent maps from (Supplemental Figure 8) using EBOV-GP-I53-50A and SUDV-GP-I53-50A samples prepared by lentiviral transduction and subsequent IMAC-SEC purification. **b.** GP-I53-50A formed nanoparticle immunogens from (Figure 3) using a preparation of EBOV-GP-I53-50 prepared in August of 2022, and the other NPs prepared in July 2021 for the gpaSUDV study Groups 2-4 (Supplemental Figures 24-26). GP trimer is flexibility attached (GS linker) to the underlying I53-50A component, so it is averaged out of the maps, I53-50 fit to semi-transparent negative stain maps generated from nsEM data from (Figure 2). Models used were (PDB ID: 7SGE)[118] for full nanoparticle & I53-50A component, and our GP cryoEM structure in (Figure 1).

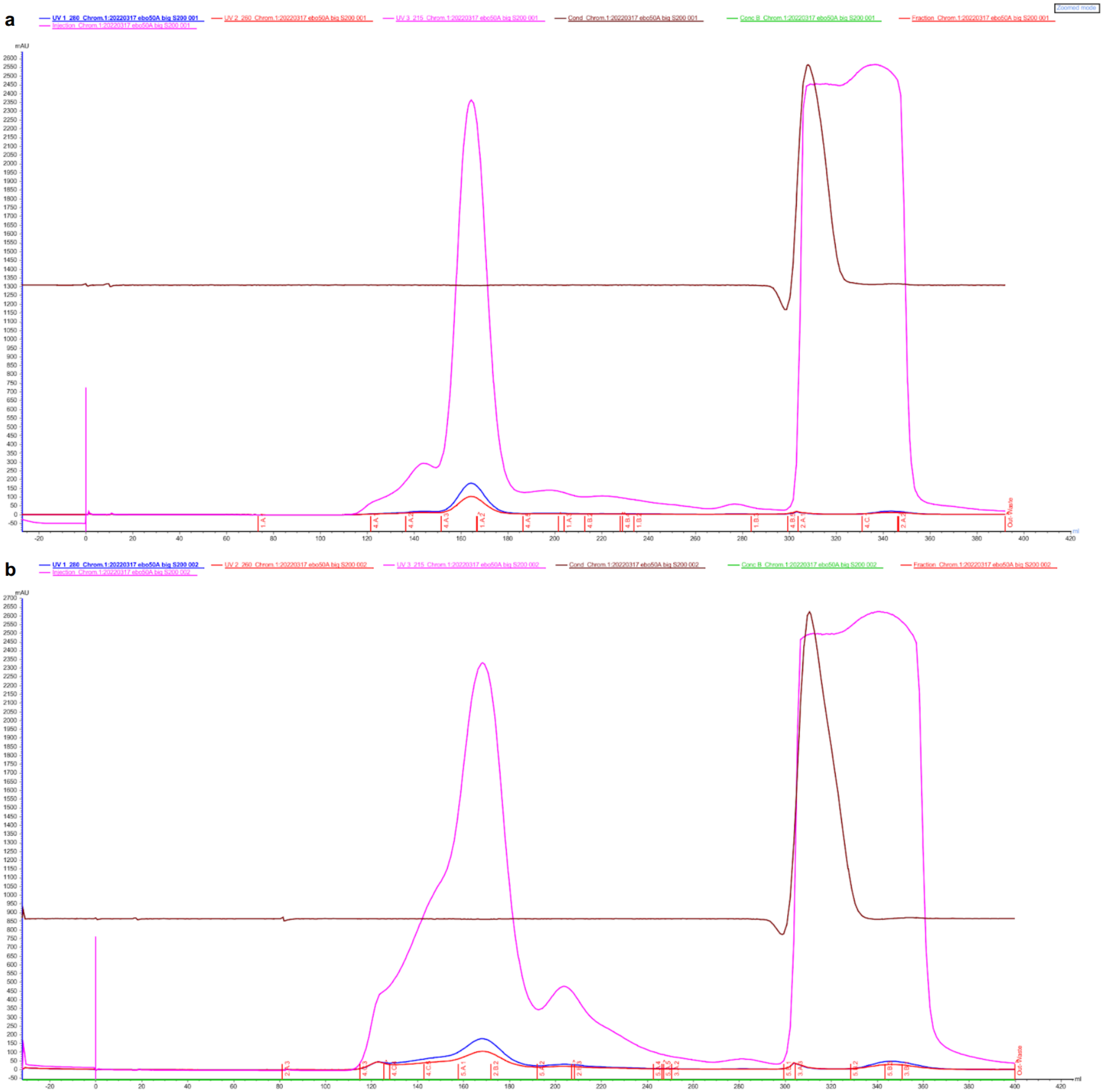

Supplemental Figure 10. Preparative SEC Purification EBOV-GP-I53-50A (MDT-000868 pCMV/R) and SUDV-GP-I53-50A (MDT-000869 pCMV/R) expressed in ExpiHEK293F by Transient Transfection. Constructs were expressed as soluble secreted proteins from ExpiHEK293F cells by transient transfection and the secreted protein was purified from culture medium by IMAC and SEC (HiLoad 26/600 Superdex 200 pg), with absorbance at 280 nm (blue), 215 nm (pink), 260 (red) and conductivity (brown) trailing peak at ~300 minutes represents the salt and imidazole separation from protein previously purified by IMAC. EBOV-GP-I53-50A (MDT-000868 pCMV/R) was produced in 1.6 liters of ~4x10^6 ExpiHEK293F cells/mL and produced a total of 11.6 mg of pure protein following IMAC and SEC purification for a total yield of 7.3 mg/L of cell culture. SUDV-GP-I53-50A (MDT-000869 pCMV/R) was produced in 1.6 liters of ~4x10^6 HEK293F cells/mL and produced a total of ~12.1 mg of pure protein following IMAC and SEC purification for a total yield of 7.6 mg/L of cell culture. **a.** SEC traces for EBOV-GP-I53-50A **b.** SEC traces for SUDV-GP-I53-50A. SUDV-GP-I53-50A (MDT-000869 pCMV/R) was produced in 1.6 liters of ~4x10^6 HEK293F cells/mL and produced a total of ~12.1 mg of pure protein following IMAC and SEC purification for a total yield of 7.6 mg/L of cell culture.

Protein Name:	MDT-000868-I53-50 (Group 1)
Date of Manufacture:	191120
Dilution Recommendation:	1:1 with Addavax
Formulation:	25 mM Tris pH 8, 150 mM NaCl, 5% glycerol
Storage Condition:	-80 °C

Dosing chart

Group	1
Immunogen	868-I53-50
Ag target dose (ug)	2.5
Ag/total protein (% by mass)	0.74
Total protein dose (ug)	3.4
Concentration (mg/mL)	0.034
Volume required per dose (uL)	100
Volume Addavax required per dose (uL)	100
Volume immunogen per vial (uL)	750
Volume Addavax to add per vial (uL)	750
#vials (includes one contingency vial)	5
Total protein (in all vials) (ug)	126

Test	Result
Appearance	Clear solution, with no particles present
Protein Concentration (A_{280})	0.35 μM, 34 ug/mL
Extinction Coefficient (molar)	99905
Predicted Molecular Weight	97098 Da
Endotoxin	<22 EU/mL

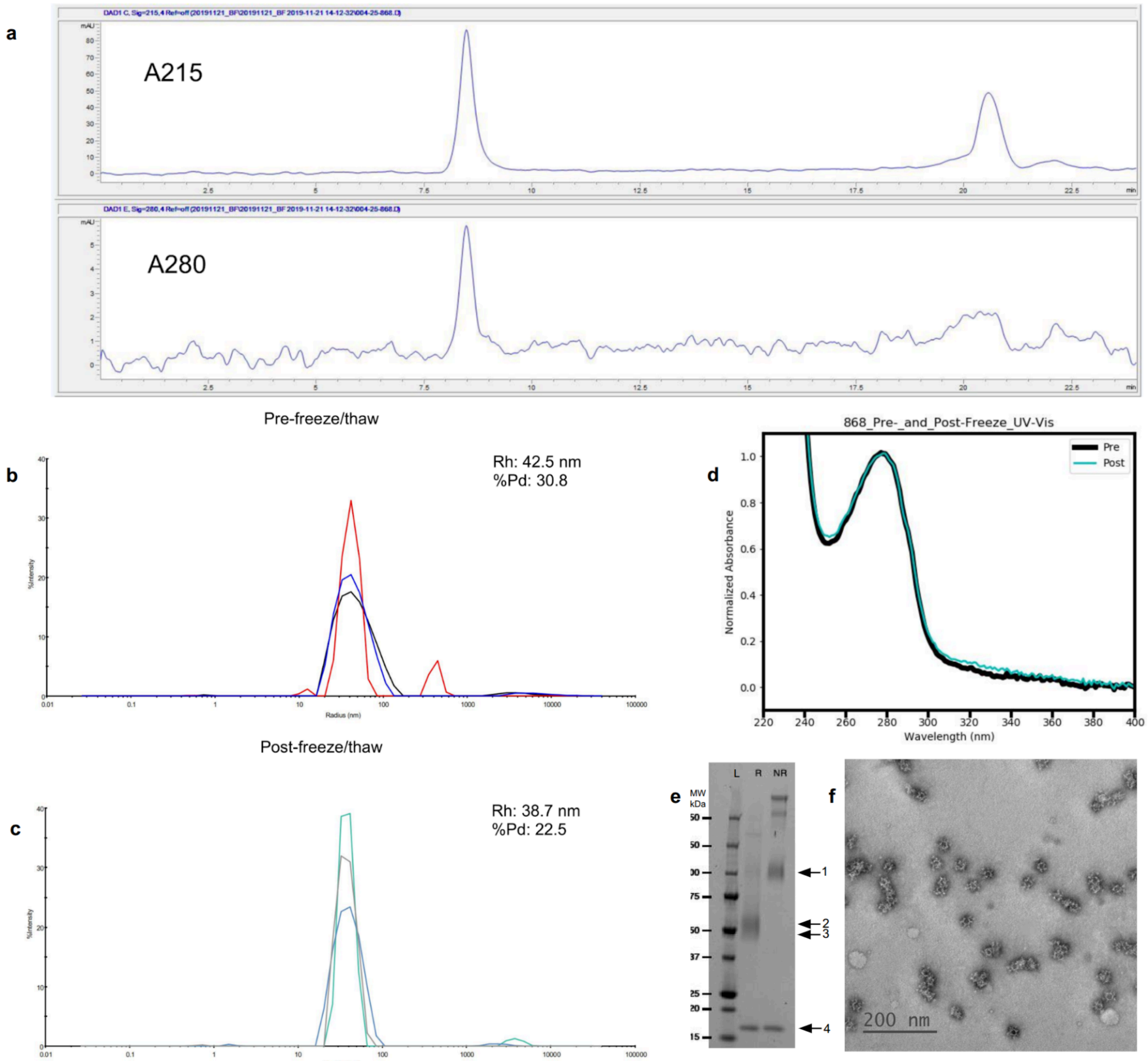

Supplemental Figure 11. EBOV-GP-I53-50 Nanoparticle Immunogens were prepared for the maEBOV study in November 2019 by mixing purified I53-5B.4PT1 protein with IMAC-SEC purified EBOV-GP-I53-50A MDT-000868 produced by pCMV/R transient transfection using ExpiHEK293F cells. **Top tables.** Certificate of testing summary information for immunogen material (in buffer 25 mM Tris pH 8, 150 mM NaCl, 5% glycerol) used in animal immunizations is noted in tabular form above figure panels. **a.** The EBOV-GP-I53-50 NP material was subjected to freeze thaw and then characterized by SEC-HPLC Superose 6 Increase 10/300 GL column with UV trace monitoring at 215 nm and A280 nm, 50 μg load **b.** The nanoparticles were characterized by dynamic light scattering in triplicate pre (red, blue, black) **c.** and post freeze thaw also in triplicate (green, light blue, gray) **d.** The nanoparticles were characterized by UV-Visible wavelength absorbance scan before and after freeze thaw, prefreeze (black) and post freeze-thaw (teal) **e.** SEC-purified nanoparticles were analyzed by SDS-PAGE (Criterion 4–20% Tris-Glycine TGX Stain-Free gel; 300 V for 20 minutes) under reducing (R) and non-reducing (NR) conditions with ladder (L) in lane 1 with molecular weight (MW) markers in kilodaltons (kDa). Bands corresponding to each component were observed at the expected molecular weights, confirming correct expression and assembly. Arrows indicate expected mass locations for band 1: GP1-(ΔMucin)-GP2-(ΔMPER-TM)-I53-50A (lane 3); Band 2: GP1-(ΔMucin) (lane 2); Band 3: GP2-(ΔMPER-TM)-I53-50A (lane 2); Band 4: I53-50B (lanes 2 & 3) **f.** Post-freeze-thaw nanoparticle immunogen samples were examined by negative-stain EM. Representative micrographs show intact, uniformly assembled nanoparticles consistent with the designed I53-50 geometry.

Protein Name: MDT-000869-I53-50 (Group 2)
Date of Manufacture: 191120 Lot 191120 SPW
Dilution Recommendation: 1:1 with Addavax
Formulation: 25 mM Tris pH 8, 150 mM NaCl, 5% glycerol
Storage Condition: -80 °C

Dosing chart

Group	2
Immunogen	869-I53-50
Ag target dose (ug)	2.5
Ag/total protein (% by mass)	0.74
Total protein dose (ug)	3.4
Concentration (mg/mL)	0.034
Volume required per dose (uL)	100
Volume Addavax required per dose (uL)	100
Volume immunogen per vial (uL)	750
Volume Addavax to add per vial (uL)	750
#vials (includes one contingency vial)	5
Total protein (in all vials) (ug)	126

Test	Result
Appearance	Clear solution, with no particles present
Protein Concentration (A_{280})	0.35 μM, 34 ug/mL
Extinction Coefficient (molar)	101395
Predicted Molecular Weight	96945 Da
Endotoxin	<19 EU/mL

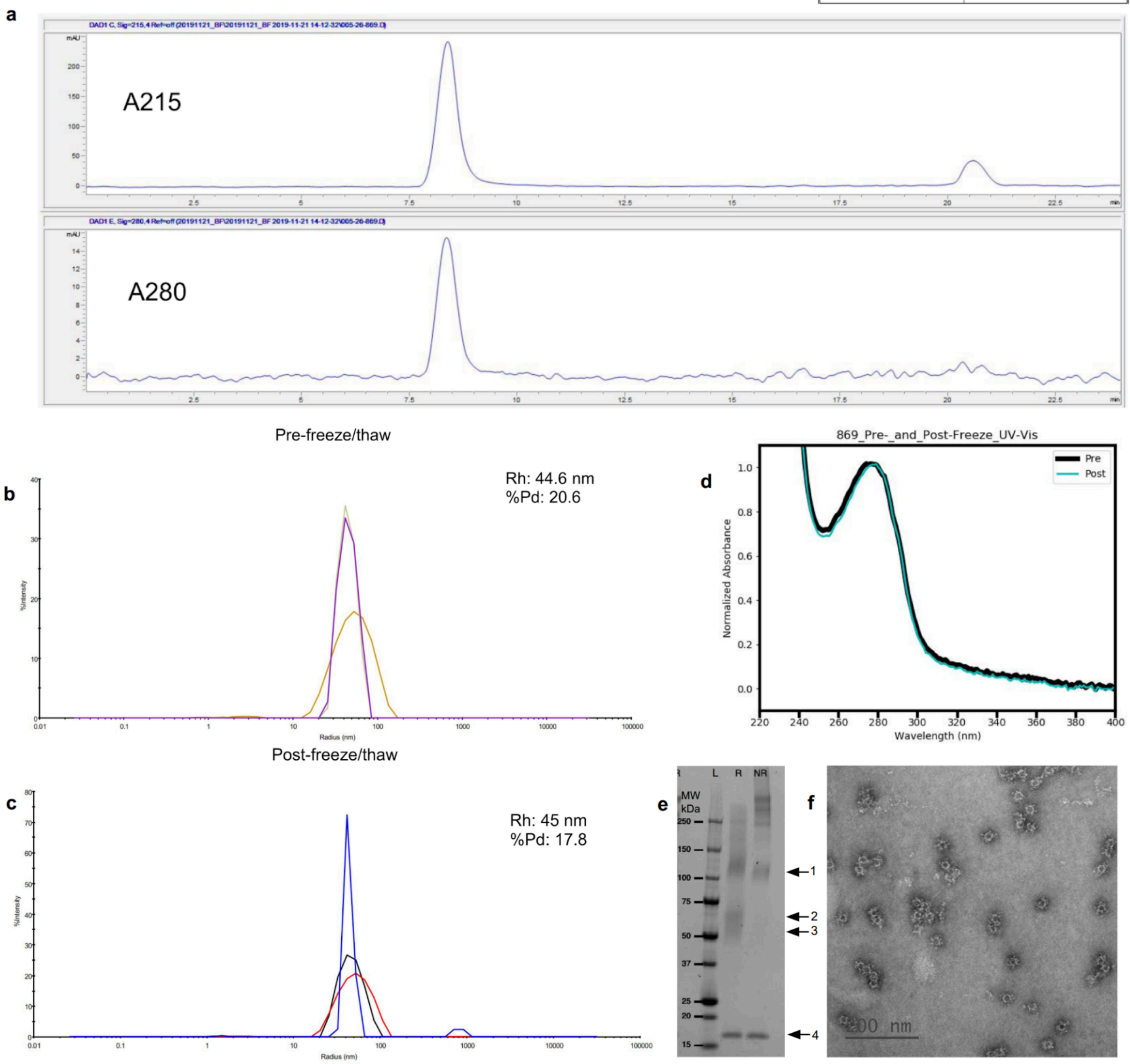

Supplemental Figure 12. SUDV-GP-I53-50 Nanoparticle Immunogens were prepared for the maEBOV study in November of 2019 by mixing purified I53-5B.4PT1 protein with IMAC-SEC purified SUDV-GP-I53-50A MDT-000869 produced by pCMV/R transient transfection using ExpiHEK293F cells. **Top tables.** Certificate of testing summary information for immunogen material (in buffer 25 mM Tris pH 8, 150 mM NaCl, 5% glycerol) used in animal immunizations is noted in tabular form above figure panels. **a.** The SUDV-GP-I53-50 NP material was subjected to freeze thaw and then characterized by SEC-HPLC Superose 6 Increase 10/300 GL column with UV trace monitoring at 215 nm and A280 nm, 50 μg load **b.** The nanoparticles were characterized by dynamic light scattering in triplicate pre (tan, yellow, magenta) **c.** and post freeze thaw (blue, black, red) **d.** The nanoparticles were characterized by UV-Visible wavelength absorbance scan pre (black) and post freeze thaw (teal) **e.** SEC-purified nanoparticles were analyzed by SDS-PAGE (Criterion 4–20% Tris-Glycine TGX Stain-Free gel; 300 V for 20 minutes) under reducing (R) and non-reducing (NR) conditions with ladder (L) in lane 1 with molecular weight (MW) markers in kilodaltons (kDa). Bands corresponding to each component were observed at the expected molecular weights, confirming correct expression and assembly. Arrows indicate expected mass locations for band 1: GP1-(ΔMucin)-GP2-(ΔMPER-TM)-I53-50A (lane 3); Band 2: GP1-(ΔMucin) (lane 2); Band 3: GP2-(ΔMPER-TM)-I53-50A (lane 2); Band 4. I53-50B (lanes 2 & 3) **f.** Post-freeze-thaw nanoparticle immunogen samples were examined by negative-stain EM. Representative micrographs show intact, uniformly assembled nanoparticles consistent with the designed I53-50 geometry.

Protein Name: MDT-000868_MDT-000869_I53-50 mosaic (Group 3)

Date of Manufacture:	191120
Dilution Recommendation:	1:1 with Addavax
Formulation:	25 mM Tris pH 8, 150 mM NaCl, 5% glycerol
Storage Condition:	-80 °C

Dosing chart

Group	3
Immunogen	868-869-I53-50 mosaic
Ag target dose (ug)	2.5
Ag/total protein (% by mass)	0.74
Total protein dose (ug)	3.4
Concentration (mg/mL)	0.034
Volume required per dose (uL)	100
Volume Addavax required per dose (uL)	100
Volume immunogen per vial (uL)	750
Volume Addavax to add per vial (uL)	750
#vials (includes one contingency vial)	5
Total protein (in all vials) (ug)	126

Test	Result
Appearance	Clear solution, with no particles present
Protein Concentration (A_{280})	0.35 µM, 35 ug/mL
Extinction Coefficient (molar)	100650
Predicted Molecular Weight	97021.5 Da
Endotoxin	<23 EU/mL

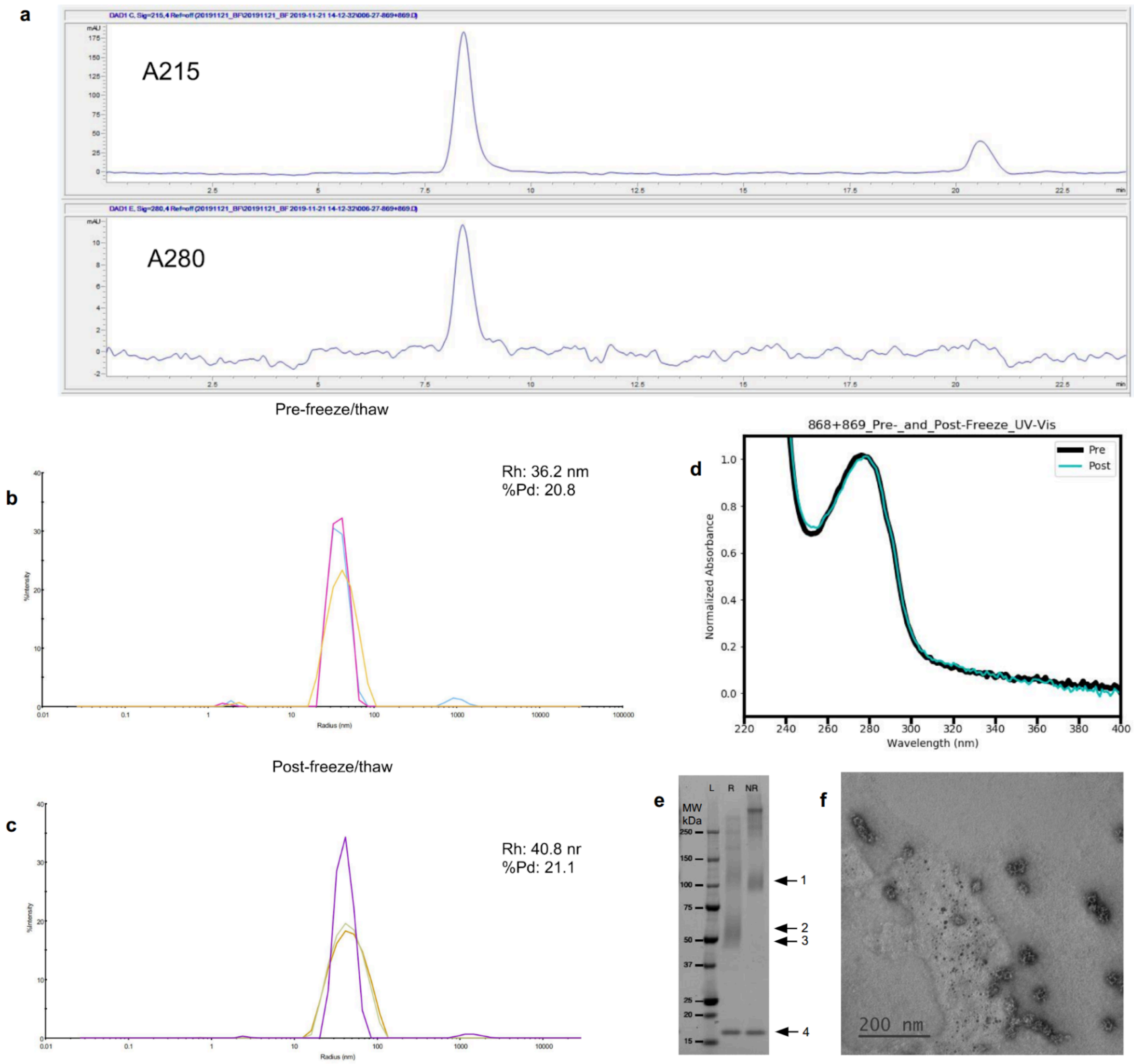

Supplemental Figure 13. Mosaic EBOV/SUDV-GP-I53-50 Nanoparticle Immunogens were prepared for the maEBOV study in November of 2019 by mixing purified I53-5B.4PT1 protein with IMAC-SEC purified EBOV-GP-I53-50A MDT-000868 and SUDV-GP-I53-50A MDT-000869 both produced by pCMV/R transient transfection using ExpiHEK293F cells. **Top tables.** Certificate of testing summary information for immunogen material (in buffer 25 mM Tris pH 8, 150 mM NaCl, 5% glycerol) used in animal immunizations is noted in tabular form above figure panels. **a.** The Mosaic EBOV-SUDV-GP-I53-50 NP material was subjected to freeze thaw and then characterized by SEC-HPLC Superose 6 Increase 10/300 GL column with UV trace monitoring at 215 nm and A280 nm, 50 µg load **b.** The nanoparticles were characterized by dynamic light scattering in triplicate before (pink, light blue, yellow) **c.** and after freeze thaw in triplicate (purple, tan, yellow) **d.** The nanoparticles were characterized by UV-Visible wavelength absorbance scan pre (black) and post freeze thaw (teal) **e.** SEC-purified nanoparticles were analyzed by SDS-PAGE (Criterion 4–20% Tris-Glycine TGX Stain-Free gel; 300 V for 20 minutes) under reducing (R) and non-reducing (NR) conditions with ladder (L) in lane 1 with molecular weight (MW) markers in kilodaltons (kDa). Bands corresponding to each component were observed at the expected molecular weights, confirming correct expression and assembly. Arrows indicate expected mass locations for band 1: GP1-(ΔMucin)-GP2-(ΔMPER-TM)-I53-50A (lane 2); Band 2: GP1-(ΔMucin) (lane 2); Band 3: GP2-(ΔMPER-TM)-I53-50A (lane 2); Band 4. I53-50B (lane 2 & 3) **f.** Post-freeze-thaw nanoparticle immunogen samples were examined by negative-stain EM. Representative micrographs show intact, uniformly assembled nanoparticles consistent with the designed I53-50 geometry.

Protein Name: MDT-000868+MDT-000869_I53-50 cocktail (Group 4)
Date of Manufacture: 191120 Lot 191120 SPW
Dilution Recommendation: 1:1 with Addavax
Formulation: 25 mM Tris pH 8, 150 mM NaCl, 5% glycerol
Storage Condition: -80 °C

Dosing chart

**Group**	4
**Immunogen**	868+869-I53-50 cocktail
**Ag target dose (ug)**	2.5
**Ag/total protein (% by mass)**	0.74
**Total protein dose (ug)**	3.4
**Concentration (mg/mL)**	0.034
**Volume required per dose (uL)**	100
**Volume Addavax required per dose (uL)**	100
**Volume immunogen per vial (uL)**	750
**Volume Addavax to add per vial (uL)**	750
**#vials (includes one contingency vial)**	5
**Total protein (in all vials) (ug)**	126

Test	Result
Appearance	Clear solution, with no particles present
Protein Concentration (A_{280})	0.35 µM, 35 ug/mL
Extinction Coefficient (molar)	100650
Predicted Molecular Weight	97021.5 Da
Endotoxin	<23 EU/mL

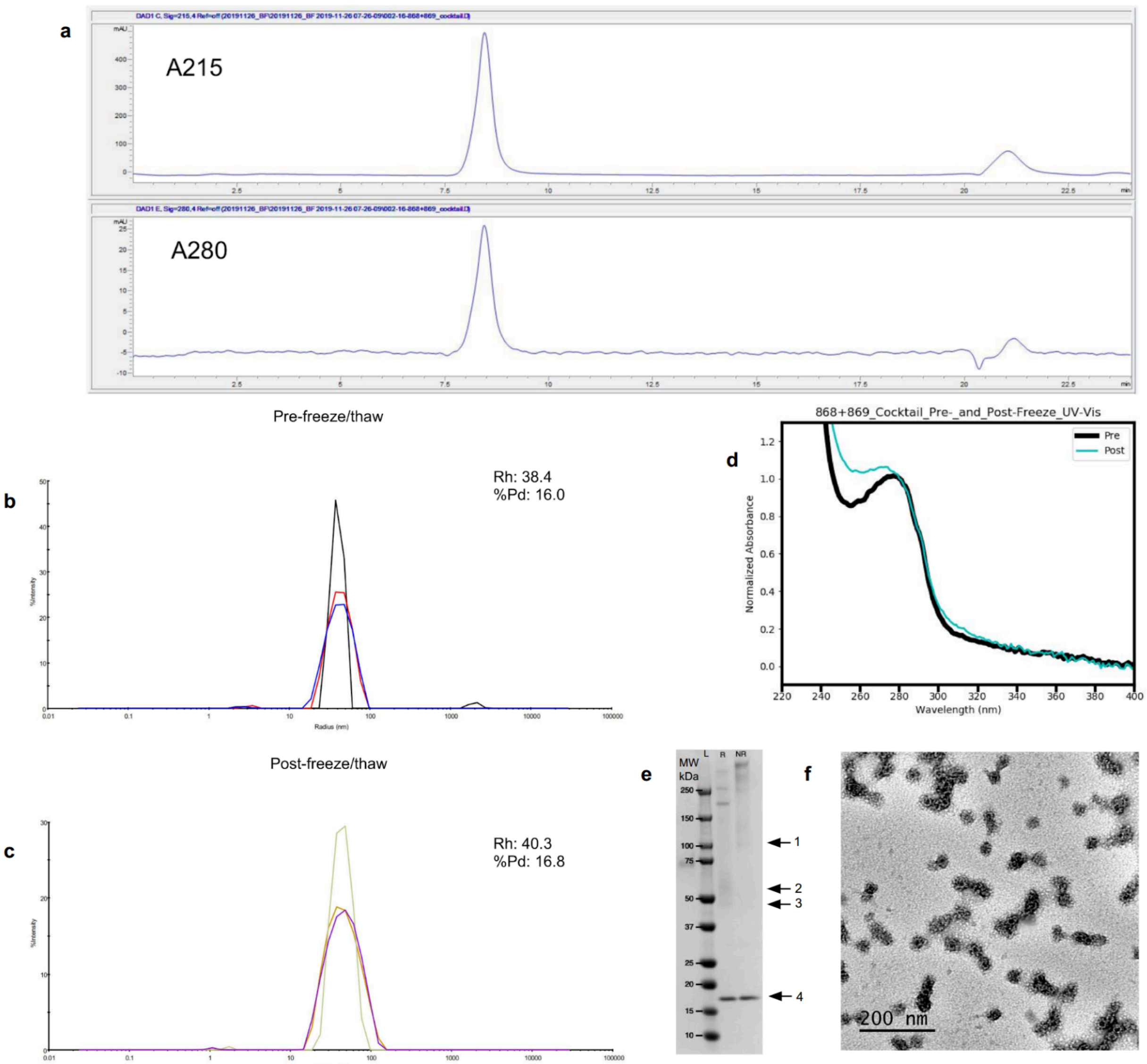

Supplemental Figure 14. Cocktail Mix of EBOV-GP-I53-50 and SUDV-GP-I53-50 Nanoparticle Immunogens were individually prepared for the maEBOV study in November of 2019 (Supplemental Figures 11 and 12) and then mixed 1:1. **Top tables.** Certificate of testing summary information for immunogen material (in buffer 25 mM Tris pH 8, 150 mM NaCl, 5% glycerol) used in animal immunizations is noted in tabular form above figure panels. **a.** The cocktail nanoparticle immunogen was subjected to freeze thaw and then characterized by SEC-HPLC Superose 6 Increase 10/300 GL column with UV trace monitoring at 215 nm and A280 nm, 50 µg load **b.** The nanoparticles were characterized by dynamic light scattering in triplicate pre (black, red, blue) **c.** and post freeze thaw in triplicate (tan, yellow, magenta) **d.** The nanoparticles were characterized by UV-Visible wavelength absorbance scan pre (black) and post freeze thaw (teal) **e.** SEC-purified nanoparticles were analyzed by SDS-PAGE (Criterion 4–20% Tris-Glycine TGX Stain-Free gel; 300 V for 20 minutes) under reducing (R) and non-reducing (NR) conditions with ladder (L) in lane 1 with molecular weight (MW) markers in kilodaltons (kDa). Bands corresponding to each component were observed at the expected molecular weights, confirming correct expression and assembly. Arrows indicate expected mass locations for band 1: GP1-(ΔMucin)-GP2-(ΔMPER-TM)-I53-50A (lane 3); Band 2: GP1-(ΔMucin) (lane 2); Band 3: GP2-(ΔMPER-TM)-I53-50A (lane 2); Band 4: I53-50B (lane 2 and 3) **f.** Post-freeze-thaw nanoparticle immunogen samples were examined by negative-stain EM. Representative micrographs show intact, uniformly assembled nanoparticles consistent with the designed I53-50 geometry.

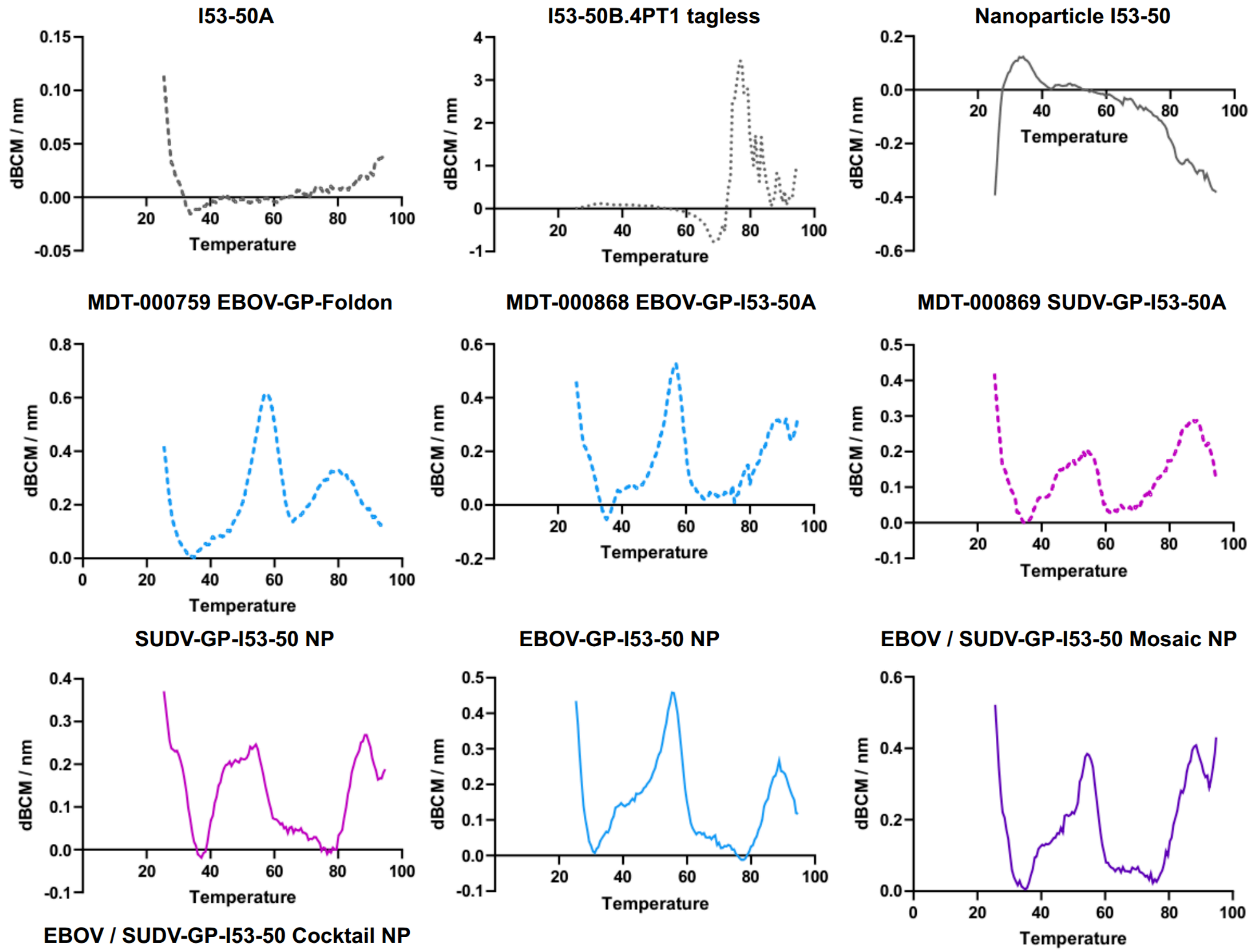

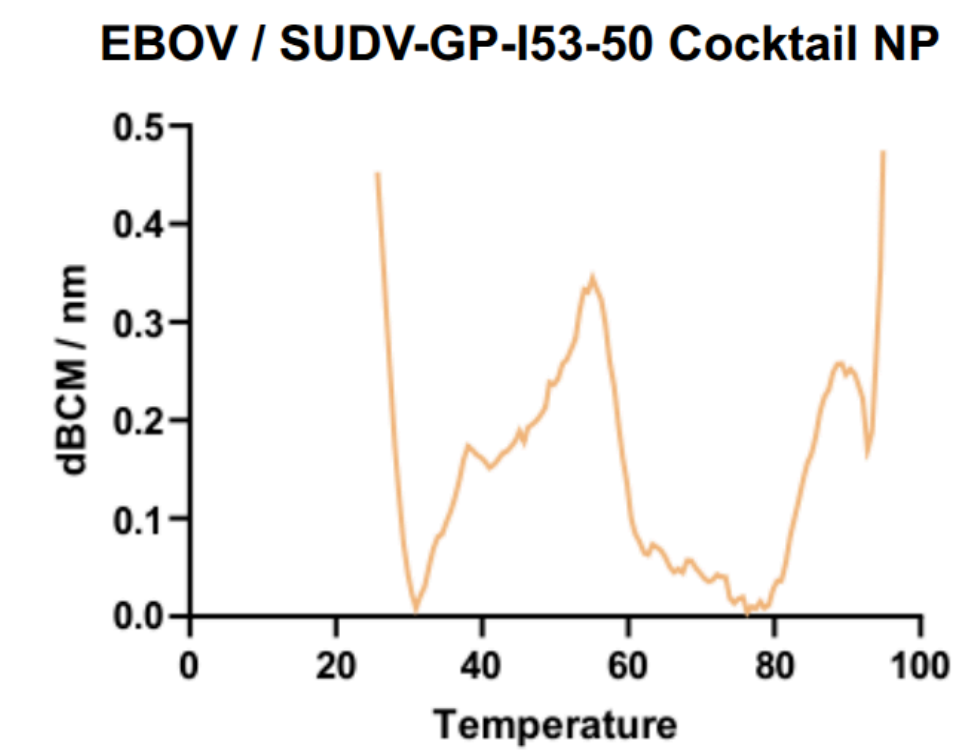

Components	TM1 °C	TM2 °C
I53-50B.4PT1	76.8	
MDT-000759 EBOV-GP-Foldon	57.5	80.3
MDT-000868 EBOV-GP-I53-50A	56.9	89
MDT-000869 SUDV-GP-I53-50A	54.1	87.3
**Nanoparticles**	**TM1 °C**	**TM2 °C**
SUDV-GP-I53-50 NP	54.3	88.3
EBOV-GP-I53-50 NP	55.3	88.8
EBOV/SUDV-GP-I53-50 Mosaic NP	55.6	88.5
EBOV/SUDV-GP-I53-50 Cocktail NP	55.1	89.2

Supplemental Figure 15. Thermal stability of EBOV- and SUDV-GP nanoparticle immunogens by differential scanning fluorimetry. EBOV-GP-I53-50 and SUDV-GP-I53-50 nanoparticle component fusions and assembled nanoparticles (0.10 mg/mL) were analyzed by intrinsic tryptophan fluorescence using a UNcle instrument (Unchained Labs) to determine melting transitions (Tm). Fluorescence intensity ratios at 330 nm and 350 nm were monitored as a function of temperature from 25°C to 95°C (0.15°C min^{-1}; acquisition interval = 10 seconds). The I53-50A component exhibited no detectable unfolding across the full temperature range, while I53-50B showed a gradual transition beginning near 80°C. Upon assembly, the I53-50 nanoparticle remained stable with no unfolding transitions observed for either component, indicating enhanced stability upon assembly. In contrast, GP-containing components and nanoparticles exhibited a biphasic melting (Tm_1 ≈ 55°C and (Tm_2 ≈ 80–88°C). Reported Tm values represent the mean of 10 replicate acquisitions per sample.

Protein Name: MDT-000868+2obx non-assembling control (Group 5)
Date of Manufacture: 191120
Dilution Recommendation: 1:1 with Addavax
Formulation: 25 mM Tris pH 8, 150 mM NaCl, 5% glycerol
Storage Condition: -80 °C

Test	Result
Appearance	Clear solution, with no particles present
Protein Concentration (A_{280})	0.34 µM, 33 ug/mL
Extinction Coefficient (molar)	101395
Predicted Molecular Weight	96169 Da
Endotoxin	<15 EU/mL

Group	5
Immunogen	868+2obx
Ag target dose (ug)	2.5
Ag/total protein (% by mass)	0.75
Total protein dose (ug)	3.3
Concentration (mg/mL)	0.033
Volume required per dose (uL)	100
Volume Addavax required per dose (uL)	100
Volume immunogen per vial (uL)	750
Volume Addavax to add per vial (uL)	750
#vials (includes one contingency vial)	5
Total protein (in all vials) (ug)	125

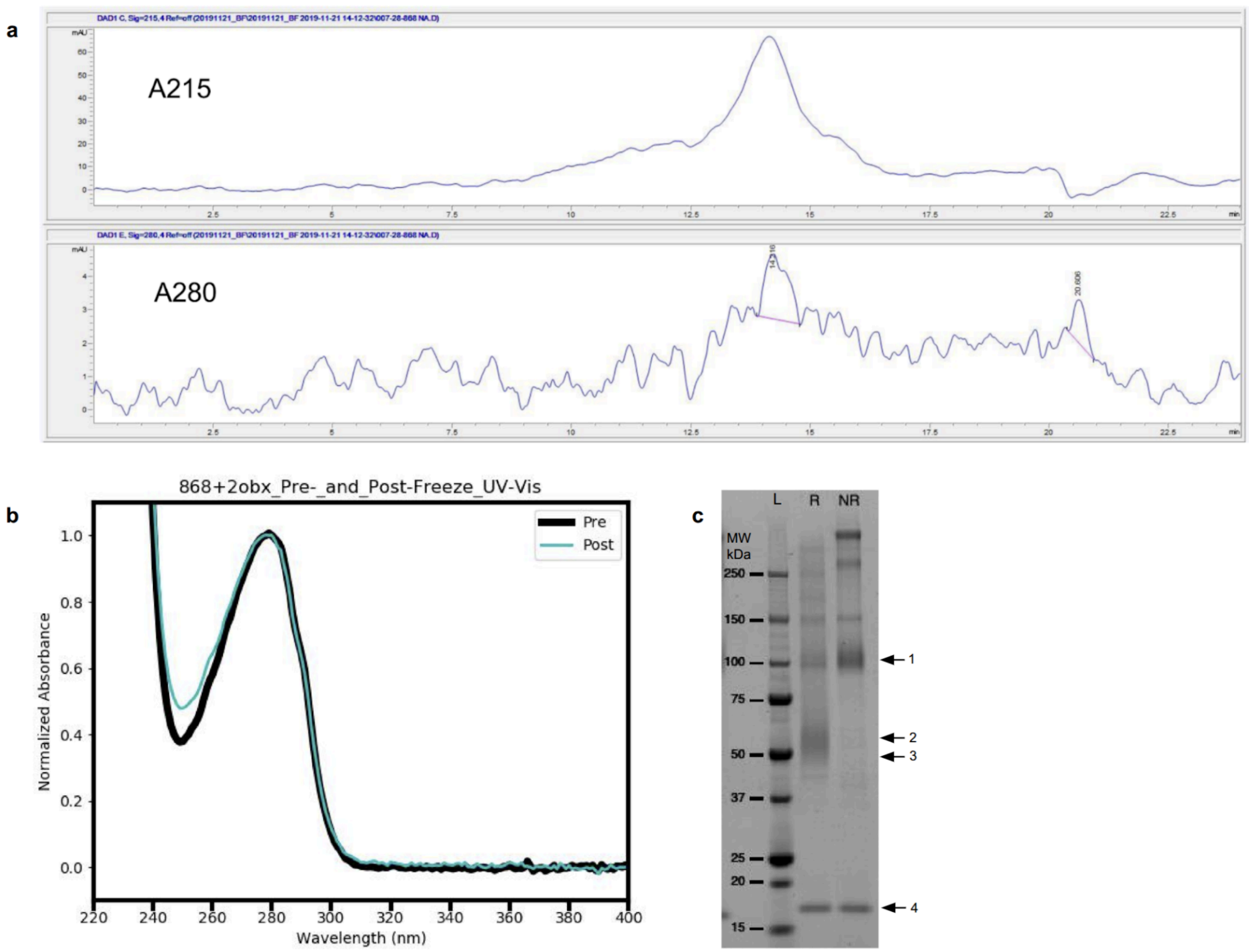

Supplemental Figure 16. Non-Assembling EBOV-GP-I53-50A + 2OBX control Immunogens were prepared for the maEBOV study in November of 2019 by mixing purified 2OBX protein with IMAC-SEC purified EBOV-GP-I53-50A MDT-000868 produced by pCMV/R transient transfection using ExpiHEK293F cells. **Top tables.** Certificate of testing summary information for immunogen material (in buffer 25 mM Tris pH 8, 150 mM NaCl, 5% glycerol) used in animal immunizations is noted in tabular form above figure panels. **a.** The EBOV-GP-I53-50A + 2OBX material was subjected to freeze thaw and then characterized by SEC-HPLC Superose 6 Increase 10/300 GL column with UV trace monitoring at 215 nm and A280 nm, 25 µg load. **b.** The protein mixture was characterized by UV-Visible wavelength absorbance scan before and after freeze-thaw. **c**. The protein mixture was analyzed by SDS-PAGE (Criterion 4-20% Tris-Glycine TGX gel StainFree, 300V for 20 minutes) under reducing (R) and non-reducing (NR) conditions with ladder (L) in lane 1 with molecular weight (MW) markers in kilodaltons (kDa). Arrows indicate expected mass locations for band 1: GP1-(ΔMucin)-GP2-(ΔMPER-TM)-I53-50A (lane 3); Band 2: GP1-(ΔMucin) (lane 1); Band 3: GP2-(ΔMPER-TM)-I53-50A (lane 2); Band 4: I53-50B (lane 2 and 3).

Protein Name: MDT-000869+2obx non-assembling control (Group 6)
Date of Manufacture: 191120
Dilution Recommendation: 1:1 with Addavax
Formulation: 25 mM Tris pH 8, 150 mM NaCl, 5% glycerol
Storage Condition: -80 °C

Test	Result
Appearance	Clear solution, with no particles present
Protein Concentration (A_{280})	0.34 μM, 33 ug/mL
Extinction Coefficient (molar)	102885
Predicted Molecular Weight	96015.8 Da
Endotoxin	<15 EU/mL

**Group**	6
**Immunogen**	869+2obx
**Ag target dose (ug)**	2.5
**Ag/total protein (% by mass)**	0.75
**Total protein dose (ug)**	3.3
**Concentration (mg/mL)**	0.033
**Volume required per dose (uL)**	100
**Volume Addavax required per dose (uL)**	100
**Volume immunogen per vial (uL)**	750
**Volume Addavax to add per vial (uL)**	750
**#vials (includes one contingency vial)**	5
**Total protein (in all vials) (ug)**	124

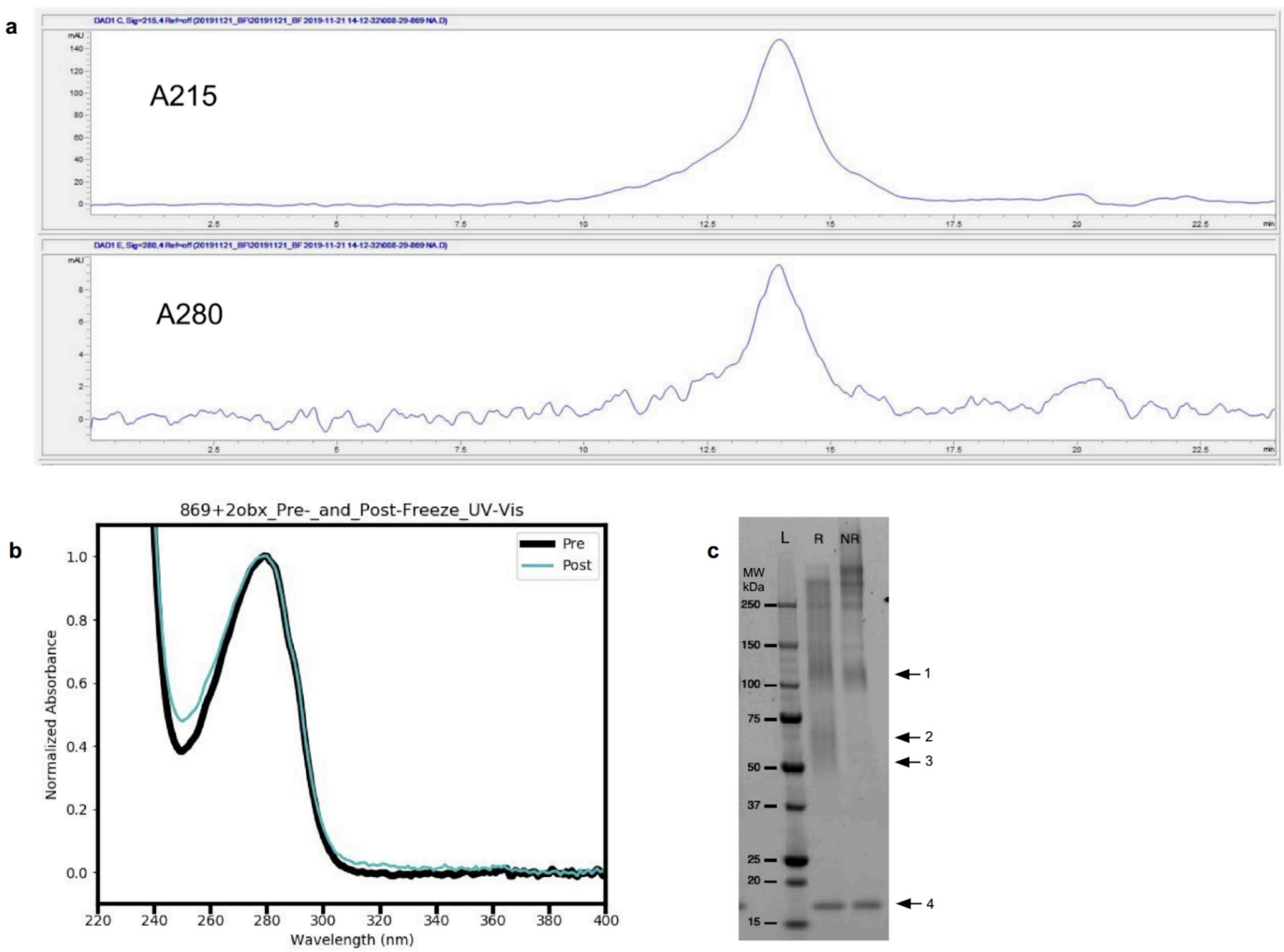

Supplemental Figure 17. SUDV-GP-I53-50 + 2OBX non-assembling control immunogens were prepared for the maEBOV study in November 2019 by mixing purified 2OBX protein with IMAC-SEC purified SUDV-GP-I53-50A MDT-000868 produced by pCMV/R transient transfection using ExpiHEK293F cells. **Top tables.** Certificate of testing summary information for immunogen material (in buffer 25 mM Tris pH 8, 150 mM NaCl, 5% glycerol) used in animal immunizations is noted in tabular form above figure panels. **a.** The SUDV-GP-I53-50A + 2OBX material was characterized by SEC-HPLC Superose 6 Increase 10/300 GL column with UV trace monitoring at 215 nm and A280 nm, 25 µg load **b.** The protein mixture was characterized by UV-Visible wavelength absorbance scan before and after freeze thaw. **c.** The protein mixture was analyzed by SDS-PAGE (Criterion 4-20% Tris-Glycine TGX gel StainFree, 300V for 20 minutes) under reducing (R) and non-reducing (NR) conditions (1.5 µg load) with ladder (L) in lane 1 with molecular weight (MW) markers in kilodaltons (kDa); Arrows indicate expected mass locations for band 1: GP1-(ΔMucin)-GP2-(ΔMPER-TM)-I53-50A (lane 3). Band 2: GP1-(ΔMucin) (lane 2); Band 3: GP2-(ΔMPER-TM)-I53-50A (lane 2); Band 4: I53-50B (lane 2 and 3).

Protein Name: MDT-000868_MDT000869_2obx (Group 7)
Date of Manufacture: 191120
Dilution Recommendation: 1:1 with Addavax
Formulation: 25 mM Tris pH 8, 150 mM NaCl, 5% glycerol
Storage Condition: -80 °C

Test	Result
Appearance	Clear solution, with no particles present
Protein Concentration (A_{280})	0.34 µM, 33 ug/mL
Extinction Coefficient (molar)	102140
Predicted Molecular Weight	96092.3 Da
Endotoxin	<15 EU/mL

**Group**	**7**
**Immunogen**	868+869+2obx
**Ag target dose (ug)**	2.5
**Ag/total protein (% by mass)**	0.75
**Total protein dose (ug)**	3.3
**Concentration (mg/mL)**	0.033
**Volume required per dose (uL)**	100
**Volume Addavax required per dose (uL)**	100
**Volume immunogen per vial (uL)**	750
**Volume Addavax to add per vial (uL)**	750
**#vials**	4
**Total protein (in all vials) (ug)**	125

a

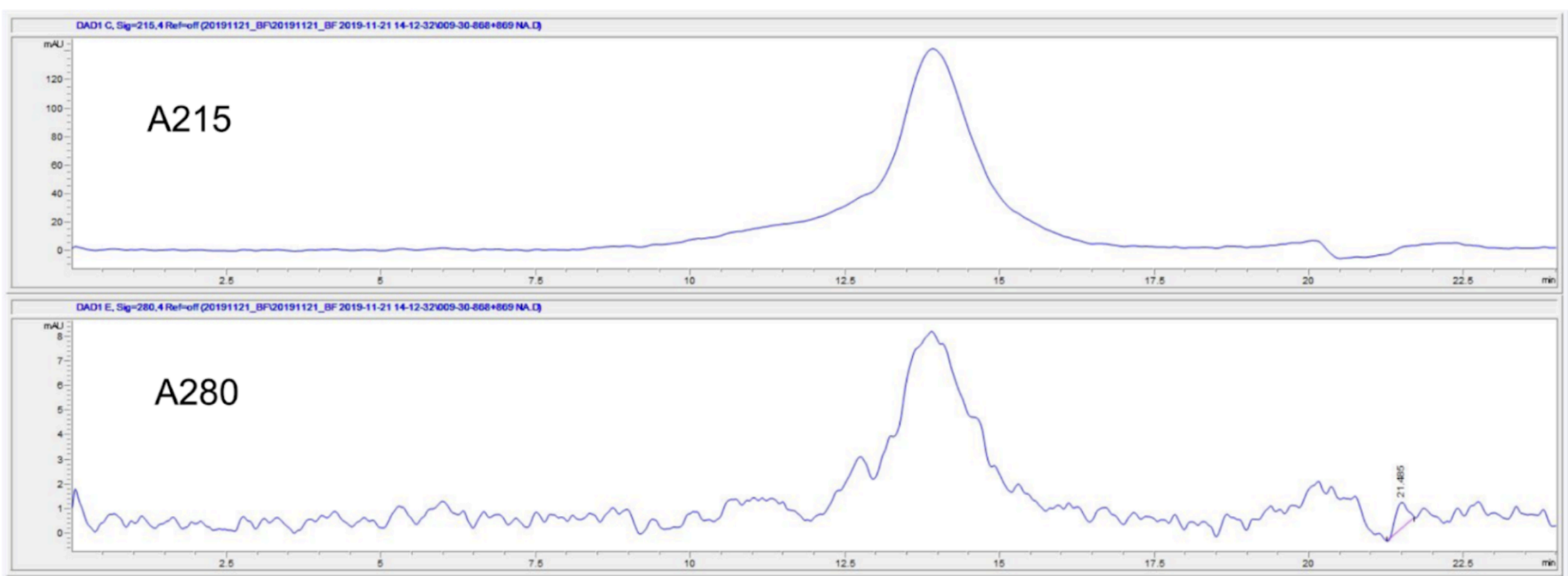

b

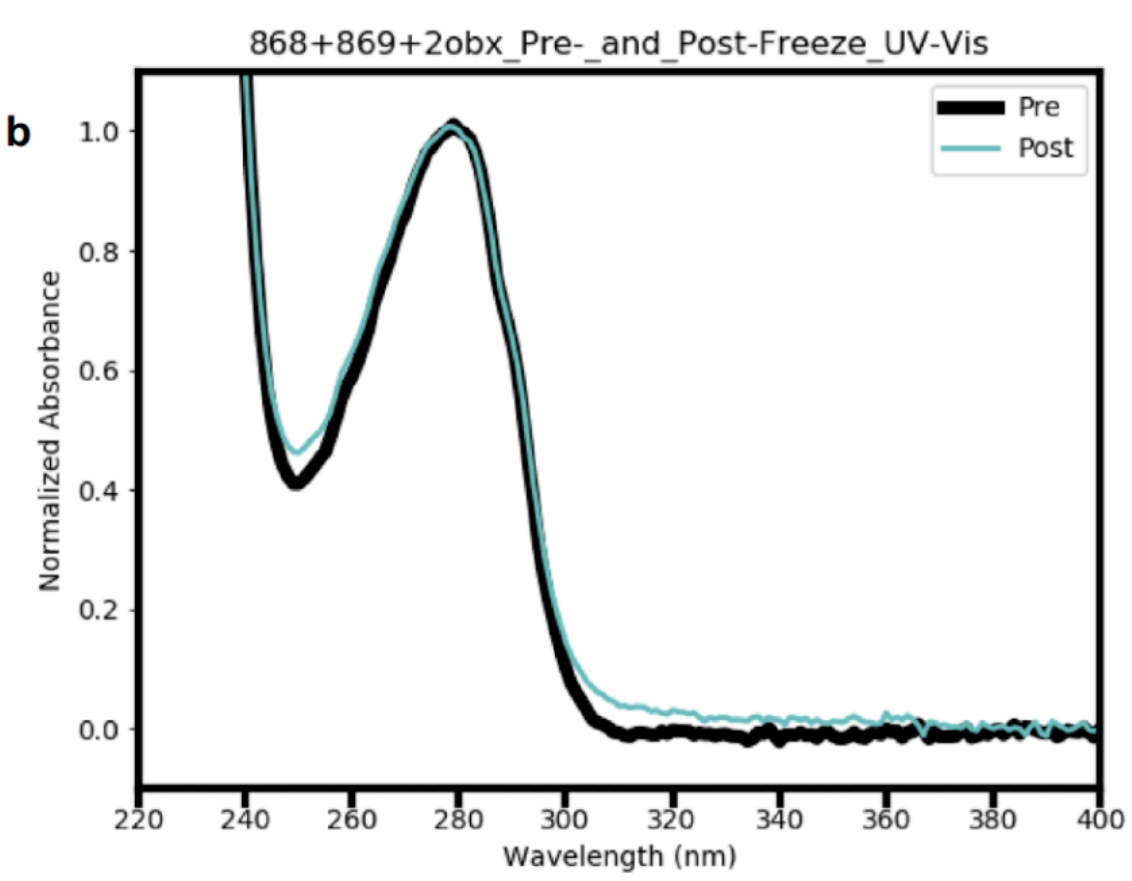

c

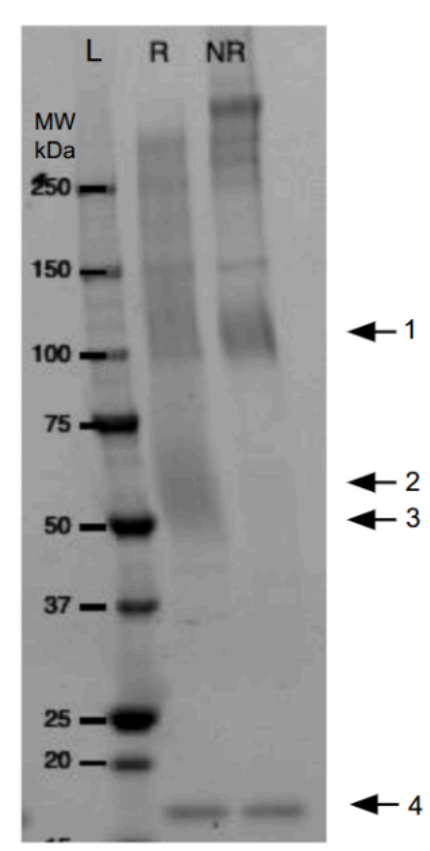

Supplemental Figure 18. EBOV-GP-I53-50 plus SUDV-GP-I53-50 + 2OBX non-assembling control immunogens were prepared for the maEBOV study in November 2019 by mixing purified 2OBX protein with IMAC-SEC purified EBOV-GP-I53-50A MDT-000868 produced by pCMV/R transient transfection using ExpiHEK293F cells. **Top tables.** Certificate of testing summary information for immunogen material (in buffer 25 mM Tris pH 8, 150 mM NaCl, 5% glycerol) used in animal immunizations is noted in tabular form above figure panels. **a.** The EBOV-GP-I53-50A + SUDV-GP-I53-50A + 2OBX material was subjected to freeze thaw and then characterized by SEC-HPLC Superose 6 Increase 10/300 GL column with UV trace monitoring at 215 nm and A280 nm, 50 µg load **b.** The mixture of proteins was characterized by UV-Visible wavelength absorbance scan before and after freeze thaw. **c.** The mixture of proteins was analyzed by SDS-PAGE (Criterion 4-20% Tris-Glycine TGX gel StainFree, 300V for 20 minutes) under reducing (R) and non-reducing (NR) conditions (1.5 µg load) with molecular weight (MW) markers in lane 1 in kilodaltons (kDa). Arrows indicate expected mass locations for band 1: GP1-(ΔMucin)-GP2-(ΔMPER-TM)-I53-50A (lane 3); Band 2: GP1-(ΔMucin) (lane 2); Band 3: GP2-(ΔMPER-TM)-I53-50A (lane 2); Band 4: I53-50B (lane 2 and 3).

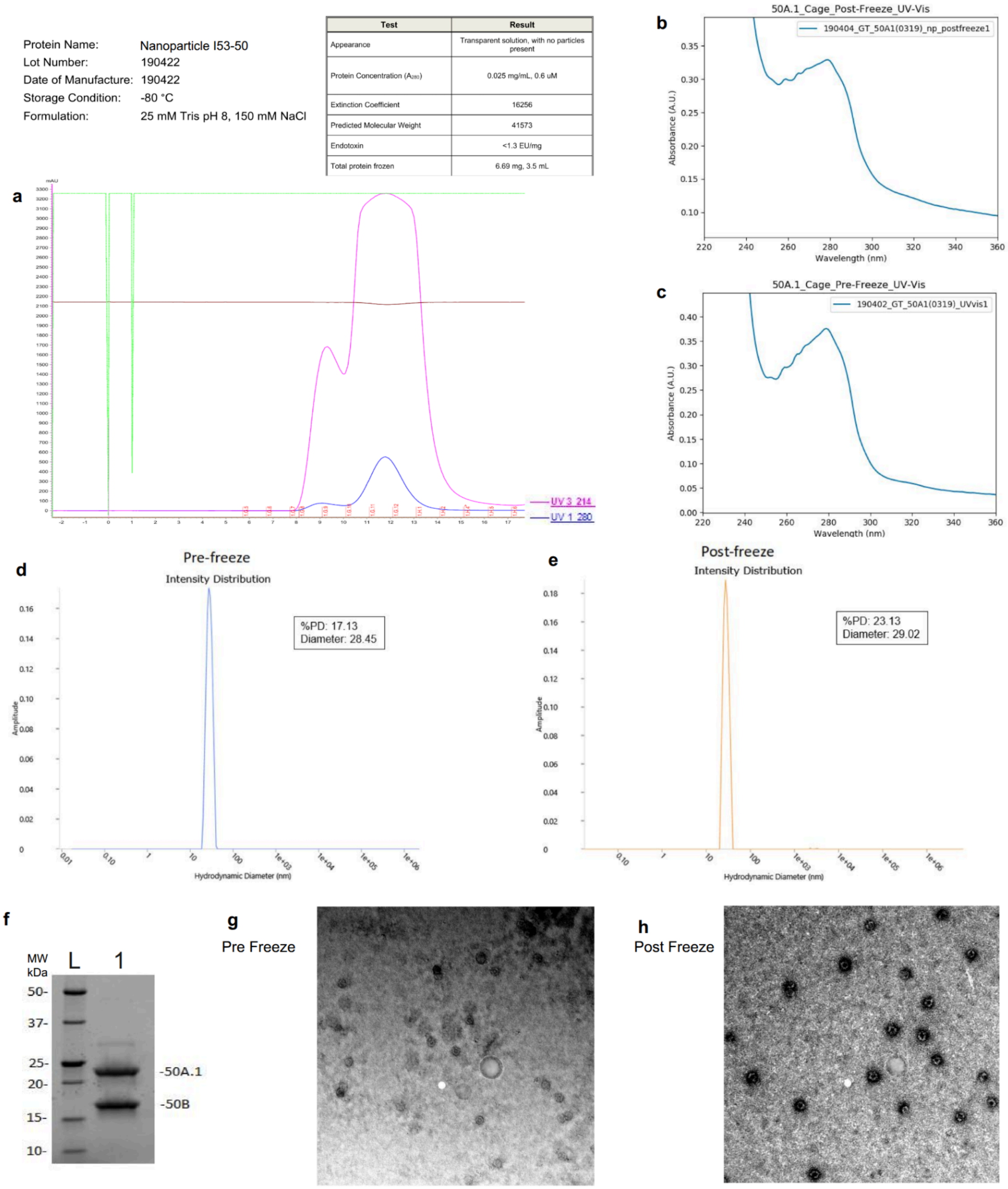

Protein Name:	Nanoparticle I53-50
Lot Number:	190422
Date of Manufacture:	190422
Storage Condition:	-80 °C
Formulation:	25 mM Tris pH 8, 150 mM NaCl

Test	Result
Appearance	Transparent solution, with no particles present
Protein Concentration (A_{280})	0.025 mg/mL, 0.6 uM
Extinction Coefficient	16256
Predicted Molecular Weight	41573
Endotoxin	<1.3 EU/mg
Total protein frozen	6.69 mg, 3.5 mL

Supplemental Figure 19. Characterization of I53-50 nanoparticles used for EBOV and SUDV immunization studies. Nanoparticle I53-50 immunogens were produced by mixing purified I53-50A with I53-50B.4PT1 followed by SEC purification and characterization. The I53-50 nanoparticles were generated as a single lot used for both the maEBOV and gpaSUDV studies. Nanoparticles were formed by mixing the I53-50A and I53-50B.4PT1 components following published procedures[39], and purifying by SEC (25 mM Tris pH 8, 150 mM NaCl) with UV trace monitoring at 215 nm and A280 nm, fractions 10-13 mL were collected and pooled. Top tables. Certificate of testing summary information for immunogen material used in animal immunizations is noted in tabular form above figure panels. **a.** The SEC purified nanoparticles were characterized by **b,c.** UV-Visible wavelength absorbance scan **b.** Before freeze-thaw **c.** After freeze-thaw and centrifugation ~20,000xg for 5 minutes at 4ºC where low absorbance at 350 nm suggests low aggregation. **d,e**. The SEC purified nanoparticles were characterized by dynamic light scattering **d.** before freeze thaw and **e.** after freeze thaw plus centrifugation ~20,000xg for 5 minutes at 4ºC **f.** SEC-purified nanoparticles were analyzed by SDS-PAGE (Criterion 4–20% Tris-Glycine TGX Stain-Free gel; 300 V for 20 minutes) under non-reducing (NR) conditions with molecular-weight markers in lane 1, Molecular weight (MW) markers in kilodaltons (kDa), ladder (L). Bands appeared at the expected molecular weights, confirming correct assembly of each nanoparticle component **g–h.** SEC-purified nanoparticles were characterized by negative-stain EM. Representative micrographs show well-formed, intact nanoparticles with consistent morphology. Images were collected over regions of thin stain, accounting for the darker contrast of the particles relative to other samples analyzed by this method in this study. **g.** Before freeze thaw **h.** After freeze–thaw.

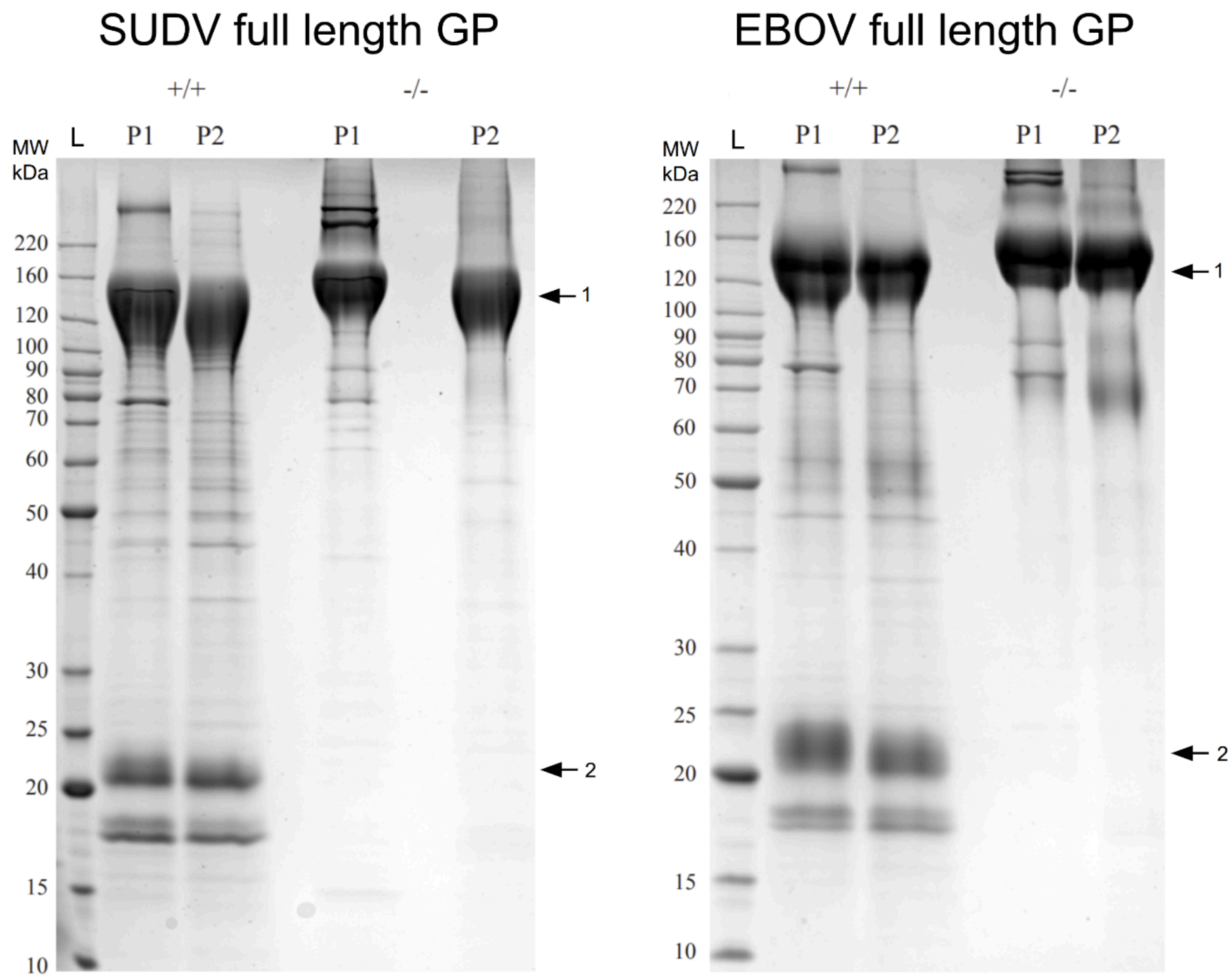

Sample	Pool ID	Total mgs
EBOV full length GP	P1	1.5
EBOV full length GP	P2	188.1
SUDV full length GP	P1	5.2
SUDV full length GP	P2	230.8

Supplemental figure 20. SDS Page gels, SUDV and EBOV full length GP produced for ELISAs. Protein was analyzed by SDS page gel and visualized with coomassie-stain; 5 µg protein was added per lane. Plus/minus indicate whether the sample was heated and reduced prior to loading gel, +/+ = heated and reduced, -/- = not heat/not reduced. Molecular weight (MW) markers in kilodaltons (kDa), ladder (L). P1 is a small-scale purification to test expression levels, and P2 is the purification as described under SUDV and EBOV GP Protein Production for ELISA in methods. The numbered arrows indicate expected mass locations for Band 1 as GP1 and Band 2 as GP2.

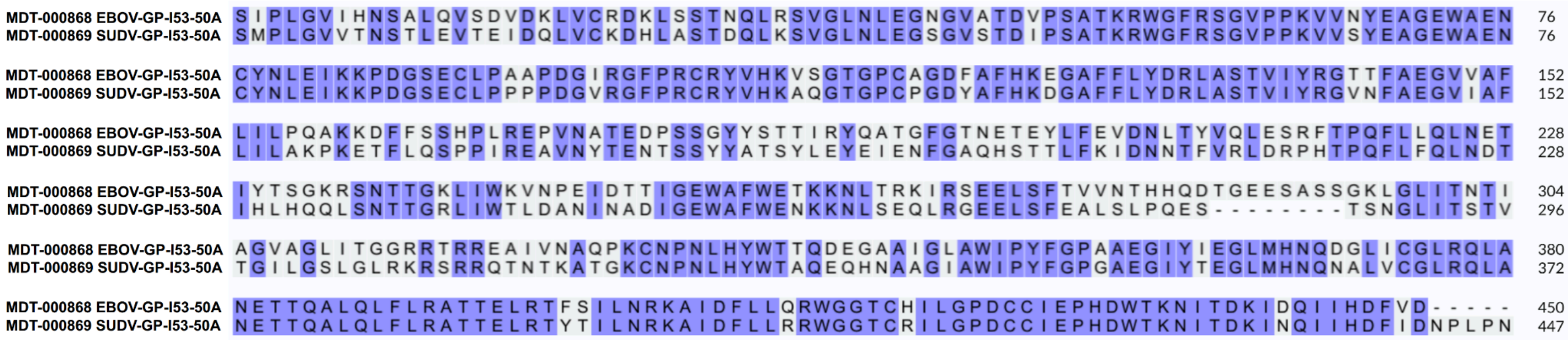

Supplemental Figure 21. Comparative Amino Acid Sequence for Ebola Viral Glycoprotein Immunogens as I53-50A Fusions. The amino acid sequences of the glycoprotein portions of SUDV-GP-I53-50A / MDT-000869 and EBOV-GP-I53-50A / MDT-000868 are aligned with UniProt[116] with sequence identities (blue), gaps (-) and non-identity (grey). There is 68% amino acid sequence identity for the GP portions of these constructs. MDT-000868 = EBOV-GP-I53-50A, MDT-000869 = SUDV-GP-I53-50A.

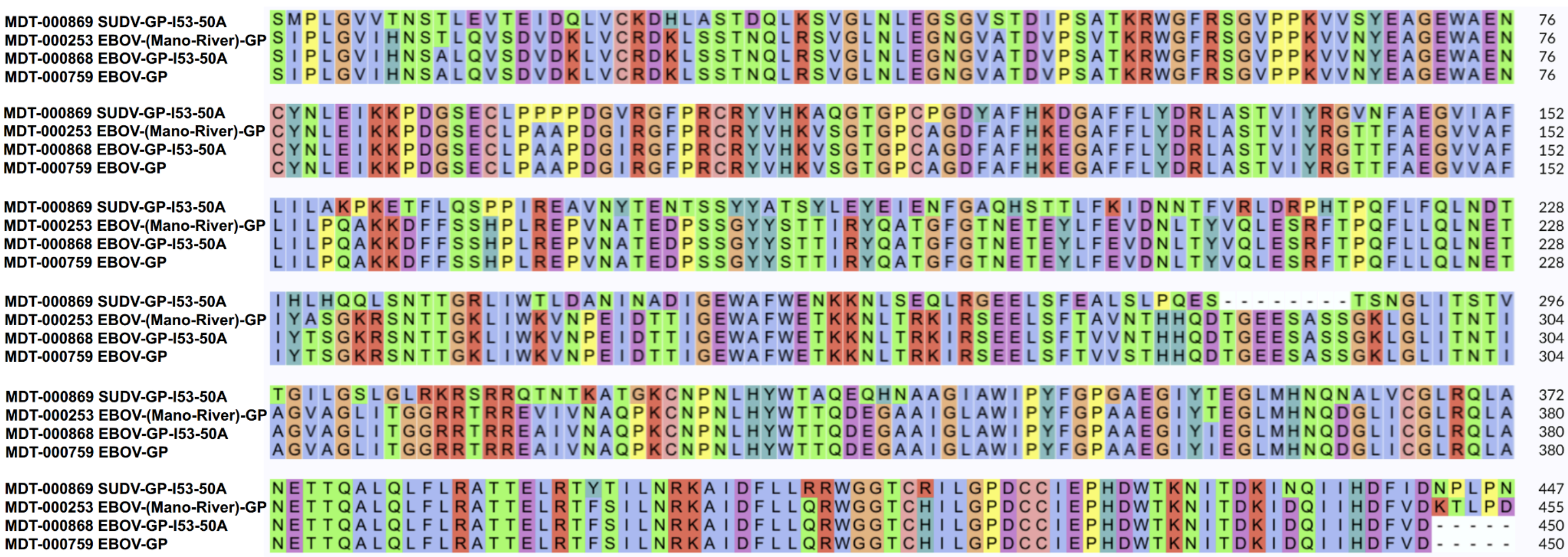

Supplemental Figure 22. Comparative Amino Acid Sequence for Ebola Viral Glycoprotein Components for Constructs in this Report. The glycoprotein amino acid sequences of the glycoprotein portions are aligned with UniProt[116] alignment with Clustal2 colors based on amino acid type, and gaps (-). for four of our constructs: MDT-000869 = SUDV-GP-I53-50A; MDT-000253 = EBOV-GP Mano-River (Flag,∆muc,∆MPER-TM)-I53-50A-Tev-His6; MDT-000868 = EBOV-GP-I53-50A; and MDT-000759 = EBOV-GP.

a

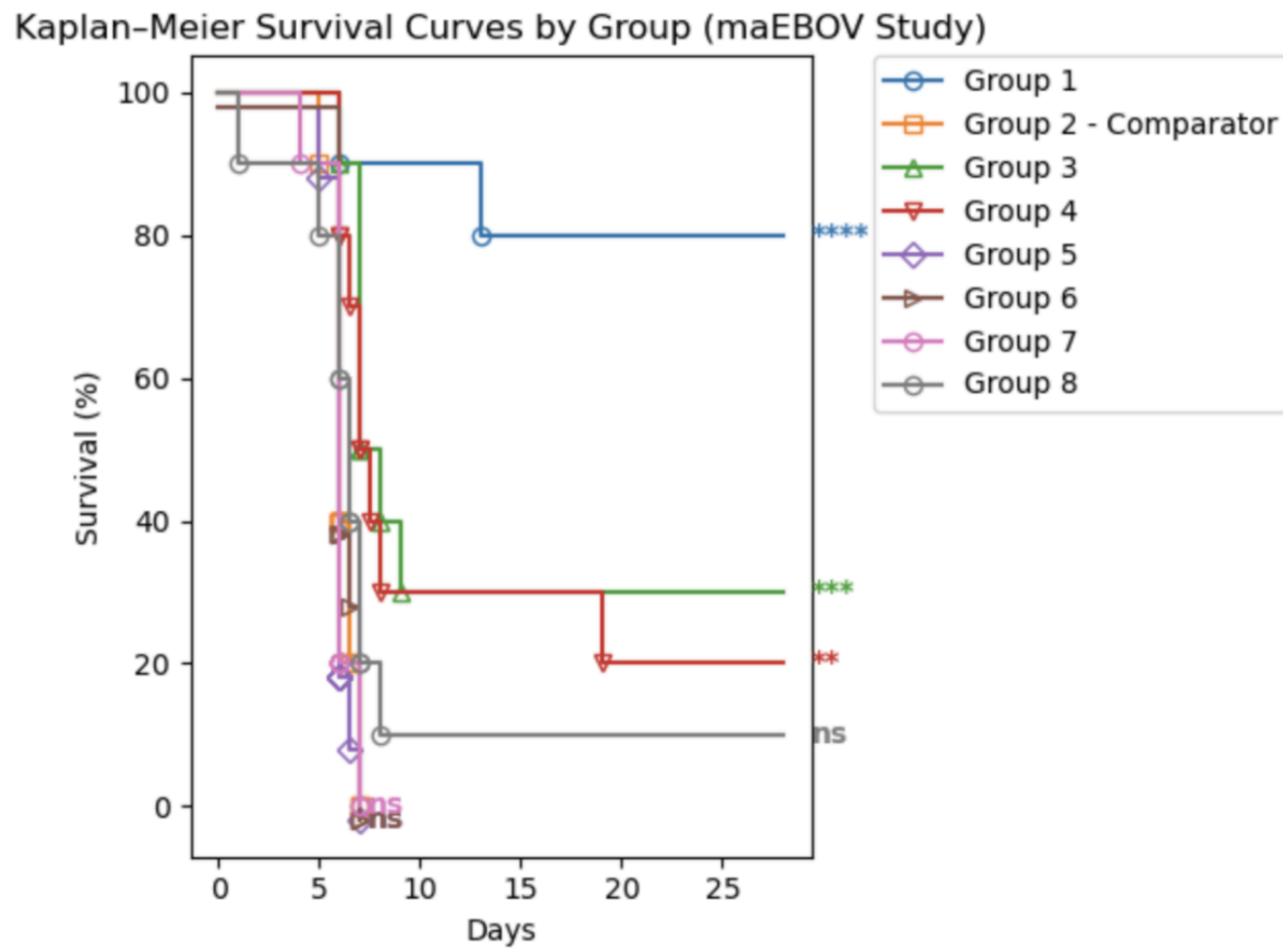

b

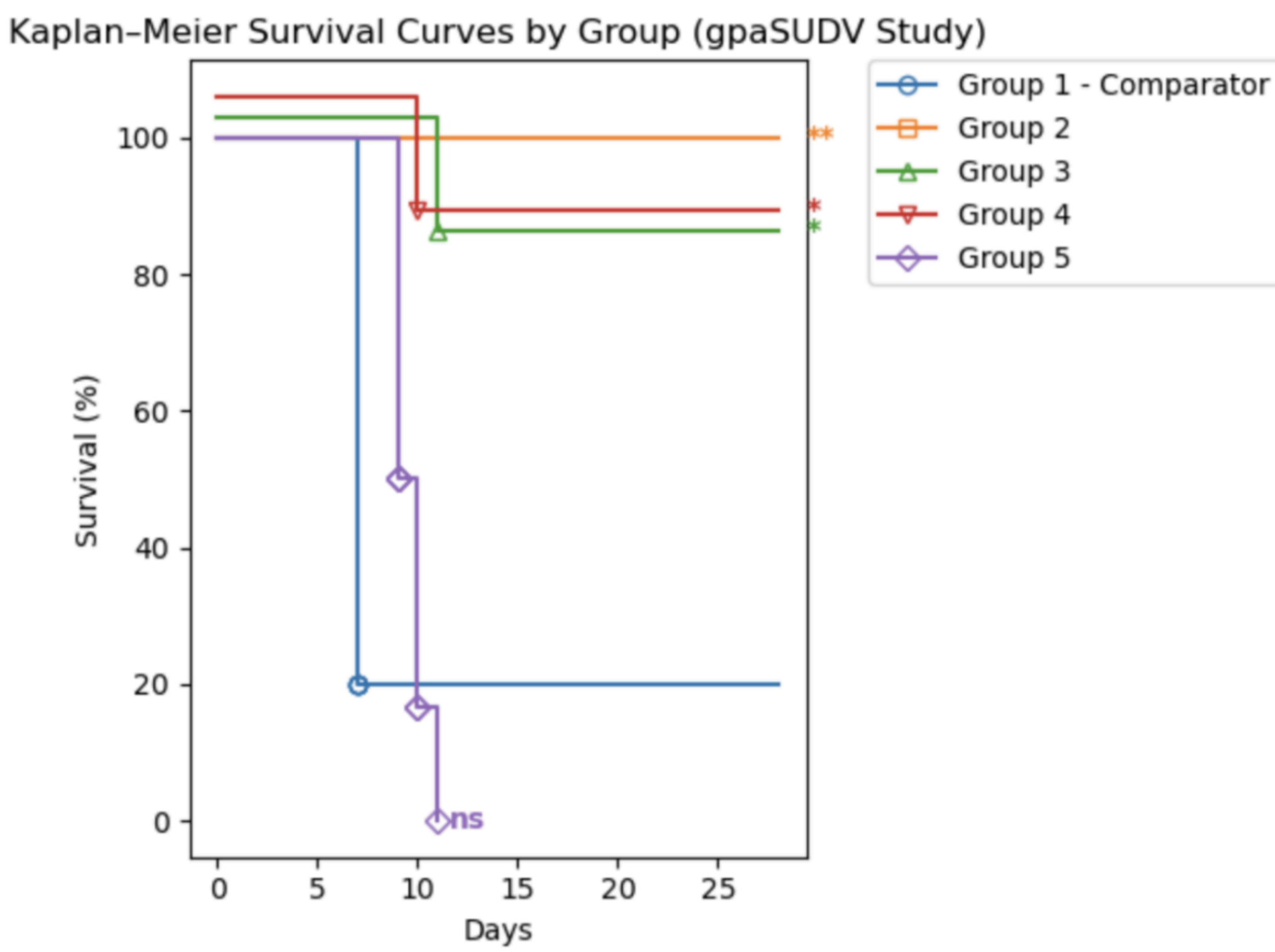

Supplemental Figure 23. Statistical Analysis of Animal Survival for EBOV/SUDV GP-I53-50 NP Mosaic and Cocktail Immunogens in Comparison to SUDV-GP-I53-50 (in maEBOV study) or EBOV-GP-I53-50 (in gpaSUDV study). a. Survival data from the maEBOV study (Figure 4) where we compared EBOV-GP-I53-50 (Group 1), mosaic (Group 3), or cocktail (Group 4) survival data to that of SUDV-I53-50 NP (Group 2 - Comparator) using the on log-rank Mantel-Cox statistical analysis. **b.** Survival data from the gpaEBOV study (Figure 5) where we compared SUDV-GP-I53-50 (Group 2), mosaic (Group 3), or cocktail (Group 4) survival data to that EBOV-I53-50 NP (Group 1 - Comparator) using the on log-rank Mantel-Cox statistical analysis. The level of statistical significance is indicated as: *P < 0.05; **P < 0.01; ***P < 0.001. See source data figures 4 and 5 for N values.

Protein Name:	EBOV-GP-I53-50 NP
Lot Number:	20210715
Date of Manufacture:	20210715
Final Formulation:	50 mM Tris pH 8.0, 150 mM NaCl, 100 mM Arginine, 5% Glycerol
**Spin Recommendation:**	**5 minutes, 14,000 rcf**
Storage Condition:	-80°C

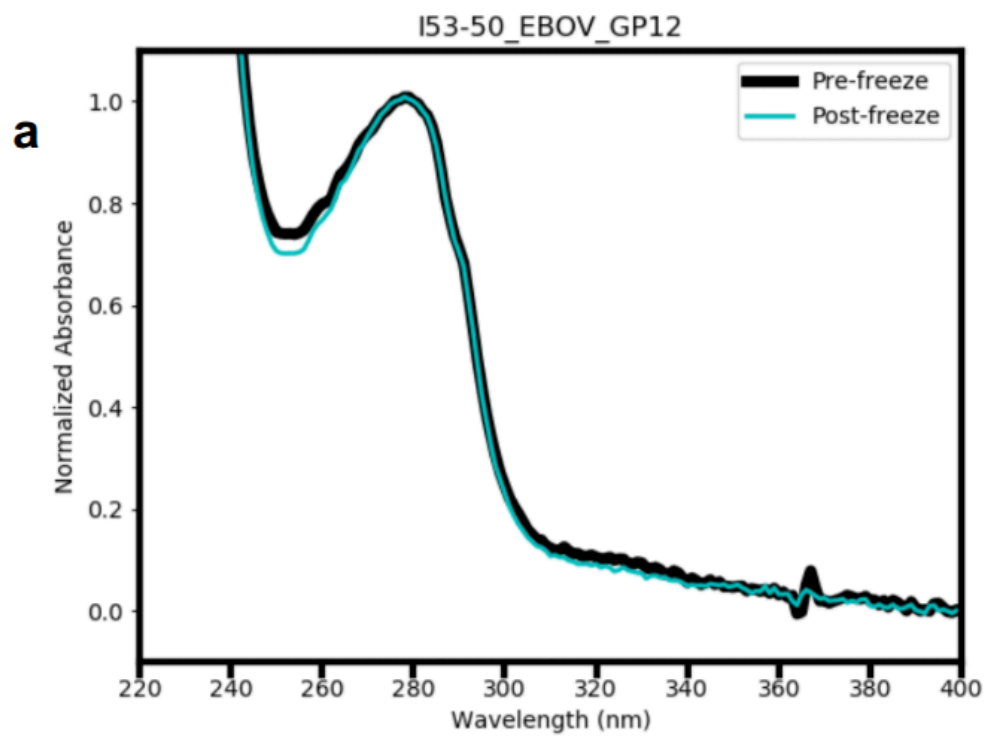

Test	Result
Appearance	Clear solution, with no particles present
Protein Concentration (A_{280})	3.16 μM (0.307 mg/mL)<br>Endotoxin: 0.14 EU/dose<br>Extinction Coefficient: 98306
Expected Molecular Weight	97123.58 Da
SDS-PAGE, Reducing/Non-Reducing	Bands observed around expected molecular weights
UV-Vis	Peak observed at 280 nm with minimal scattering at 320 nm
DLS	Peak observed at expected diameter with some aggregation
EM	Some clumping observed post-freeze. Cleared up by post freeze centrifugation.

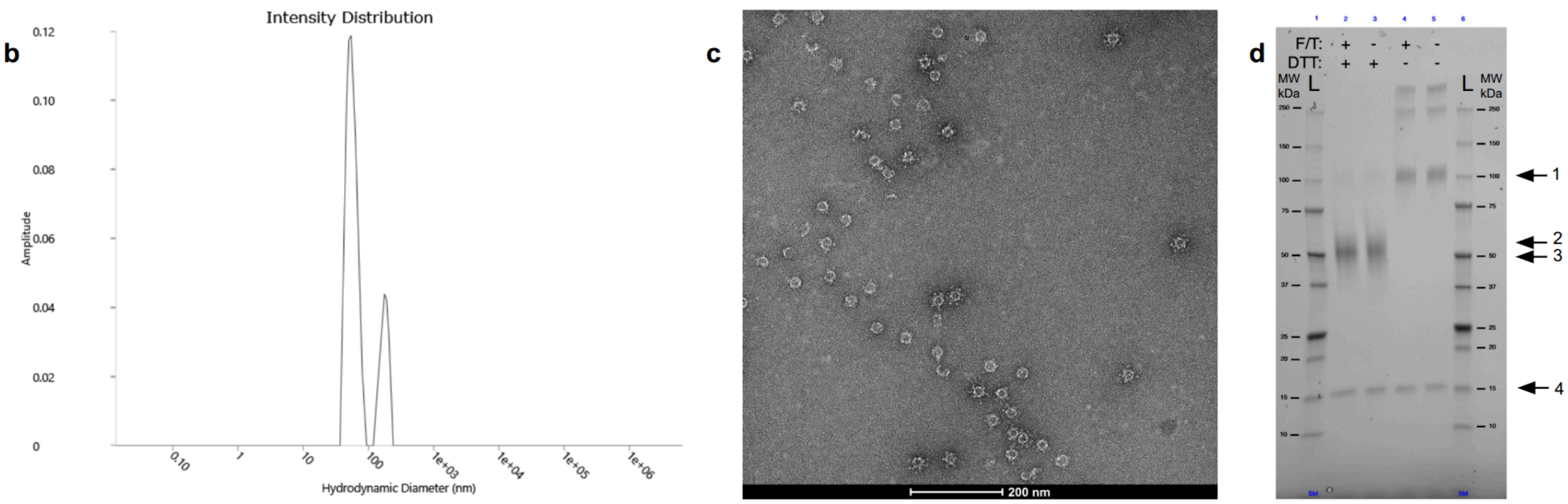

Supplemental Figure 24. EBOV-GP-I53-50 Nanoparticle Immunogens were prepared for the gpaSUDV study in July of 2021 by mixing purified I53-5B.4PT1 protein with IMAC-SEC purified EBOV-GP-I53-50A MDT-000868 produced by pCMV/R transient transfection using ExpiHEK293F cells, followed by SEC purification of the assembled EBOV-GP-I53-50 nanoparticles in storage buffer (50 mM Tris pH 8.0, 150 mM NaCl, 100 mM Arginine, 5% glycerol). **Top tables.** Certificate of testing summary information for immunogen material used in animal immunizations is noted in tabular form above figure panels. **a.** The EBOV-GP-I53-50 NP material was characterized by UV-Visible wavelength absorbance scan before (black line) and after freeze-thaw (cyan line). **b.** The post freeze thaw nanoparticles were centrifuged at 4°C for 5 minutes at 14,000xg and then characterized by dynamic light scattering (DLS) in triplicate. Trace amounts of aggregated NPs were observed with hydrodynamic radius above 100 nM; an example intensity distribution is shown. **c.** The post freeze thaw, post centrifugation (4°C for 5 minutes at 14,000xg) nanoparticles were subject to negative stain EM analysis and the majority of observed material were single particle NPs (as expected based on DLS and UV-Vis scan). **d.** The SEC purified nanoparticles were analyzed by SDS-PAGE (Criterion 4-20% Tris-Glycine TGX gel StainFree, 300V for 20 minutes) under reducing (DDT +) and non-reducing (DTT -)) conditions for material pre- (F/T -) or post (F/T +) freeze thaw (1.5 μg load). With ladder (L) in lanes 1 & 6 with molecular weight (MW) markers in kilodaltons (kDa). Arrows indicate expected mass locations for band 1: GP1-(ΔMucin)-GP2-(ΔMPER-TM)-I53-50A (lanes 4 & 5); Band 2: GP1-(ΔMucin) (lane 2 & 3); Band 3: GP2-(ΔMPER-TM)-I53-50A (lane 2 & 3); Band 4. I53-50B (lanes 2-5).

Protein Name:	SUDV-GP-I53-50 NP
Lot Number:	20210715
Date of Manufacture:	20210715
Final Formulation:	50 mM Tris pH 8.0, 150 mM NaCl, 100 mM Arginine, 5% Glycerol
**Spin Recommendation:**	**5 minutes, 14,000 rcf**
Storage Condition:	-80°C

Test	Result
Appearance	Clear solution, with no particles present
Protein Concentration (A_{280})	3.40 µM (0.332 mg/mL)<br>Endotoxin: 0.13 EU/dose<br>Extinction Coefficient: 101286
Expected Molecular Weight	97681.12 Da
SDS-PAGE, Reducing/Non-Reducing	Bands observed around expected molecular weights
UV-Vis	Peak observed at 280 nm with minimal scattering at 320 nm
DLS	Peak observed at expected diameter
EM	Some clumping observed post-freeze. Cleared up by post freeze centrifugation.

a

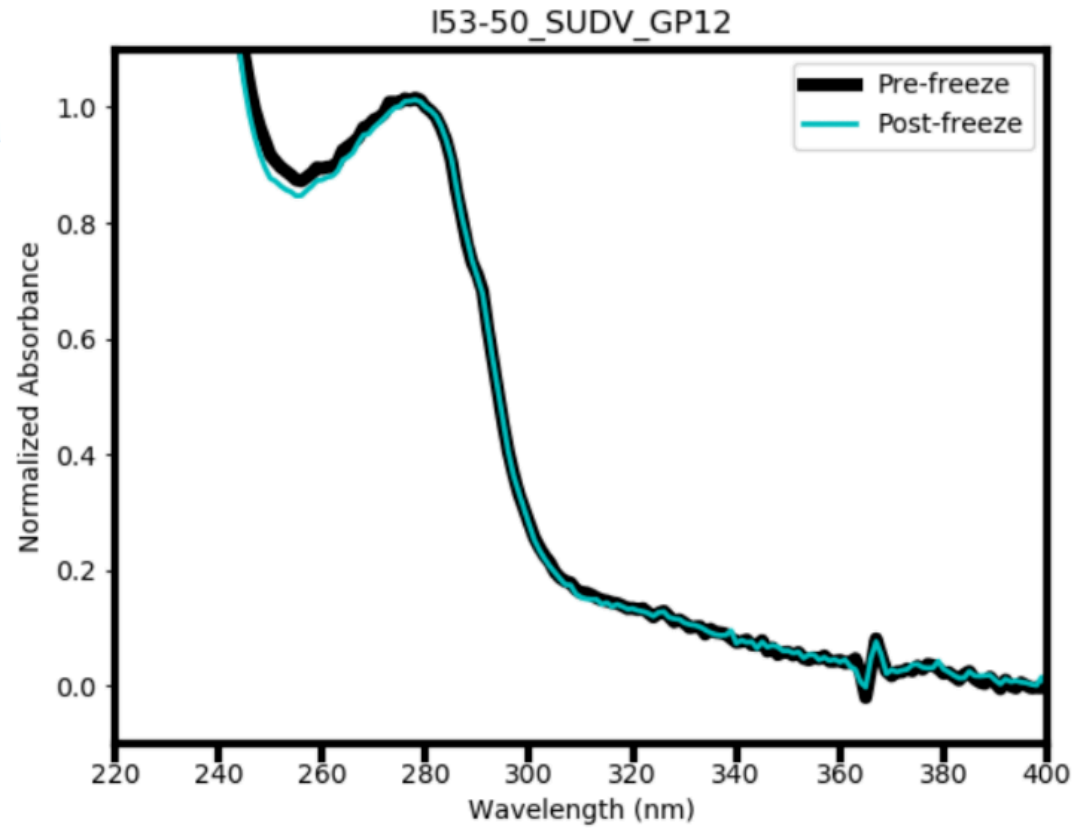

b

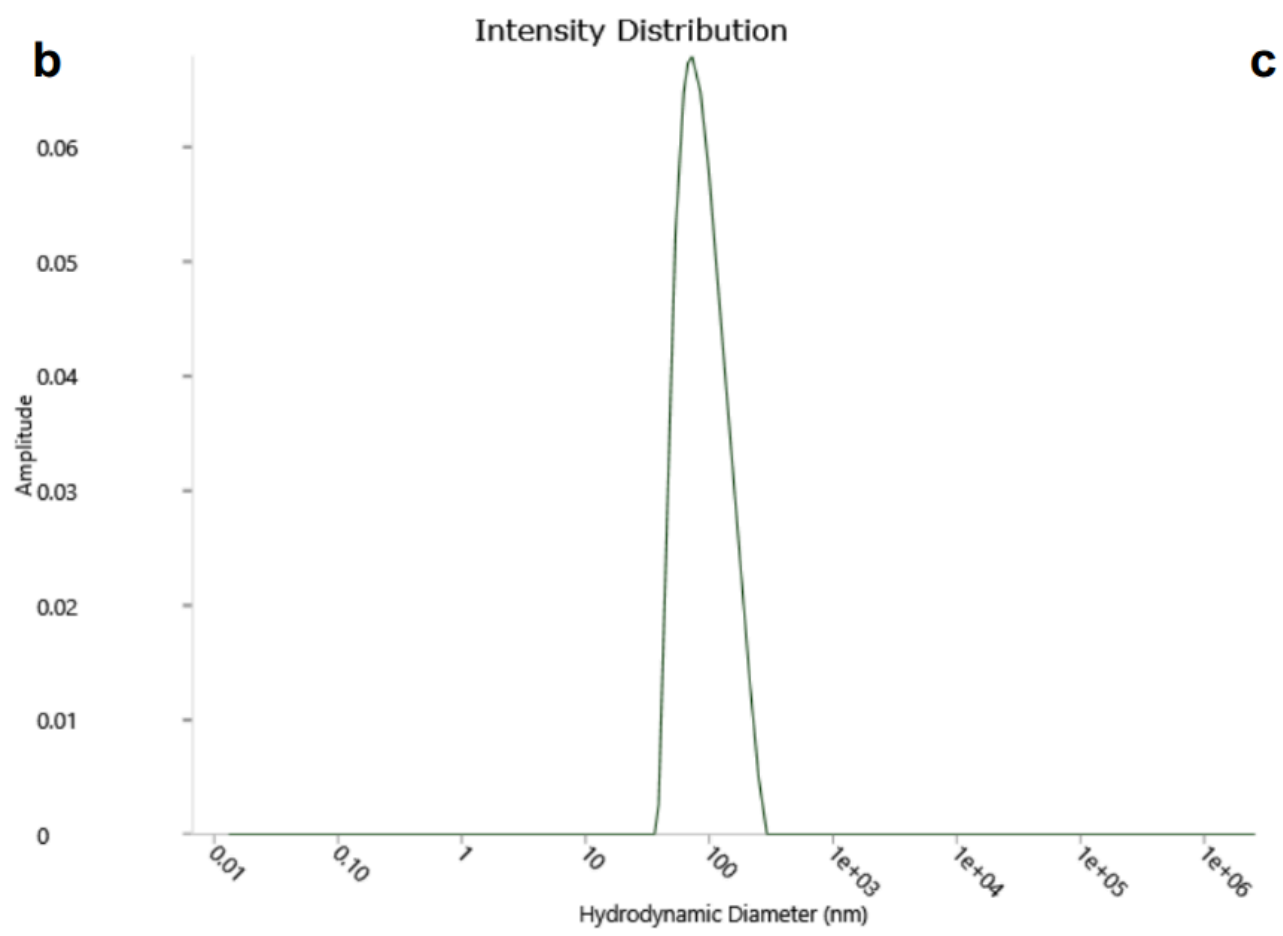

c

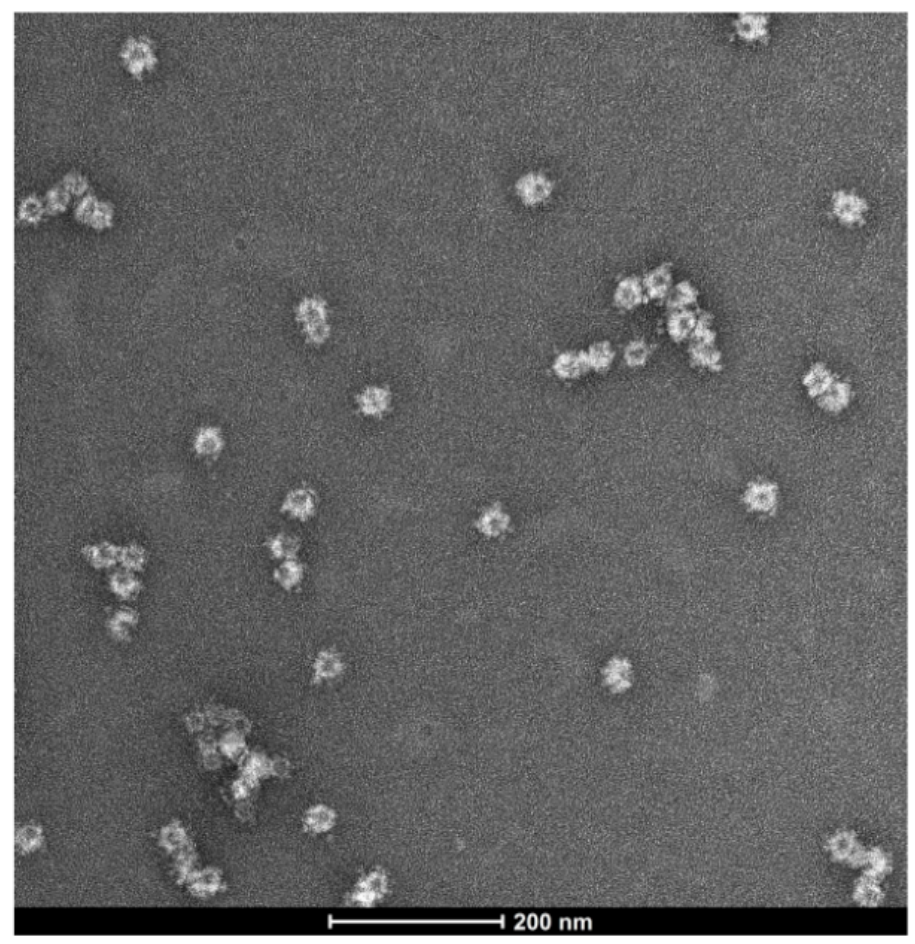

d

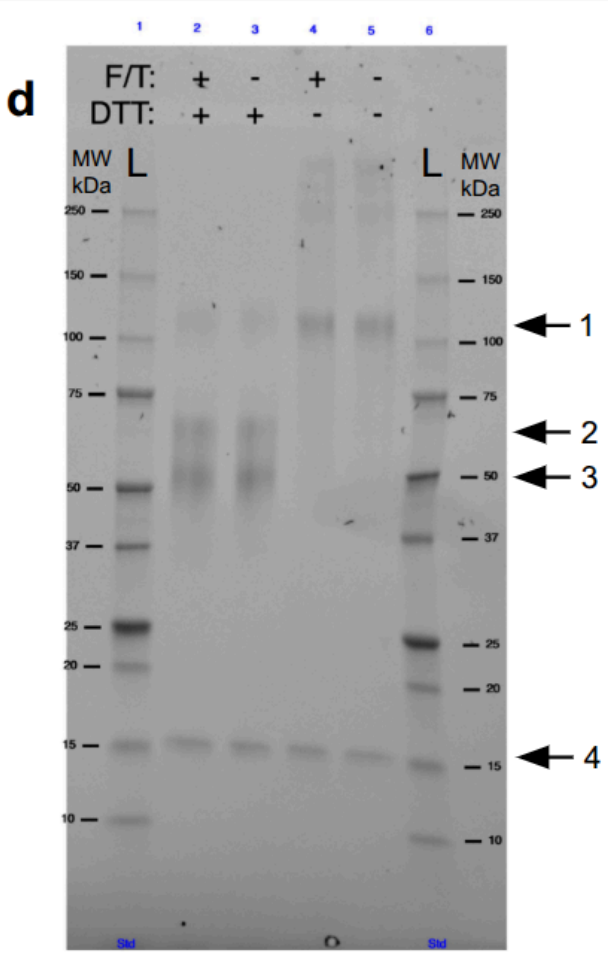

Supplemental Figure 25. SUDV-GP-I53-50 Nanoparticle Immunogens were prepared for the gpaSUDV study in July of 2021 by mixing purified I53-5B.4PT1 protein with IMAC-SEC purified SUDV-GP-I53-50A MDT-000869 produced by pCMV/R transient transfection using ExpiHEK293F cells, followed by SEC purification of the assembled SUDV-GP-I53-50 nanoparticles in storage buffer (50 mM Tris pH 8.0, 150 mM NaCl, 100 mM Arginine, 5% glycerol). **Top tables.** Certificate of testing summary information for immunogen material used in animal immunizations is noted in tabular form above figure panels. **a.** The SUDV-GP-I53-50 NP material was characterized by UV-Visible wavelength absorbance scan before (black line) and after freeze-thaw (cyan line). **b.** The post freeze thaw nanoparticles were centrifuged at 4°C for 5 minutes at 14,000xg) and then characterized by dynamic light scattering (DLS) in triplicate. Trace amounts of aggregated NPs were observed with hydrodynamic radius above 100 nM; an example intensity distribution is shown. **c.** The post freeze thaw, post centrifugation (4°C for 5 minutes at 14,000xg) nanoparticles were subject to negative stain EM analysis and the majority of observed material were single particle NPs along with two, three or four particle clusters (as expected based on DLS and UV-Vis scan). **d.** The SEC purified nanoparticles were analyzed by SDS-PAGE (Criterion 4-20% Tris-Glycine TGX gel StainFree, 300V for 20 minutes) under reducing (DDT +) and non-reducing (DTT -) conditions for material pre- (F/T -) or post (F/T +) freeze thaw (1.5 µg load), with ladder (L) in lanes 1 & 6 with molecular weight (MW) markers in kilodaltons (kDa). Arrows indicate expected mass locations for Band 1: GP1-(ΔMucin)-GP2-(ΔMPER-TM)-I53-50A (lane 4 & 5); Band 2: GP1-(ΔMucin) (lane 2 & 3); Band 3: GP2-(ΔMPER-TM)-I53-50A (lane 2 & 3); Band 4. I53-50B (lanes 2-5).

Protein Name:	Mosaic EBOV/SUDV-GP-I53-50 NP
Lot Number:	20210813
Date of Manufacture:	20210813
Final Formulation:	50 mM Tris pH 8.0, 150 mM NaCl, 100 mM Arginine, 5% Glycerol
Storage Condition:	-80°C

Test	Result
Appearance	Clear solution, with no particles present
Protein Concentration (A_{280})	1.48 µM (0.144 mg/mL)<br>Endotoxin: 10.3 EU/dose<br>Extinction Coefficient: 99796
Expected Molecular Weight	97541.73 Da
SDS-PAGE, Reducing/Non-Reducing	Bands observed around expected molecular weights
UV-Vis	Peak observed at 280 nm with minimal scattering at 320 nm
DLS	Peak observed at expected diameter with no aggregation
EM	Well-formed particles with some aggregate observed.

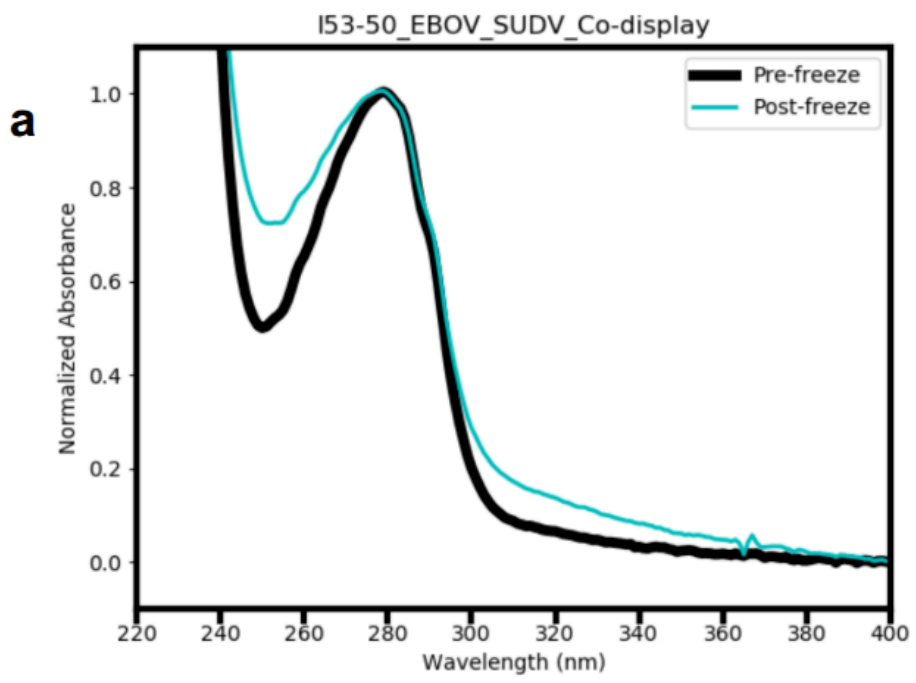

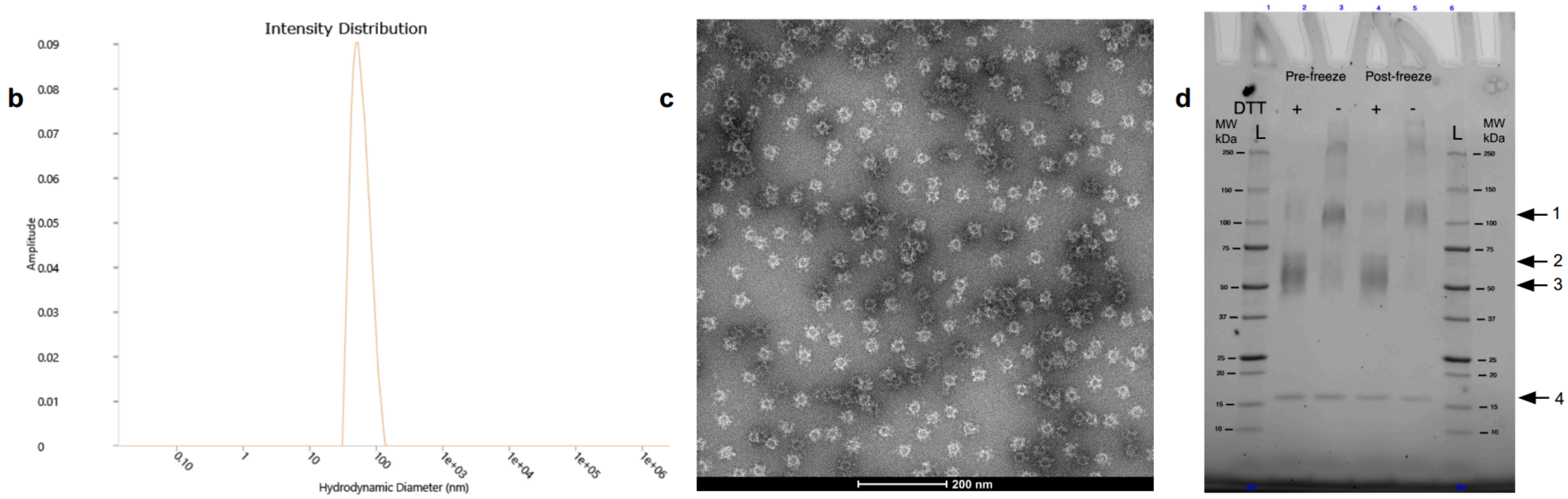

Supplemental Figure 26. Mosaic EBOV/SUDV-GP-I53-50 Nanoparticle Immunogens were prepared for the gpaSUDV study in July of 2021 by mixing purified I53-5B.4PT1 protein with IMAC-SEC purified EBOV-GP-I53-50A MDT-000868 and SUDV-GP-I53-50A MDT-000869 both produced by pCMV/R transient transfection using ExpiHEK293F cells, followed by SEC purification of the assembled EBOV/SUDV-GP-I53-50 nanoparticles in storage buffer (50 mM Tris pH 8.0, 150 mM NaCl, 100 mM Arginine, 5% glycerol). Top tables: Certificate of testing summary information for immunogen material used in animal immunizations is noted in tabular form above figure panels. **a.** The Mosaic EBOV/SUDV-GP-I53-50 NP material was characterized by UV-Visible wavelength absorbance scan before (black line) and after freeze-thaw (cyan line). **b.** The post freeze thaw nanoparticles were centrifuged at 4°C for 5 minutes at 14,000xg) and then characterized by dynamic light scattering (DLS) in triplicate. Trace amounts of aggregated NPs were observed with hydrodynamic radius above 100 nM; an example intensity distribution is shown. **c.** The post freeze thaw, post centrifugation (4°C for 5 minutes at 14,000xg) nanoparticles were subject to negative stain EM analysis and the majority of observed material were single particle NPs along with two, three or four particle clusters (as expected based on DLS and UV-Vis scan). **d.** The SEC purified nanoparticles were analyzed by SDS-PAGE (Criterion 4-20% Tris-Glycine TGX gel StainFree, 300V for 20 minutes) gel under reducing (DTT +) and non-reducing (DTT -) conditions for material pre-freeze or post-freeze thaw (1.5 µg load). With ladder (L) in lanes 1 & 6 with molecular weight (MW) markers in kilodaltons (kDa). Arrows indicate expected mass locations for Band 1: GP1-(ΔMucin)-GP2-(ΔMPER-TM)-I53-50A (lane 3 & 5); Band 2: GP1-(ΔMucin) (lane 2 & 4); Band 3: GP2-(ΔMPER-TM)-I53-50A (lane 2 & 4); Band 4. I53-50B (lanes 2-5).

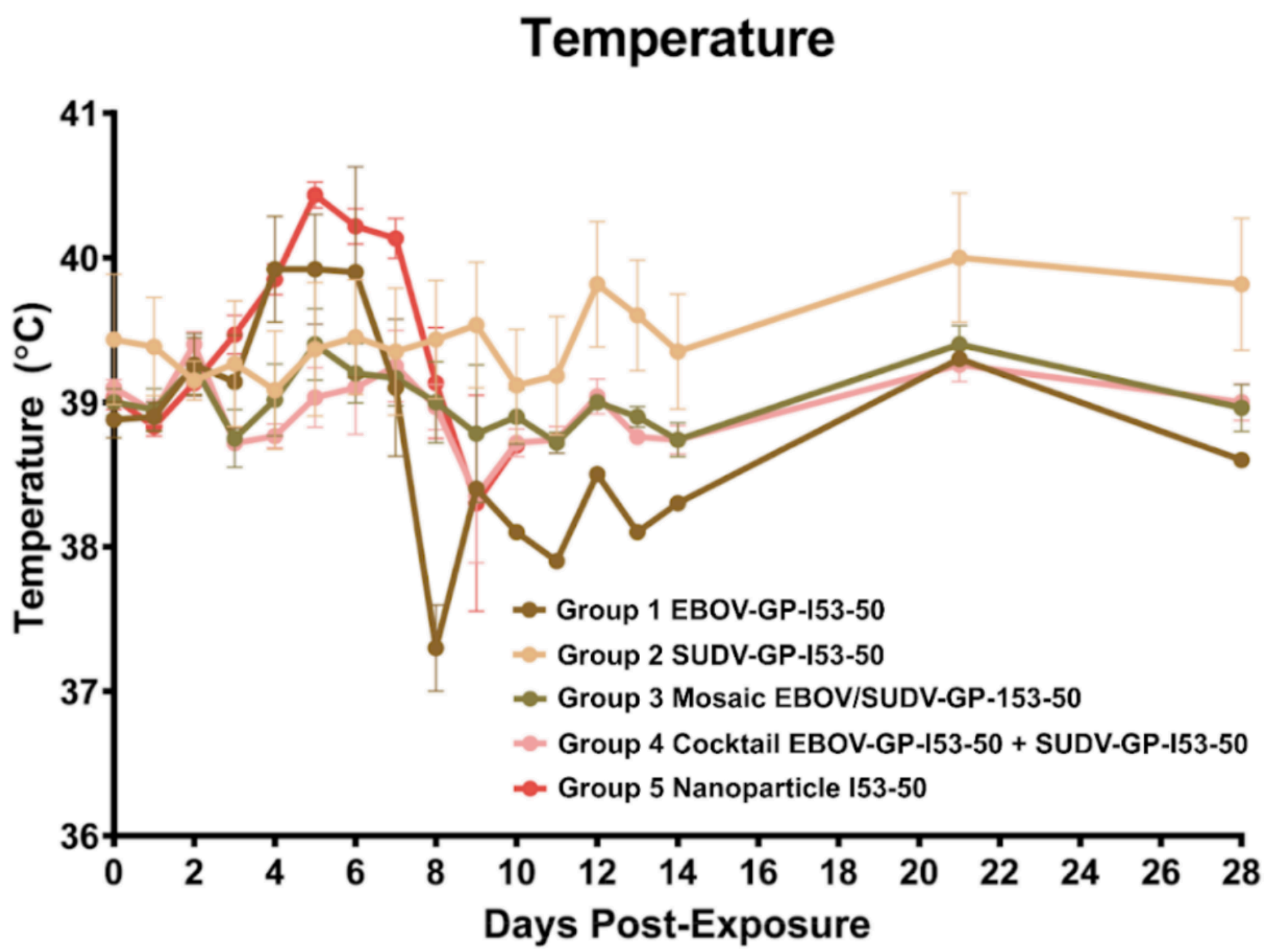

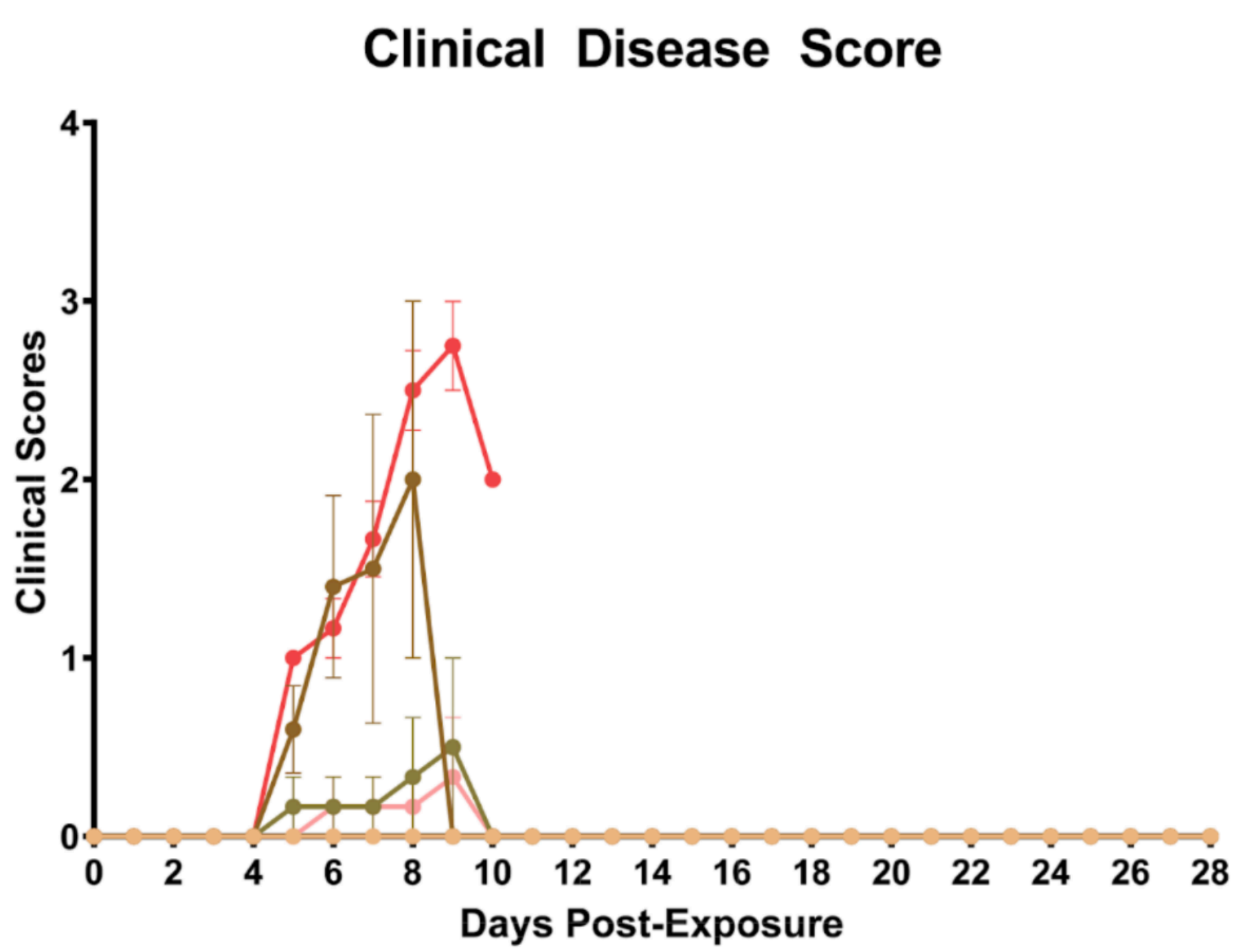

Supplemental Figure 27. Temperature and Clinical Disease Scores for Prophylactic Immunization and Challenge with the Guinea Pig Adapted SUDV. (Top panel) Daily temperature data (by subcutaneous chip; BDMS) for animals recorded for 28 days in the gpaSUDV study. Mean values with +/- SEM and N sample size see table below. Moribund guinea pigs were promptly euthanized when they met predefined euthanasia criteria, a score of 4; defined as bleeding, unresponsive, weak, or unable to walk. Legend for Groups is indicated in the upper right. Full clinical disease score is as follows: (Bottom panel) Post-exposure, mice were observed daily for weight and clinical signs of disease for up to 28 days. Upon onset of clinical signs of disease, mice were observed at least twice daily. Mean values with +/- SD and N sample size in the source data file (Figure 5). Moribund mice, with maximum clinical disease scores of 4 (defined as bleeding, unresponsive, weak, unable to walk) or surviving through day 28 post exposure were euthanized. The full scoring/euthanasia criteria was as follows: 0 = normal (smooth coat; eyes/nose clear); 1 = reduced grooming/ruffled fur; 2 = subdued response, but normal when stimulated; 3 = lethargic; hunched posture; subdued response, even when stimulated; 4 = (euthanize) unresponsive when provoked, weak, unable to walk.

Supplementary Table S1. CryoEM Data Collection Statistics: EBOV-GP MDT-000759

**Data Collection**	
Microscope	Titan Krios
Voltage (kV)	300
Detector	Gatan K3
Energy Filter	Gatan<br>BioQuantum Gif
Recording mode	Counting
Magnification	105,000 X
Movie micrograph pixel size (Å)	0.84
Dose rate (e^{-}/Å^2/s)	11.34
No. of frames per movie micrograph	100
Frame exposure time (s)	0.05
Movie micrograph exposure time (s)	5.0
Total dose (e^{-}/Å^2)	56.69
Under focus range (μm)	0.8 - 1.8
Number of movie micrographs	3,128
**Map Processing**	
Extraction Box Size (pix)	256
Initial particle images (no.)	540,665
Final particle images (no.)	189,700
Map resolution (Å)	3.05
FCS threshold	0.143
Map resolution range (Å)	2.79 - 3.24
**Refinement**	
Initial model used	5JQ3
PDB ID and EMDB ID	9MHA, EMD-48271
Map resolution (Å)	3.05
FCS threshold	0.143
Model resolution range (Å)	2.79 - 3.24
Map sharpening B factor	DeepEMhancer
Model composition	
Non-hydrogen atoms	6194
Protein Residues	810
Ligands	BMA: 3, NAG: 6
B factors (Å)	
Protein	DeepEMhancer
Ligands	DeepEMhancer
R.M.S. deviations	
Bond lengths (Å)	0.004
Bond angles (°)	0.589
Validation	
MolProbity score	1.12
Clashscore	1.82
Rotamer Outliers (%)	0.34
Ramachandran plot	
Favored (%)	96.97
Allowed (%)	3.03
Disallowed (%)	0.00

ORCID

Connor Weidle ORCID: 0000-0002-9354-2276
Natalie Brunette ORCID: 0000-0002-0558-5584
Samuel P Wrenn ORCID: 0000-0002-4280-1012
Brooke Fiala ORCID: 0000-0003-0148-5268
Rashmi Ravichandran ORCID: 0000-0002-2630-7487
Kenneth D. Carr ORCID: 0009-0005-1929-3952
Samantha E Zak ORCID: 0009-0002-0368-0139
Elizabeth E Zumbrun ORCID: 0000-0002-9897-7201
Russell R Bakken ORCID: none
Michael Murphy ORCID: 0000-0002-8438-2653
Zhitong Peng ORCID: 0009-0007-3968-3637
Sidney Chan ORCID: 0000-0002-1617-0464
Rebecca Skotheim ORCID: 0009-0005-0757-8432
Andrew J Borst ORCID: 0000-0003-4297-7824
Lauren Carter ORCID: 0000-0002-9837-9068
Colin E Correnti ORCID: 0000-0003-3710-9619
John M Dye ORCID: 0000-0002-0883-5016
David Baker ORCID: 0000-0001-7896-6217
Neil P King ORCID: 0000-0002-2978-4692
Lance J Stewart ORCID: 0000-0003-4264-5125

116. UniProt Consortium. UniProt: The universal protein knowledgebase in 2023. *Nucleic Acids Res.* **51**, D523–D531 (2023).

117. Gire, S. K. *et al.* Genomic surveillance elucidates Ebola virus origin and transmission during the 2014 outbreak. *Science* **345**, 1369–1372 (2014).

118. Brouwer, P. J. M. *et al.* Lassa virus glycoprotein nanoparticles elicit neutralizing antibody responses and protection. *Cell Host Microbe* **30**, 1759–1772.e12 (2022).
