# Supplementary material for: Pan-Ebolavirus nanoparticle vaccine provides protection in rodents from lethal infection by Zaire and Sudan viruses
Source: Nat Commun. 2026 Aug 8;17:9541. doi: 10.1038/s41467-026-76114-1 (PMC13547272; doi:10.1038/s41467-026-76114-1)
Supplement: Supplementary file 2 [file 41467_2026_76114_MOESM2_ESM.pdf]

Description of the Additional Supplementary Files

Supplementary Data 1: Amino acid sequence information for all constructs
