# Supplementary material for: Pan-Ebolavirus nanoparticle vaccine provides protection in rodents from lethal infection by Zaire and Sudan viruses
Source: Nat Commun. 2026 Aug 8;17:9541. doi: 10.1038/s41467-026-76114-1 (PMC13547272; doi:10.1038/s41467-026-76114-1)
Supplement: Supplementary file 4 [file 41467_2026_76114_MOESM5_ESM.pdf]

nature portfolio

# Peer Review File

# Pan-Ebolavirus Nanoparticle Vaccine Provides Protection in Rodents from Lethal Infection by Zaire and Sudan Viruses

Corresponding Author: Dr Lance Stewart

**This file contains all reviewer reports in order by version, followed by all author rebuttals in order by version.**

Version 0:

Reviewer comments:

Reviewer #1

(Remarks to the Author)
This manuscript is written very well with only minor suggested corrections of the text. A vast amount of supplementary data is presented in addition to high quality main figures describing research efforts towards a bivalent vaccine encompassing EBOV and SUDV components.

While the overall efforts are commendable and lead towards the potential development of interesting vaccine candidates, the present stage data do not support the conclusions, especially in the context of existing published research by other groups.

For example in line 51, three references are listed of vaccine candidates with proof of efficacy in NHP's. Unfortunately not a single one showing SUDV efficacy is listed. This may mislead a less versed reader as to the current state of the field. Of particular relevance to the platform presented here, efforts related to adjuvanted protein vaccines would be necessary.
Further incomplete data presentation includes the efficacy data from the Ebola ca suffit study (line 59). Here a final study report with efficacy results has been published (in 2016).
In line 103 two references cite efforts on protein subunit vaccines agains EBOV, unfortunately this is not comprehensive and is missing highly relevant candidates that would later help to position the data in the appropriate context for discussion.
For the animal study design, many important aspects are missing that would be highly relevant in terms of reproducibility. The authors fail to describe why the subcutaneous route was chosen for both mouse and guinea pig studies, but also what exact (GP) antigen dose was given to mice. For both animal species injection volumes were also omitted.
It has to be noted that the efficacy levels against EBOV in the mouse model are not truly impressive, especially in the context of other vaccine candidates and also the SUDV efficacy in the guinea pig model is not much stronger. The data indicates some early achievenments, however, rodent models of filovirus infection are not very predictive of efficacy in (relevant) primate models.
In the discussion the rather long section about passive immunotherapies should (in line 308) more clearly describe that ZMapp protects against EBOV and then the latter mAb cocktails against SUDV are still under development.
The section discussing structural findings is overall fine, however, the prefusion trimeric EBOV-GP that was analyzed was a truncated version as described in detail. The absence of larger sections such as the mucin-like domain could very well have further implications in the accuracy of the modeled protein structure.
Lines 343-344 state that virus neutralizing titers could not be obtained due to BSL-4 requirements, however there are several BSL2-level surrogate assays (based on rVSV or lentiviruses) available that could have easily been used and would have strengthened the dataset, in particular as a potential readout to be correlated with survival in the rodent models.
In the further hypothetical discussion of what all could be included (even though the current candidate does not meet the mark yet), Marburg virus is implied to also be an Ebolavirus (line 349). And the next line speaks only about EVD-causing filoviruses (what about MVD?).
The entire paragraph lines 358-370 sounds like positioning a technology for a grant or contract, but at the current stage of development, I don't think that has a place in the current manuscript.
I also think lines 378-381 are highly speculative as at this point it is hard to predict whether primates will be protected with the nanoparticle vaccine in its current form, so discussing a potential boost in people is extremely speculative as an ineffective booster could cause more harm than do good.
In M&M I noticed some things...
section 549-567 is missing a lot of details on vendors, line 553 misspelling of "Whatman"

missing volumes/doses in both animal studies
line 601: after giving excellent detail on the vaccine antigens, some more information on the coating antigens for ELISA are needed to replicate and position immunogenicity results. Also no description is given on what dilutions were analyzed on the ELISA plates.
Figure 5 (legend) line 714 speaks of mice when it should read guinea pigs...
Supplemental figure 2 - the text speaks of I53-50A, the figure itself (line 829) says "153"

Reviewer #2

(Remarks to the Author)
Review of manuscript# NCOMMS-25-02639

In this manuscript, Brunette et al. developed and characterized a series of protein nanoparticle vaccines against two species of the ebolavirus genus, EBOV and SUDV. The authors displayed mucin-like domain (MLD)-deleted glycoprotein (GP Muc) trimers on the I53-50 2-component nanoparticle (2c-NP) platform, determined their atomic-level structures by cryo-electron microscopy (cryo-EM), and carried out challenge studies in mice and guinea pigs to demonstrate protection conferred by either cocktail or mosaic vaccines. This work was highly similar to previous studies done by the same group of authors: they utilized the I53-50 2c-NP platform to display either wildtype viral antigens or rationally designed viral antigens licensed from other institutions as vaccine candidates, with supporting data generated through structural analyses and in vivo assessment. There is no doubt about the importance of a broadly protective ebolavirus vaccine, and the logic and rationale of the study are clear. The animal studies were well-designed; we were particularly impressed by the inclusion of matched “non-assembling” subunit controls as well as bare I53-50 2c-NPs. However, their vaccine construct design is outdated and could be significantly improved by incorporating recent advances in rational GP design and stabilization. Given the suboptimal GP design, we have concerns about the stability of the EBOV, SUDV, and mosaic NP constructs and would like to see data addressing this. More concerningly, the authors’ Discussion of their own data is selective and, in some cases, misleading in what appears to be a deliberate attempt to promote mosaic and/or 2c-NP vaccines with little supporting data. I suggest that the authors address these issues with rigorous experimental data and revise the writing.

Major comment #1:
This study was built upon the hypothesis that a cocktail or a mosaic vaccine containing both EBOV and SUDV components will protect against these two ebolavirus species more effectively than a vaccine based on a single ebolavirus GP backbone. This hypothesis is inconsistent with a large body of antibody work published over the last decade, with panels of potent pan-ebolavirus neutralizing antibodies (nAbs) isolated from human survivors (e.g., ADI-15878 and ADI-15946) or immunized nonhuman primates (e.g., CA45). The elicitation of pan-ebolavirus nAbs during infection and vaccination provides strong evidence for the development of broadly protective ebolavirus vaccines based on a single ebolavirus GP backbone.

Therefore, the lack of cross-ebolavirus antibody response observed in this study, as evidenced by lines 221-223 “Little or no cross-reactive antibody responses were observed: only 3 out of 10 animals that received EBOV-GP-I53-50 had any observable anti-SUDV-GP antibodies, while none of the ten animals that received SUDV-GP-I53-50 had any appreciable anti-EBOV-GP antibodies,” was very concerning, suggesting potential problems with vaccine construct design. In fact, the He et al. study in 2021 showed that EBOV GP-NP-induced sera were cross-reactive and robustly neutralized pseudotyped BDBV, another ebolavirus species.

The statement in the next sentence “This was expected as the EBOV and SUDV GP components of our NP immunogens have only 68% sequence identity (Supplemental 225 Figures 18 and 19), and previous reports on filovirus vaccine candidates have suggested that little heterologous cross-protection can be obtained with single filoviral GP immunogens.” is purposedly confusing. As leading experts in protein structure prediction, the authors must know that 68% sequence identity means high homology in protein structure and function, and furthermore, “filovirus” is different from “ebolavirus” in scope, as the former contains several diverse viral families and the latter has 6 species including EBOV and BDBV, which are closely related.

Similar misleading statements can be found throughout the manuscript.

Major comment #2:
The vaccine constructs used in this study (lines 140-153), appeared to not have considered recent advances in rational GP design and stabilization (Rutten et al., 2020, Cell Rep 30, 4540–4550; He et al., 2021, Nat Commun 12, 2633). These constructs only included basic modifications that were used in early studies to produce soluble GPs, such as removal of the membrane-proximal external region (MPER) and MLD, as well as the T42A mutation. None of the critical stabilizing mutations reported in the Rutten and He et al. papers, such as the proline mutations in HR1c and various mutations in HR2, which substantially improved GP trimer formation and stability, was tested in this study. Due to the use of a less table, nearly wildtype GP, the resulting EBOV-GP-foldon and SUDV-GP-foldon constructs produced trimeric, dimeric, and monomeric GP species, as indicated by the SEC profile after IMAC purification (Figure 1C). The GP trimer stability problem became worse when it was directly attached to the I53-50A trimer without a foldon motif, as indicated by the SEC profile (Supplemental Figure 2). Even though the trimer fraction can be extracted through repeated SEC purifications, due to the inherent GP instability this GP trimer may not remain stable when displayed on the 2c-NP surface, adjuvanted with AddaVax adjuvant, and mixed with body fluids at an elevated (body) temperature after immunization.

Structurally, I53-50 is not suitable for displaying glycoprotein trimers with a narrow stalk, e.g., filovirus GP, because its N-terminus, the trimer-anchoring site, forms a wide triangle (~50 Å). In the current EBOV/SUDV-GP-foldon-I53-50 constructs,

foldon is included to overcome the GP's inherent instability and I53-50's structural incompatibility, but itself has no biological functions. It is worth pointing out that these two problems can be dealt with using previously reported GP-stabilizing mutations and a modified I53-50 NP backbone.

The authors should determine the thermostability of their EBOV and SUDV GP trimers and I53-50 2c-NPs using differential scanning calorimetry (DSC) and include DSC profiles in revision.

Major comment #3:
In this manuscript, the authors used SKYCovione, which is a COVID-19 vaccine based on the I53-50 2c-NP, as a successful example to support the ebolavirus vaccine study based on the same platform. SKYCovione was approved for human use in South Korea and the UK but not in the US. It was reported in 2022 that in the phase 1 trial conducted in the US the vaccine group underperformed the placebo group, causing concerns about potential flaws in the 2c-NP platform. In the phase 3 trial conducted in South Korea, SKYCovione was compared with AstraZeneca's vector vaccine and showed ~2-fold increase in potency. However, AstraZeneca's vector vaccine was known to underperform mRNA vaccines by ~4-fold, suggesting that SKYCovione would seem less effective if it was compared directly with mRNA vaccines. If the authors intended to use the SKYCovione data, it would be important to provide a complete and unbiased background about the development of this vaccine, including both positive and negative data.

Major comment #4:
In lines 366-370, the authors discussed single-component and SpyTag/SpyCatcher NP systems, which were incomplete or inaccurate. First, He et al. demonstrated that both I3-01 and E2p 60-mers can be used to display stabilized EBOV GP trimers, with E2p showing greater structural compatibility with GP and eliciting more potent nAbs. However, E2p was omitted throughout the paper. Second, the statement on the inability of single-component NP to create mosaic vaccines is inaccurate. Although the 2c-NP system can readily incorporate multiple antigens by mixing different antigen-I53-50A trimers with I53-50B pentamers, the ratio and surface location of different antigens upon assembly cannot be controlled. Single-component NP systems can be used to create mosaic vaccines by mixing different antigen-NP plasmids during expression but face similar challenges with controlling the antigen ratio and surface location on the NPs. Third, given the inherent instability of the current GP and GP-foldon-I53-30 designs, the mosaic display of EBOV and SUDV GP trimers will further reduce NP stability and cause product heterogeneity. Fourth, the animal data didn't show a clear advantage of mosaic vaccines vs. cocktail vaccines.

The authors are encouraged to perform the DSC analysis of mosaic vs. cocktail I53-50 2c-NPs and compare their in vivo stabilities after immunization.

Major comment #5:
Immunogenicity data raises some concerns about the conclusions drawn from this study. First, survival data (Fig. 4f) demonstrates that EBOV-GP-I53-50 alone protects significantly better (80%) against maEBOV challenge compared to mosaic and cocktail EBOV-I53-50-GP + SUDV-GP I53-50 (20-30%), which is not consistent with the ELISA results, where these groups show similar binding against EBOV GP (Fig. 4b). This suggests that the binding antibodies elicited in mice by the cocktail and mosaic vaccines are non-neutralizing/non-Fc-mediated and thereby non-protective compared to antibodies elicited by EBOV-GP-I53-50 alone. This observation is not addressed adequately in Discussion. A key analysis that is missing here is the assessment of nAb responses elicited by these vaccines, which may better explain the discrepancy between IgG binding and survival post-challenge. An explanation to the absence of nAb data is attributed to BSL-4 facility expenses (lines 342-344). However, BSL-2-level pseudovirus assays using small blood volumes have been routinely carried out to evaluate filovirus vaccine-induced nAbs and are critical to inform conclusions for this study. Next, it is surprising that "non-assembled" counterparts to the 2c-NP vaccines (groups 5-7), which contain soluble EBOV GP and/or SUDV GP trimers, could not elicit any GP-specific binding responses nor significant protection against the maEBOV challenge. This result further brings into question the use of these EBOV GP and SUDV GP trimers as immunogens for eliciting GP-specific protective antibody responses. Lastly, it is concerning how bare I53-50 immune serum samples (from both mice and guinea pigs) were able to recognize EBOV GP- and/or SUDV GP-specific coating antigens up to nearly a 103 endpoint titer, suggesting that there might non-specific background signals in this ELISA. This is briefly mentioned (lines 232-234) but a explanation is not provided.

In addition, morbidity is defined as having lost >15% of body weight (line 238). However, how was weight loss calculated in the challenge study with the mouse adapted EBOV? Based on Fig. 4d, it appears that some mice lost over 20% of their body weight.

Major comment #6:
The authors claim that the co-display of additional Ebolavirus GPs such as those from Marburg virus (MARV), Bundibugyo virus (BDBV), or Taï Forest virus (TAFV) could broaden protection (lines 348-350). Additionally, the authors mention that mosaic 2c-NPs may be required to elicit broad spectrum responses (lines 366-368). However, as evidenced by the survival data against both maEBOV and gpaSUDV challenges, the mosaic EBOV-GP-I53-50 + SUDV-GP-I53-50 vaccine 1) always significantly underperformed the challenge-matched GP I53-50 alone and 2) never significantly outperformed its cocktail counterpart. Hence, there is minimal evidence to suggest that the development of a more complex mosaic NP incorporating additional filovirus GPs would broaden or improve the protection against other filoviruses, much less perform better than a cocktail counterpart. The authors have not adequately addressed this issue in the results or discussion of this manuscript.

Other comments:
1. Lines 199 – 200: The authors state that they produced "a mosaic I53-50 NP co-displaying EBOV- and SUDV-GP in a 1:1

ratio," but provide no evidence for this claim. While the authors mixed the EBOV and SUDV subunits in a 1:1 ratio, it does not necessarily follow that the assembled NPs will maintain this ratio, since it is entirely possible that one of the trimeric GP subunits self-assembles more efficiently with the pentameric subunit.
2. Lines 367 – 368: The authors state that their results suggest that "mosaic nanoparticles displaying oligomeric antigens… may be needed to elicit broad-spectrum immune responses." In fact, their results show that a cocktail of EBOV and SUDV NPs and mosaic NPs provide nearly identical protection from challenge. Furthermore, the cocktail elicited vaccine-specific IgG levels that were either similar to or higher than those elicited by mosaic NPs.
3. In Fig. 4B-C and Fig. 5B-C, the authors compare EBOV-GP- and SUDV-GP-specific IgG titers elicited by EBOV-GP NPs, SUDV-GP NPs, EBOV/SUDV mosaic NPs, and a cocktail of EBOV and SUBV NPs and calculate P values compared to bare NPs. Since the rationale for this study is to develop a vaccine capable of protecting against both EBOV and SUDV, it would be more informative to compare the mosaic and cocktail vaccines to the relevant single-antigen vaccine. For example, in Fig. 4C the mosaic NP elicited (significantly?) lower levels of SUDV-specific IgG than SUDV NPs, while the cocktail vaccine elicited similar levels. Similarly, in Fig. 5C the cocktail vaccine elicited EBOV-specific IgG levels similar to those of the EBOV NP, while the mosaic NP elicited much lower IgG levels which were not significantly higher than those of the negative control. This is not surprising since it is well established that the efficacy of NP vaccines is largely due to the cross-linkage of B cell receptors (BCRs) by the regular and repeated array of fixed antigens. In the context of presentation to B cells, a mosaic NP has approximately half of the antigen avidity (assuming a 1:1 ratio of antigens) as a single-antigen NP.

Minor comments:
1. The SEC profile in Figure 1c. should include the unit of X-axis and Y-axis. Especially for X-axis, the manuscript has included both FPLC and HPLC. Retention time was used in some SEC profiles (Figure 2i and 2j), but retention volume might be used in Figure 1c (?). In addition, retention time should use "minute" as the unit, not "milliliter" (Supplemental Figure 2b, 3b, 3c, 3d, 7a, 7c, etc.), which should be used as the unit for retention volume.
2. The manuscript mentioned that the HR2 stalk was not built in the cryo-EM model (line 171-172). The authors should clearly state which parts are missing in the structure.
3. The shoulder to the left of the trimer peak in the SEC profile of SUDV-GP-153-50A (MDT-000869-lenti) indicated that this protein was not homogenous, and this shoulder might correspond to aggregation or other higher molecular-weight GP forms. The authors may need to characterize the sample homogeneity with SEC-MALS.
4. The authors may want to provide more detailed description of structural detailed. The cryo-EM structure was determined at a resolution of 3.05 Å, which may not be sufficient for visualizing atomic details of the sidechains, especially when they were compared with those from a crystal structure at a resolution of 2.3 Å (Supplemental Figure 6). Sidechain orientation in the cryo-EM structure may not be as accurate as in the crystal structure
5. In Figure 2, the authors showed representative 2D classifications and 3D density models derived from the negative-stain EM analysis for EBOV-GP-I53-50A and SUDV-GP-I53-50A, respectively. Both I53-50A-attached ebolavirus GP trimers appeared to exhibit C3 symmetry, judged by the 2D classification images. However, the trimeric GP showed a ring-like shape instead of C3 symmetry in the 3D models. The authors should include more technical details for their negative-stain EM analysis (e.g., 2D classification and 3D modeling) and provide some explanations for the non-trimeric GP in 3D modeling.
6. The authors should provide more details about the coating GP antigens used in serum ELISA to ensure that the coating antigens do not contain foldon or other non-GP motifs that have been used in the vaccine antigens.

Reviewer #3

(Remarks to the Author)
In this paper titled "A Multivalent Pan-Ebolavirus Nanoparticle 1 Vaccine Provides Protection in Rodents from Lethal Infection by Adapted Zaire and Sudan Viruses", Brunette et al., report the design, characterization and testing of nanoparticles displaying Ebolavirus surface glycoproteins. The nanoparticles are prepared using the icosahedral I53-32 platform, and characterized using several techniques, including electron microscopy. The protective efficacy of these nanoparticles was demonstrated in mice and guinea pigs. This study addresses an important vaccine need against a viral pathogen.

This review focuses primarily on the EM studies. Overall, the studies are well done, and characterizations are extensive at each stage of the production.

Comments:

A major deficiency in the paper is lack of characterization of the epitopes of the antibodies elicited by vaccination. While the authors note this deficiency in the discussion, given the extensive EM data shown in the paper, which attests to the fact that the authors have access to the technology, this important characterization data for the vaccine outcome should be included in the paper.

Other comments:

In Figure 1: the legend should be revised to include details that aid in understanding the figure panels. For example, for panel g, no description is provided in the legend for how the subunits are visualized and colored.

Figure 1d: Bands in the SDS-PAGE should be labeled indicating which band represents each component. Same comment for 2k. The gels in the Supplement are better labeled.

Figure 1e: the scale bar in the figure is very hard (or impossible) to decipher.

Figure 1h: Add labels to the figure, for example, for the glycan.

The fit for the glycan in 1h (left) does not seem optimal and can be improved.

Figure 2b and 2f: scale bars are drawn with no indication for what the scales are.

Minor:

Supplemental Figure 13 legend: "..the GP is flexibly attached…"

Version 1:

Reviewer comments:

Reviewer #1

(Remarks to the Author)
The revised version of this interesting manuscript now addresses the majority of prior reviewer questions, thank you for working though the long list of topics raised. Unfortunately with some of the more substantial revisions came some inaccuracies in description of relevant prior published work. Some further topics will have to be clarified further specifically related to antigen doses.

The remaining items that should be addressed are the following:
1) it is great that more information is now included to the status of EBOV, SUDV and multivalent filovirus vaccines from various platforms including adjuvanted recombinant subunits. On the latter, in line 124 you inaccurately describe that a EBOV+MARV+SUDV GP vaccine had been tested and shown efficacy against MARV which is not described in this paper (but other combinations were tested successfully). However, another (later) publication describes a combination of SUDV+MARV GP and protective efficacy against both viruses.
2) Thank you also for including the rationale of why mouse immunizations used the subcutaneous route. But the explanation why guinea pigs were immunized via the intra-peritoneal route is still missing. As this is an unusual route not used clinically for vaccines, a rationale should be given why IM or SC routes were not used. Also, in presenting results (for example in the summarizing lines 344-346) the findings should be relativized as the IP route may have a significantly stronger immune activation resulting in the qualitatively better protection observed over the mouse model data. The same consideration should be used when discussing data, for example in lines 364-366 where you speak about a more robust cross-reactivity which could also be linked (at least partially) to the different route of administration.
3) While the discussion now presents a more complete picture of the relevant candidates in the space of other VLP, protein and nanoparticle vaccines, it should be noted that the drosophila-expressed GP-subunits (in the context of thermostabilization) have also been extensively characterized in their self-assembled trimerization state and solution stability. Mention and discussion of this fact would be relevant in the context of your added thermostability experiment as well as in the discussion in lines 368-370.
4) The revised paragraph summarizing the NP-platform capabilities has been noted, however, the final sentence (especially "promising manufacturable candidates for clinical development...") in lines 393-394 seems to be a bit too optimistic given that a lot more steps of preclinical development are needed before this can be advanced into the clinic.
5) In the final paragraph (lines 425-426) there seem to be some overstatements with regards to the ease of achieving a multivalent vaccine (that presumably protects humans). In particular it should be noted that immunogenicity and efficacy will have to be tested using a clinically acceptable injection route in a relevant NHP model first.

In materials and methods the following was noted:
1) for both the mouse and guinea pig vaccinations you make satements that immunogens were mixed 1:1 with Addavax to reach a concentration of 0.01mg/ml. If 0.1ml was given to mice that would have resulted in 1ug doses, the results section, however, states that 3.4ug total antigen were given.
For guinea pigs the calculation would indicated that 250ul injection volume given contained 2.5ug antigen. The test states that 4.3ug total were administered per immunization. Please make this consistent and clarify.
2) for the ELISA section, information about the volume per well used to coat plates is missing and the statement about the dilutions tested needs to be clarified (stating starting dilution and how many subsequent dilutions rather than the current sentence containing "gradient" twice).

Reviewer #2

(Remarks to the Author)
I have no more questions about the revised article

Reviewer #3

(Remarks to the Author)
The authors have adequately addressed my critiques (related to cryo-EM).

**Open Access** This Peer Review File is licensed under a Creative Commons Attribution 4.0 International License, which permits use, sharing, adaptation, distribution and reproduction in any medium or format, as long as you give appropriate credit to the original author(s) and the source, provide a link to the Creative Commons license, and indicate if changes were made.
In cases where reviewers are anonymous, credit should be given to 'Anonymous Referee' and the source.
The images or other third party material in this Peer Review File are included in the article's Creative Commons license, unless indicated otherwise in a credit line to the material. If material is not included in the article's Creative Commons license and your intended use is not permitted by statutory regulation or exceeds the permitted use, you will need to obtain permission directly from the copyright holder.
To view a copy of this license, visit https://creativecommons.org/licenses/by/4.0/

## RESPONSE TO REVIEWERS COMMENTS

*"A Multivalent Pan-Ebolavirus Nanoparticle Vaccine Provides Protection in Rodents from Lethal Infection by Adapted Zaire and Sudan Viruses"* by Weidle & Brunette et al. (NCOMMS-25-02639)

**Reviewer #1**

1. This manuscript is written very well with only minor suggested corrections of the text. A vast amount of supplementary data is presented in addition to high quality main figures describing research efforts towards a bivalent vaccine encompassing EBOV and SUDV components.

Thank you for your overall endorsement of the manuscript and work.

2. While the overall efforts are commendable and lead towards the potential development of interesting vaccine candidates, the present stage data do not support the conclusions, especially in the context of existing published research by other groups.

We thank the reviewer for this comment. We agree that our original conclusions did not sufficiently reflect the strength and limitations of the data when viewed alongside prior filovirus vaccine studies. We have rewritten most of the Abstract (lines 29 to 40), the Results and their interpretation (lines 149 to 350), and the Discussion (lines 352 to 432) to more accurately reflect the outcomes and trends observed in both rodent models. These revisions constitute a large portion of the entire manuscript (as opposed to single lines or statements). This was necessary to ensure that our interpretations are reframed to be fully supported by the data. The discussion is largely also rewritten to be more precise and limited only to what the results justify. These changes are highlighted in Yellow throughout the revised manuscript.

The Introduction (lines 43-147) and Discussion have both been rewritten to also better contextualize our findings within the existing literature, including prior studies using vectorized vaccines encoding multiple filoviral GPs (Pratt et al. 2010; Geisbert et al. 2009) and protein subunit vaccines containing EBOV, SUDV, and MARV GPs (Lehrer et al. 2021). Together, these additions clarify how our results fit within the broader field and how combinations of heterologous GP immunogens have been shown to generate broad neutralizing responses in previous studies. Our revised text now more accurately reflects both the current state of the field and the specific strengths and limitations of our data.

For convenience the new abstract is as follows:

*"Both Zaire ebolavirus (EBOV) and Sudan ebolavirus (SUDV) are members of the genus Ebolavirus and cause outbreaks marked by high fatality rates and repeated spillover from animal reservoirs. Filoviral glycoproteins (GPs) are the primary targets of neutralizing antibodies and form the basis of current vaccines. Here we describe the design, structural characterization,*

*and evaluation of two-component self-assembling icosahedral I53-50 nanoparticles displaying prefusion EBOV or SUDV GP antigens, either individually or in cocktail and mosaic multivalent formats. EBOV-GP-I53-50 and SUDV-GP-I53-50 nanoparticles elicited strong homologous protection in mice and guinea pigs. In the mouse-adapted EBOV model (maEBOV), mosaic and cocktail formulations produced weak survival below that of the matched EBOV-GP-I53-50 GP immunogen. In contrast, in the gpaSUDV guinea pig model (gpSUDV), cocktail and mosaic nanoparticles elicited robust protection against gpSUDV, and detectable antibody responses to both SUDV and EBOV GPs. These findings demonstrate that multivalent GP-I53-50 nanoparticle immunogens can protect rodents from death, severe clinical signs of disease, and weight loss in relevant models. Together with the established clinical safety of the I53-50 platform, these results support continued efforts toward the development of a pan-ebolavirus vaccine."*

3. For example in line 51, three references are listed of vaccine candidates with proof of efficacy in NHP's. Unfortunately not a single one showing SUDV efficacy is listed. This may mislead a less versed reader as to the current state of the field. Of particular relevance to the platform presented here, efforts related to adjuvanted protein vaccines would be necessary.

We thank the reviewer for highlighting this omission. We've revised this section to include references for both EBOV and SUDV vaccines, demonstrating protection in NHPs (lines 59-60):

"*Several species specific EBOV(Stanley et al. 2014; Marzi et al. 2015) and SUDV(Honko et al. 2024; Fletcher et al. 2025) candidates have been shown to protect against lethal infection in non-human primates (NHPs).*" We have also significantly expanded our discussion of protein based vaccines for EVD in the introduction.

4. Further incomplete data presentation includes the efficacy data from the Ebola Ça Suffit study (line 59). Here a final study report with efficacy results has been published (in 2016).

We thank the reviewer for pointing out this study. Importantly, the Ebola Ça Suffit study was mentioned in lines 57-58 of the initial submission(Henao-Restrepo et al. 2017). For our resubmission, we've added further discussion regarding publications for both Ça Suffit, as well as the larger ring vaccination study in 2019 (Preliminary results on the efficacy o...; Muyembe et al. 2024) (lines 65-69). We've added additional information about the results of the 2019 study, including its overall effectiveness, as well as vaccine effectiveness for patients already exposed and recently exposed to EBOV (lines 65-74). Additionally, we've removed the more anecdotal example of post exposure prophylaxis (Davis, C 2020). We believe this better contextualizes the current state of EVD vaccine development in the field.

5. In line 103 two references cite efforts on protein subunit vaccines against EBOV, unfortunately this is not comprehensive and is missing highly relevant candidates that would later help to position the data in the appropriate context for discussion.

We thank the reviewer for this critique. As noted above, the introduction (lines 43-147) has been extensively revised to better contextualize our research within the broader field. Specifically, we have added discussion of currently approved viral vector vaccines—Ervedo, Zabdeno/Mvabea (Ad26.ZEBOV/MVA-BN-Filo), and GP (ChAdOx1 biEBOV). While these vaccines have demonstrated efficacy against EBOV, none provide broad protection against both EBOV and SUDV. This limitation highlights the need for continued vaccine development to achieve broad protection, which is the central objective of our study involving the co-display and co-immunization of EBOV and SUDV GPs on the I53-50 nanoparticle. We have also expanded our discussion (lines 352-432) to include several preclinical vaccine efforts addressing this goal. Notably, the viral vector vaccine ChAdOx EBO7, which includes both SUDV and EBOV GP, elicits broad protective responses in NHPs. In addition, the adjuvanted protein-based vaccine described by Lehrer et al. ("Recombinant protein filovirus vaccines protect Cynomolgus macaques from Ebola, Sudan, and Marburg viruses") uses soluble EBOV, SUDV, and Marburg GP protein ectodomain to induce broad and protective immunity in NHPs. These examples illustrate that broad protection can be achieved through both viral vector and protein-based adjuvanted vaccines utilizing GP immunogens, and help contextualize our I53-50 nanoparticle vaccine approach within the broader field.

6. For the animal study design, many important aspects are missing that would be highly relevant in terms of reproducibility. The authors fail to describe why the subcutaneous route was chosen for both mouse and guinea pig studies, but also what exact (GP) antigen dose was given to mice. For both animal species injection volumes were also omitted.

We thank the reviewer for this comment. The subcutaneous route of immunization was selected following guidance from the University of Washington IACUC committee, to avoid potential pain at the injection site that could ensue from intramuscular delivery (now added in the Method section (lines 624-625). Accurate (GP) antigen dosage and injection volumes for both rodent studies in results (mice: lines 255-268), (guinea pigs: lines 307-317)

7. It has to be noted that the efficacy levels against EBOV in the mouse model are not truly impressive, especially in the context of other vaccine candidates and also the SUDV efficacy in the guinea pig model is not much stronger. The data indicates some early achievements, however, rodent models of filovirus infection are not very predictive of efficacy in (relevant) primate models.

We thank the reviewer for this comment and have reworked the discussion (lines 352-432) to better represent the achievements of the work presented. NHP studies were beyond the scope of this work.

8. In the discussion the rather long section about passive immunotherapies should (in line 308) more clearly describe that ZMapp protects against EBOV and then the latter mAb cocktails against SUDV are still under development.

Thank you to the reviewer for this comment. Having a closer look at this section we agree with the reviewer that the current wording was ambiguous. Upon further review the discussion on mAbs was removed entirely from the text as the primary focus of this work is on vaccines. In its stead, additional information focusing on the current state-of-the-art in vaccine development was added to the Discussion (lines 373-385).

9. The section discussing structural findings is overall fine, however, the prefusion trimeric EBOV-GP that was analyzed was a truncated version as described in detail. The absence of larger sections such as the mucin-like domain could very well have further implications in the accuracy of the modeled protein structure.

We thank the reviewer for this note. As a follow up, we looked further into the published literature that examined WT GP on virions and VLPs (Beniac, 2017) (Tran, 2014). These structures both observe only low-resolution density for the mucin-like domain. We added the following information to the main text (lines 401-411):

*"Additionally, the glycan cap (residues 224–310) was unresolved in our cryoEM structure. This was an interesting observation, as this domain is resolved in the aligned crystal structure sharing the same sequence (PDB ID: 5JQ3). We hypothesize that this is likely due to stabilizing crystal packing effects for these domains. An alternative hypothesis for a lack of observable glycan cap density in our map could be due to the presence of a cathepsin cleavage site at Lys190, which removes the glycan cap and mucin domains to facilitate viral entry(Dube et al. 2009; Brecher et al. 2012; Lee and Saphire 2009). However, clear density in our map for residues 191–195 and 216–223 indicates that no cleavage had occurred at this site – a result consistent with our SDS–PAGE analysis (**Figure 1d**). Therefore, the absence of glycan cap density in the cryoEM maps likely reflects the weak attachment of this region to the GP core domains, as previously hypothesized by other groups(Yu et al. 2023; Murin et al. 2021), and may also explain the lack of glycan cap density in their antibody-bound cryoEM structures. Notably, the absence of the mucin-like domain may further contribute to destabilization of the glycan cap in our structure. As a result, the mucine-like domain has still only been resolved to low resolution on virions and VLPs using cryoEM (Tran et al. 2014; Beniac and Booth 2017)."*

10. Lines 343-344 state that virus neutralizing titers could not be obtained due to BSL-4 requirements, however there are several BSL2-level surrogate assays (based on rVSV or lentiviruses) available that could have easily been used and would have strengthened the dataset, in particular as a potential readout to be correlated with survival in the rodent models.

We thank the reviewer for this comment. We have removed this comment on the limitation of BSL-4 requirements to obtain neutralization titers from the paper. Overall our studies were limited by the amount of sera obtained from the rodents and what serum we obtained was used for the quantitative ELISA studies used to measure anti-GP IgG. Unfortunately, the remaining serum was not sufficient to conduct pseudo-neutralization assays. The expense and time to conduct additional studies is beyond the scope of this study and our resources. We do note this in the limitations (lines 413-424) section of the discussion as something important for future studies.

9b. In the further hypothetical discussion of what all could be included (even though the current candidate does not meet the mark yet), Marburg virus is implied to also be an Ebolavirus (line 349). And the next line speaks only about EVD-causing filoviruses (what about MVD?).

This typo has been corrected.

11. The entire paragraph lines 358-370 sounds like positioning a technology for a grant or contract, but at the current stage of development, I don't think that has a place in the current manuscript.

We thank the reviewer for the comment. We have substantially revised this paragraph to better situate the I53-50 NP platform within the broader literature. The updated text now places appropriate context on why the I53-50 nanoparticle platform is of interest relative to other protein-based vaccine approaches for broad-spectrum EVD prophylaxis, without overstating its advantages. We now reference that the I53-50 NP platform has completed regulatory approvals for SKYCovione™ and RSV/HMPV candidates and note its growing safety profile. The paragraph now concludes with the following conservative sentence (lines 384-385):

*“Collectively, these results position the EBOV- and SUDV-GP-I53-50 nanoparticle immunogens as potentially promising manufacturable candidates for clinical development of broadly protective ebolavirus vaccines.”*

12. I also think lines 378-381 are highly speculative as at this point it is hard to predict whether primates will be protected with the nanoparticle vaccine in its current form, so discussing a potential boost in people is extremely speculative as an ineffective booster could cause more harm than do good.

We agree with the reviewer and have removed this statement from the manuscript.

13. In M&M I noticed some things...section 549-567 is missing a lot of details on vendors,

We reviewed this section, and added two additional vendors (lines 595-619).

14. line 553 misspelling of "Whatman"

This spelling has been corrected. (line 601)

15. missing volumes/doses in both animal studies

Thank you for bringing this to our attention. Volumes and doses have been added to the manuscript for all studies in results (mice: lines 255-268), (guinea pigs: lines 307-317)

16. line 601: after giving excellent detail on the vaccine antigens, some more information on the coating antigens for ELISA are needed to replicate and position immunogenicity results. Also no description is given on what dilutions were analyzed on the ELISA plates.

This information about the full-length GPs produced and purified for ELISAs is now included in the manuscript. Additionally, the ELISA protocols were revised to include dilutions used.

17. Figure 5 (legend) line 714 speaks of mice when it should read guinea pigs…

This error has been corrected.

18. Supplemental figure 2 - the text speaks of I53-50A, the figure itself (line 829) says "153"

This typo was corrected.

**Reviewer #2**

19. In this manuscript, Brunette et al. developed and characterized a series of protein nanoparticle vaccines against two species of the ebolavirus genus, EBOV and SUDV. The authors displayed mucin-like domain (MLD)-deleted glycoprotein (GPMuc) trimers on the I53-50 2-component nanoparticle (2c-NP) platform, determined their atomic-level structures by cryo-electron microscopy (cryo-EM), and carried out challenge studies in mice and guinea pigs to demonstrate protection conferred by either cocktail or mosaic vaccines. This work was highly similar to previous studies done by the same group of authors: they utilized the I53-50 2c-NP platform to display either wildtype viral antigens or rationally designed viral antigens licensed from other institutions as vaccine candidates, with supporting data generated through structural analyses and in vivo assessment. There is no doubt about the importance of a broadly

protective ebolavirus vaccine, and the logic and rationale of the study are clear. The animal studies were well-designed; we were particularly impressed by the inclusion of matched “non-assembling” subunit controls as well as bare I53-50 2c-NPs.

Thank you for your overall endorsement of the manuscript and work.

20. However, their vaccine construct design is outdated and could be significantly improved by incorporating recent advances in rational GP design and stabilization. Given the suboptimal GP design, we have concerns about the stability of the EBOV, SUDV, and mosaic NP constructs and would like to see data addressing this.

We deliberately selected the most native form of the prefusion GP trimer possible. Extensive characterization confirmed that the GP immunogens in our nanoparticle vaccines are well-folded prefusion trimers. To address the reviewer’s concern regarding potential stability, we conducted thermal stability assays (lines 697-708 in methods) on all nanoparticle and GP immunogens described in this study. For all GP-containing constructs, a primary thermal melting temperature ($Tm_1$) of approximately 55°C was observed, which was not negatively affected by scaffolding onto the I53-50A component. These results indicate that the nanoparticle immunogen constructs used in this study are stable at, and well above, physiological temperatures relevant to vaccination and immunological studies. These results and analysis are included as part of Supplemental Figure 14 and we added the following description to the Results section (lines 240-254) of the revised manuscript:

*“To assess thermal solution stability of both the GP component proteins and assembled NP immunogens, we conducted differential scanning fluorimetry (using the nanoDSF UNcle instrument of Unchained Laboratories) under a thermal ramping regime while measuring the intrinsic tryptophan fluorescence of the protein samples (**Supplemental Figure 14**)* (lines 1081-1089)*. The I53-50A component remained stable with no detectable unfolding even at the upper temperature limit, whereas the I53-50B component showed a gradual transition beginning near 80°C. However, when I53-50A and I53-50B were assembled into the full nanoparticle, no unfolding transitions were observed for either component, indicating enhanced stability of the assembled I53-50 NP. In contrast, GP-containing components exhibited an initial melting transition ($Tm_1$ ≈ 55°C) for all GP and GP-containing NP immunogens, including EBOV-GP-Foldon (MDT-000759), EBOV-GP-I53-50A (MDT-000868), SUDV-GP-I53-50A (MDT-000869), EBOV-GP-I53-50 NP, SUDV-GP-I53-50 NP, EBOV/SUDV-GP-I53-50 Mosaic NP, and EBOV + SUDV I53-50 Cocktail NP. Constructs with the foldon trimerization domain displayed a secondary transition near 80°C, while substitution of foldon with I53-50A shifted this event upward to approximately 88°C. This biphasic behavior was retained upon nanoparticle assembly with I53-50B. We interpret these transitions as representing sequential destabilization of the GP trimer at ~55°C (Powell et al. 2022; Rutten et al. 2020) and potential unfolding of a either postfusion state or individual GP1/GP2 monomers at higher temperature(Weissenhorn et al. 1998; Harrison et al. 2011) (**Supplemental Figure 14**). These results demonstrate that all EBOV and SUDV constructs retained structural integrity at temperatures well above*

*physiological levels, indicating that they are likely to remain structurally stable during subsequent injection and immunization studies.”*

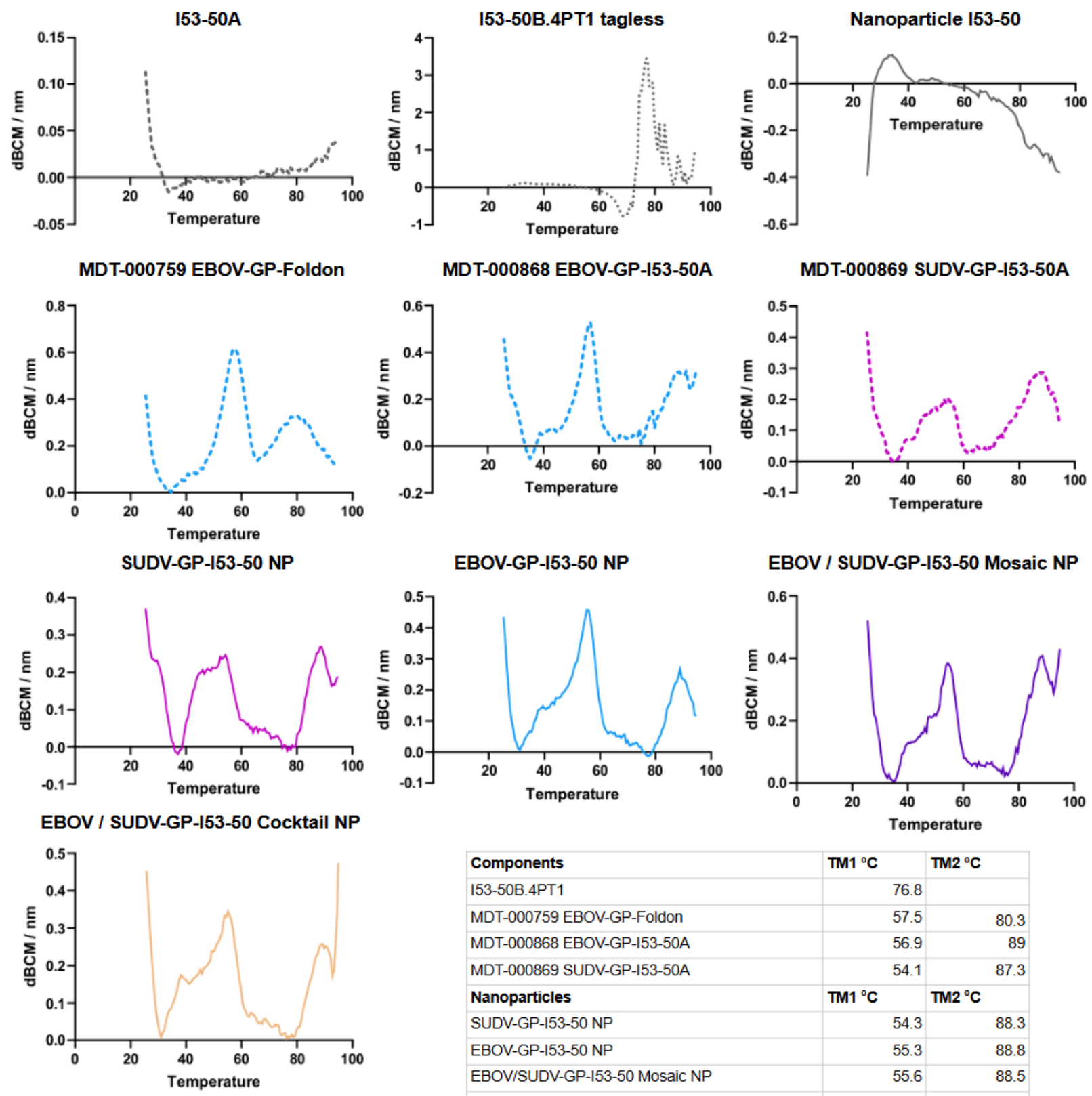


| Components | TM1 °C | TM2 °C |
|---|---|---|
| I53-50B.4PT1 | 76.8 | |
| MDT-000759 EBOV-GP-Foldon | 57.5 | 80.3 |
| MDT-000868 EBOV-GP-I53-50A | 56.9 | 89 |
| MDT-000869 SUDV-GP-I53-50A | 54.1 | 87.3 |
| **Nanoparticles** | **TM1 °C** | **TM2 °C** |
| SUDV-GP-I53-50 NP | 54.3 | 88.3 |
| EBOV-GP-I53-50 NP | 55.3 | 88.8 |
| EBOV/SUDV-GP-I53-50 Mosaic NP | 55.6 | 88.5 |
| EBOV/SUDV-GP-I53-50 Cocktail NP | 55.1 | 89.2 |

**Supplemental Figure 14. Thermal stability of EBOV- and SUDV-GP nanoparticle immunogens by differential scanning fluorimetry.** EBOV-GP-I53-50 and SUDV-GP-I53-50 nanoparticle component fusions and assembled nanoparticles (0.10 mg/mL) were analyzed by intrinsic tryptophan fluorescence using a UNcle instrument (Unchained Labs) to determine melting transitions (Tm). Fluorescence intensity ratios at 330 nm and 350 nm were monitored as a function of temperature from 25°C to 95°C (0.15°C $min^{-1}$; acquisition interval = 10 seconds). The I53-50A component exhibited no detectable unfolding across the full temperature range, while I53-50B showed a gradual transition beginning near 80°C. Upon assembly, the I53-50 nanoparticle remained stable with no unfolding transitions observed for either component, indicating enhanced stability upon assembly. In contrast, GP-containing components and nanoparticles exhibited a biphasic melting ($Tm_1$ ≈ 55°C and ($Tm_2$ ≈ 80–88°C). Reported Tm values represent the mean of 10 replicate acquisitions per sample.

21. More concerningly, the authors’ discussion of their own data is selective and, in some cases, misleading in what appears to be a deliberate attempt to promote mosaic and/or 2c-NP vaccines with little supporting data. I suggest that the authors address these issues with rigorous experimental data and revise the writing.

Thank you for this comment. We agree that the phrasing in the original version could be improved for clarity. In response, we have revised the Introduction, Results, and Discussion to ensure that they reflect the observed data without ambiguity. We have also added the following text to the Results and Discussion, respectively, to provide clearer context for the performance

of the EBOV- and SUDV-GP-I53-50 nanoparticle immunogens and to better highlight the significance of our findings:

*"Overall, survival outcomes in the gpaSUDV guinea pig model were markedly better than those observed in the maEBOV mouse challenge. Mosaic and cocktail EBOV/SUDV-GP-I53-50 NP immunogens protected 5 of 6 animals in each group (83% survival), and the strain-matched SUDV-GP-I53-50 NP conferred complete protection, all substantially outperforming the 20-30% survival seen in the maEBOV study's mosaic and cocktail GP-I53-50 groups. These findings highlight the strong protective capacity of SUDV-GP–bearing, mosaic, and cocktail I53-50 NP immunogens in a stringent BSL-4 guinea pig model and underscore their potential value as broadly protective filovirus vaccine candidates (**Supplemental Figure 22**)."*

*"The cocktail or mosaic NP immunogens formulated with AddaVax™ adjuvant elicited robust anti-GP IgG immune responses to both EBOV-GP and SUDV-GP, with ELISA titers similar to that observed in either mice or guinea pigs dosed with 2x GP antigen mass equivalents as individual NP immunogens. However, survival responses observed for the mosaic or cocktail NP immunogens in the maEBOV murine challenge (Figure 4f), while still statistically significant when compared to the SUDV-GP-I53-50 group (Supplemental Figure 22a), were far below the protection achieved by the strain-matched EBOV-GP-I53-50 NP immunogen. In contrast, in the gpaSUDV guinea pig challenge (Figure 5f), strain-matched SUDV-GP-I53-50 NP immunization conferred complete protection (6/6 survivors), and both the cocktail and mosaic EBOV/SUDV-GP-I53-50 NP formulations afforded high levels of protection (5/6 survivors in each group). These outcomes were significantly better than those observed for the EBOV-GP-I53-50 NP group (1/5 survivors) (Supplemental Figure 22b) or the bare I53-50 NP control group (0/6 survivors). Together, these two independent BSL-4 rodent studies indicate that cocktail and mosaic EBOV/SUDV GP I53-50 NP immunogens can provide statistically significant dual protection against lethal EBOV and SUDV challenge (Supplemental Figure 22) as compared to heterologous single-antigen NP immunogen-dosed groups, while their relative performance compared to homologous single-antigen NP immunogen-dosed groups depends on the specific challenge model. Additionally, SUDV-GP-I53-50 NP immunization elicited detectable cross-reactive anti-EBOV GP antibody responses in guinea pigs (Figure 5b), whereas no EBOV↔SUDV cross-reactivity was observed in the maEBOV mouse model (Figure 4b). Together, these findings highlight that the guinea pig model demonstrates broader cross-reactivity and more robust heterologous protection than the maEBOV mouse model, aligning more closely with Immunogenicity patterns observed for E2p NP EBOV GP trimers in immunized rabbits(He, L. et al. 2020)."*

*"Importantly, our results show that rationally designed EBOV and SUDV GP I53-50 nanoparticle immunogens form well-behaved, properly folded prefusion trimers that assemble efficiently and elicit strong protective immunity in homologous challenge models. The mosaic and cocktail formulations induced cross-reactive antibody responses in both mice and guinea pigs and provided clear protection against gpSUDV challenge in guinea pigs, supporting continued development of multivalent GP I53-50 nanoparticles for broader filovirus coverage. Additional refinement of antigen composition, adjuvant formulation, and immunization strategy may further*

*improve the breadth and durability of protection against multiple Ebolavirus species in future clinical applications."*

22. Major comment #1:

This study was built upon the hypothesis that a cocktail or a mosaic vaccine containing both EBOV and SUDV components will protect against these two ebolavirus species more effectively than a vaccine based on a single ebolavirus GP backbone. This hypothesis is inconsistent with a large body of antibody work published over the last decade, with panels of potent pan-ebolavirus neutralizing antibodies (nAbs) isolated from human survivors (e.g., ADI-15878 and ADI-15946) or immunized nonhuman primates (e.g., CA45). The elicitation of pan-ebolavirus nAbs during infection and vaccination provides strong evidence for the development of broadly protective ebolavirus vaccines based on a single ebolavirus GP backbone.

Thank you for pointing out this research on broad spectrum EVD nAbs elicited in survivors of EVD. In the study noted, 349 antibodies were isolated and characterized from 2014 EBOV survivors. Of these antibodies "*only 11% were cross reactive with SUDV GP*", and only "*10 of 37 cross-reactive mAbs showed significant neutralizing activity against rVSV-SUDV GP*." These cross reactive antibodies show potential for therapeutic mAbs to combat pan-EBOLA infection. At the same time, these results show that exposure to only EBOV GP generates a largely specific response to EBOV with the bulk of neutralizing antibodies offering little to no protection against a SUDV infection. This evidence alone points to the need for both EBOV and SUDV GP exposure to develop broadly neutralizing antibodies against both strains, while a single GP can develop broadly neutralizing antibodies; these examples are rare and unique (Wec et al. 2017).

The following example has also been added to the text (lines 83-88):
*"Additionally, a phase 1 clinical trial using only ZEBOV GP encoded by the viral vector chimpanzee adenovirus (ChAd3) generated no detectable SUDV GP-specific antibodies at 28 days post dose, despite generating strong levels of EBOV neutralizing antibodies(Ewer et al. 2016). Anti-SUDV GP antibodies could only be observed in individuals following a boost with a modified Vaccinia Ankara (MVA) vector (MVA-BN Filo - Mvabea(Woolsey and Geisbert 2021)), which includes the same ZEBOV GP as ChAd3, plus the GPs of SUDV and Marburg virus (MARV) and the nucleoprotein of Taï Forest Ebola virus (TAFV)."*

23. Therefore, the lack of cross-ebolavirus antibody response observed in this study, as evidenced by lines 221-223 "Little or no cross-reactive antibody responses were observed: only 3 out of 10 animals that received EBOV-GP-I53-50 had any observable anti-SUDV-GP antibodies, while none of the ten animals that received SUDV-GP-I53-50 had any appreciable anti-EBOV-GP antibodies," was very concerning, suggesting potential problems with vaccine construct design. In fact, the He et al. study in 2021 showed that EBOV GP-NP-induced sera were cross-reactive and robustly neutralized pseudotyped BDBV, another ebolavirus species. The statement in the next sentence "This was expected as the EBOV and SUDV GP

components of our NP immunogens have only 68% sequence identity (Supplemental 225 Figures 18 and 19), and previous reports on filovirus vaccine candidates have suggested that little heterologous cross-protection can be obtained with single filoviral GP immunogens." is purposedly confusing

We appreciate the reviewer's comment and have clarified this point in the manuscript. In our study, cross-reactive anti-GP antibodies were not observed in the maEBOV mouse model, consistent with the statement originally cited. However, in the gpaSUDV guinea pig model, guinea pigs immunized with SUDV-GP-I53-50 demonstrated a clear cross-protective response against EBOV, and the mosaic and cocktail NP immunogens elicited measurable cross-reactive anti-EBOV GP IgG responses.

To make this distinction clear, we have revised the text in the maEBOV section to read (lines 275-277):

*"The EBOV and SUDV GP components of our NP immunogens share only 68% sequence identity (Supplemental Figures 20 and 21). In this maEBOV mouse model, this level of divergence was insufficient to elicit strong EBOV↔SUDV cross-neutralizing antibody responses."*

We have also added the following to the Discussion section (lines 365-371):

*"Cocktail and mosaic EBOV/SUDV GP I53-50 nanoparticles generated strong antibody responses and provided significant protection in both BSL-4 rodent models, although their performance relative to strain-matched single-antigen immunogens varied by challenge system. Strain-matched immunogens gave the highest survival in each model, and cross-reactivity was observed only in guinea pigs, not in mice. Together, these findings highlight that the guinea pig model demonstrates broader cross-reactivity and more robust heterologous protection than the maEBOV mouse model, aligning more closely with immunogenicity patterns observed for E2p NP EBOV GP trimers in immunized rabbits(He et al. 2021)."*

24. As leading experts in protein structure prediction, the authors must know that 68% sequence identity means high homology in protein structure and function,

While we agree that 68% sequence identity reflects a high degree of structural and functional homology, we do not agree that this level of similarity necessarily predicts cross-neutralizing antibody responses across all vaccine platforms or all animal models. In our study, 68% identity was insufficient to elicit a cross-reactive response in the maEBOV mouse model, whereas clear cross-reactivity was observed in guinea pigs. Thus, although the reviewer's point is valid in principle, our data show that this degree of sequence conservation did not uniformly translate into cross-neutralization across model systems, despite strong structural and biochemical evidence that all antigens were well-folded and thermally stable (see new Supplemental Figure 14 for thermal assays included during revision and our response to points 20 and 23).

24.b. and furthermore, "filovirus" is different from "ebolavirus" in scope, as the former contains several diverse viral families and the latter has 6 species including EBOV and BDBV, which are closely related. Similar misleading statements can be found throughout the manuscript.

We thank the reviewer for this comment and have scaled back the language to clarify the focus here is creating a Pan-EbolaVirus Vaccine.

25. Major comment #2:

The vaccine constructs used in this study (lines 140-153), appeared to not have considered recent advances in rational GP design and stabilization (Rutten et al., 2020, Cell Rep 30, 4540–4550; He et al., 2021, Nat Commun 12, 2633). These constructs only included basic modifications that were used in early studies to produce soluble GPs, such as removal of the membrane-proximal external region (MPER) and MLD, as well as the T42A mutation. None of the critical stabilizing mutations reported in the Rutten and He et al. papers, such as the proline mutations in HR1c and various mutations in HR2, which substantially improved GP trimer formation and stability, was tested in this study. Due to the use of a less table, nearly wildtype GP, the resulting EBOV-GP-foldon and SUDV-GP-foldon constructs produced trimeric, dimeric, and monomeric GP species, as indicated by the SEC profile after IMAC purification (Figure 1C). The GP trimer stability problem became worse when it was directly attached to the I53-50A trimer without a foldon motif, as indicated by the SEC profile (Supplemental Figure 2). Even though the trimer fraction can be extracted through repeated SEC purifications, due to the inherent GP instability this GP trimer may not remain stable when displayed on the 2c-NP surface, adjuvanted with AddaVax adjuvant, and mixed with body fluids at an elevated (body) temperature after immunization.

To address this comment, we conducted additional (Supplemental Figure 14) differential scanning fluorimetry experiments to measure the thermal stability of our EBOV GP and SUDV GP NP immunogens (with foldon) and observed that the GP components as fusions to I53-50A trimer component, or as assembled I53-50 NPs demonstrated a two phase unfolding pattern (Tm1 = 55°C and Tm2 = 80-88°C), indicating that the GP immunogens reported here are stable to at least 50°C, above that of body temperatures for rodents or humans.

We recognize that GP point mutations can be introduced to enhance thermal stability. However, we did not pursue this strategy in our work, because our study was designed from the outset to focus on deletion constructs and was started before the referenced papers using stabilized constructs were published. Thus, we had focused on removing the mucin and MPER transmembrane domains, rather than introducing stabilizing point mutations. We also selected the Zaire Mayinga strain of EBOV GP rather than the Mano River variant that has been reported to exhibit reduced pre-fusion stability at room temperature, which we also observed.

Our Supplemental Figure 2 shows that Mano River GP without the addition of a foldon and genetically fused to I53-50A existed as two molecular species by SEC, and was therefore not selected for further investigation. In contrast, the fusion of either Zaire Mayinga EBOV GP (delta mucin, delta transmembrane domain) or SUDV GP (delta mucin, delta transmembrane domain) to foldon and I53-50A resulted in stable pre-fusion constructs suitable for I53-50 NP assembly as demonstrated in Figures 2 and 3, and that these constructs are stable up to 55°C when incorporated into I53-50 NPs (Supplemental Figure 14).

26. Structurally, I53-50 is not suitable for displaying glycoprotein trimers with a narrow stalk, e.g., filovirus GP, because its N-terminus, the trimer-anchoring site, forms a wide triangle (~50 Å). In the current EBOV/SUDV-GP-foldon-I53-50 constructs, foldon is included to overcome the GP's inherent instability and I53-50's structural incompatibility, but itself has no biological functions. It is worth pointing out that these two problems can be dealt with using previously reported GP-stabilizing mutations and a modified I53-50 NP backbone.The authors should determine the thermostability of their EBOV and SUDV GP trimers and I53-50 2c-NPs using differential scanning calorimetry (DSC) and include DSC profiles in revision.

We addressed this comment in answer to point #25 above.

27. Major comment #3:

In this manuscript, the authors used SKYCovione, which is a COVID-19 vaccine based on the I53-50 2c-NP, as a successful example to support the ebolavirus vaccine study based on the same platform. SKYCovione was approved for human use in South Korea and the UK but not in the US. It was reported in 2022 that in the phase 1 trial conducted in the US the vaccine group underperformed the placebo group, causing concerns about potential flaws in the 2c-NP platform. In the phase 3 trial conducted in South Korea, SKYCovione was compared with AstraZeneca's vector vaccine and showed ~2-fold increase in potency. However, AstraZeneca's vector vaccine was known to underperform mRNA vaccines by ~4-fold, suggesting that SKYCovione would seem less effective if it was compared directly with mRNA vaccines. If the authors intended to use the SKYCovione data, it would be important to provide a complete and unbiased background about the development of this vaccine, including both positive and negative data.

We thank the reviewer for making this observation, and have rewritten the Discussion (lines 352-432) on the I53-50 NP platform to focus on its safety and manufacturability profile. We compare our current pre-clinical data on recombinant protein production levels for our EBOV and SUDV GP fusion constructs to the I53-50A component relative to that reported on the pre-clinical production levels of other viral immunogens as fusions to I53-50A.

28. Major comment #4:

In lines 366-370, the authors discussed single-component and SpyTag/SpyCatcher NP systems, which were incomplete or inaccurate. First, He et al. demonstrated that both I3-01 and E2p 60-mers can be used to display stabilized EBOV GP trimers, with E2p showing greater structural compatibility with GP and eliciting more potent nAbs. However, E2p was omitted throughout the paper.

To address this comment, we have reworded the reference to I3-01 and E2p to say the following (lines 373-385):
*"While protein-based nanoparticle Ebola vaccine candidates have been reported using full-length GP purified from insect cells as rosette aggregates(Bengtsson et al. 2016; Hahn et al. 2015), or as single-component nanoparticle vaccine candidates based on I3-01(He et al. 2021; Hsia et al. 2016), E2p(He et al. 2021; Powell et al. 2022), or ferritin(Xu et al. 2024), these nanoparticle systems lack the ability to easily co-display multiple antigens. However, this can be readily accommodated by the two-component I53-50 NP. Mosaic nanoparticles can alternatively be produced using the SpyTag/SpyCatcher system(Bruun et al. 2018; Zakeri et al. 2012). A SARS-CoV-2 vaccine based on the two-component nanoparticle scaffold used in this study was licensed for use in both the United Kingdom and South Korea and was granted Emergency Use Listing by the World Health Organization in 2023(Feng et al. 2023; Arunachalam et al. 2021; Jacob-Dolan et al. 2023). The I53-50 NP has a growing safety profile with multiple clinical trials completed for SKYCovione™ and RSV/HMPV(Marcandalli et al. 2019), demonstrating that scalable manufacturing of I53-50-based vaccines is not likely to present a roadblock to further clinical development of the EBOV- and SUDV-GP-I53-50 vaccines. In our hands, the production EBOV-GP-I53-50A and SUDV-GP-I53-50A components was about four-fold lower (~7.3 and 7.6 mg/L by transient transfection, respectively* ***(Supplemental Figure 9)*** *than that observed for SARS-CoV-2 RBD-I53-50A fusion proteins (Walls et al. 2020). Collectively, these results position the EBOV- and SUDV-GP-I53-50 nanoparticle immunogens as potentially promising manufacturable candidates for clinical development of broadly protective ebolavirus vaccines."*

28. Second, the statement on the inability of single-component NP to create mosaic vaccines is inaccurate. Although the 2c-NP system can readily incorporate multiple antigens by mixing different antigen-I53-50A trimers with I53-50B pentamers, the ratio and surface location of different antigens upon assembly cannot be controlled. Single-component NP systems can be used to create mosaic vaccines by mixing different antigen-NP plasmids during expression but face similar challenges with controlling the antigen ratio and surface location on the NPs. Third, given the inherent instability of the current GP and GP-foldon-I53-30 designs, the mosaic display of EBOV and SUDV GP trimers will further reduce NP stability and cause product heterogeneity. Fourth, the animal data didn't show a clear advantage of mosaic vaccines vs. cocktail vaccines.

To address this comment, we have made several changes to the Introduction (lines 43-147) and Discussion (lines 352-432) as noted in other above responses; including the thermal stability assessment of our EBOV-GP I53-50 and SUDV-GP I53-50 NP immunogens which are stable to at least 50°C. See above points and point 29, below.

29. The authors are encouraged to perform the DSC analysis of mosaic vs. cocktail I53-50 2c-NPs and compare their in vivo stabilities after immunization.

Thank you to the reviewer for this suggestion. We conducted additional differential scanning fluorimetry experiments (Supplemental Figure 14) to measure the thermal stability of our EBOV GP and SUDV GP NP immunogens (with foldon) and observed that the GP components as fusions to I53-50A trimer component, or as assembled I53-50 NPs demonstrated a two phase unfolding pattern (Tm1 = 55°C and Tm2 = 80-88°C), indicating that the GP immunogens reported here are stable to at least 50°C, above that of body temperatures for rodents or humans.

30. Major comment #5:

Immunogenicity data raises some concerns about the conclusions drawn from this study. First, survival data (Fig. 4f) demonstrates that EBOV-GP-I53-50 alone protects significantly better (80%) against maEBOV challenge compared to mosaic and cocktail EBOV-I53-50-GP + SUDV-GP I53-50 (20-30%), which is not consistent with the ELISA results, where these groups show similar binding against EBOV GP (Fig. 4b). This suggests that the binding antibodies elicited in mice by the cocktail and mosaic vaccines are non-neutralizing/non-Fc-mediated and thereby non-protective compared to antibodies elicited by EBOV-GP-I53-50 alone. This observation is not addressed adequately in Discussion.

The reviewer is correct in noting that we have not addressed the specific basis for observed anti-GP responses by ELISA vs virus neutralizing vs protection from maEBOV. We have noted the limitations of our work in the Discussion as follows (lines 413-424):

*"There are several limitations to the studies reported here, mainly due to limited sera and the logistical constraints associated with conducting repeat BSL-4 studies. Our serological analyses of anti-GP IgG responses were based purely on ELISA assays. Furthermore, we have not examined the epitope specificities or structures of monoclonal or polyclonal antibodies elicited by our NP immunogens, and therefore do not know if similar antibodies are induced by the mosaic and cocktail versions of our GP-I53-50 NP immunogens. In addition, our NP immunogens lacked the MPER region of GP, which is known to be targeted by broadly neutralizing antibodies(King et al. 2019; Rijal and Donnellan 2023; Flyak et al. 2018). However, display of an MPER-based epitope scaffold on the computationally designed two-component I53-40 nanoparticle failed to elicit neutralizing antibodies after rabbit immunization(Schoeder et al. 2022). It remains to be determined if inclusion of the MPER domains in prefusion-stabilized trimeric GPs displayed on nanoparticles would further improve cross-reactive vaccine-elicited antibody responses. Future research would also benefit from an exploration of the nature of T cell responses to our GP-I53-50 NP immunogens(Walls et al. 2020), the removal of His6 tags from the GP-NP immunogens, and dose range or prime-boost optimization of the mosaic or cocktail NP immunogens (versus their non-NP/assembled counterparts) for improved broad spectrum EBOV - SUDV protection."*

31. A key analysis that is missing here is the assessment of nAb responses elicited by these vaccines, which may better explain the discrepancy between IgG binding and survival post-challenge. An explanation to the absence of nAb data is attributed to BSL-4 facility expenses (lines 342-344). However, BSL-2-level pseudovirus assays using small blood volumes have been routinely carried out to evaluate filovirus vaccine-induced nAbs and are critical to inform conclusions for this study.

Thank you to the reviewer for raising this point. As noted in our response to point 10, our study did not include sufficient serum material or financial resources to perform full neutralization analyses, and this limitation is acknowledged in the discussion. The additional experiments suggested by the reviewer would be valuable, but they are beyond the scope of the present study.

32. Next, it is surprising that "non-assembled" counterparts to the 2c-NP vaccines (groups 5-7), which contain soluble EBOV GP and/or SUDV GP trimers, could not elicit any GP-specific binding responses nor significant protection against the maEBOV challenge. This result further brings into question the use of these EBOV GP and SUDV GP trimers as immunogens for eliciting GP-specific protective antibody responses.

We thank the reviewer for noting the marked difference in anti-GP serological responses between the I53-50–assembled immunogens and the non-assembling 2OBX controls (groups 5–7). This difference is expected and is consistent with multiple published studies showing that multivalent nanoparticle display of viral glycoproteins significantly boosts immunogenicity compared to the same antigens delivered as soluble trimers. We now emphasize this point more clearly in the manuscript (lines 362-365):

"*The weak antibody responses and lack of survival in the groups of mice receiving the non-assembling 2OBX control immunogens demonstrate that multimeric NP display of the GP antigen is key to eliciting a strong and protective antiviral immune response, as has been observed for both influenza, and SARS-CoV2, RSV, EBV and HIV viral antigens(Ellis et al. 2021; Chao et al. 2024; Boyoglu-Barnum et al. 2021; Marcandalli et al. 2019; Kato et al. 2020; Malhi et al. 2022).*"

Our observation that the non-assembling 2OBX groups showed little to no detectable anti-GP IgG can be explained by the ELISA assay's lower limit of detection (LOD). The LOD for reciprocal endpoint titers was $10^2$, while the matched I53-50–assembled immunogens elicited endpoint titers in the $10^3$ to $0.5 \times 10^4$ range. This corresponds to only a 10–100-fold difference, which translates to an improvement of approximately 10–50-fold attributable to nanoparticle assembly on a per-mass basis. This degree of enhancement is consistent with prior characterizations of I53-50 nanoparticle immunogens compared to their non-assembling counterparts.

We have also now noted in the limitations paragraph (lines 413-424) in the discussion (lines 417-424) several considerations regarding our choice of immunogens:

"*In addition, our NP immunogens lacked the MPER region of GP, which is known to be targeted by broadly neutralizing antibodies(King et al 2019, Rijal et al 2023, Flyak et al 2018). However, display of an MPER-based epitope scaffold on the computationally designed two-component I53-40 nanoparticle failed to elicit neutralizing antibodies after rabbit immunization[96]. It remains to be determined if inclusion of the MPER domains in prefusion-stabilized trimeric GPs displayed on nanoparticles would further improve cross-reactive vaccine-elicited antibody responses. Future research would also benefit from an exploration of the nature of T cell responses to our GP-I53-50 NP immunogens(Walls et al. 2020), the removal of His6 tags from the GP-NP immunogens, and dose range or prime-boost optimization of the mosaic or cocktail NP immunogens (versus their non-NP/assembled counterparts) for improved broad spectrum EBOV - SUDV protection.*"

We have also added the following information to the Introduction (lines 115-127) to better frame our specific GP constructs against others used in the field:

*"…several preclinical vaccine platforms have shown cross-ebolavirus and even broader filovirus protection in NHP studies. A bivalent single complex adenovirus-based viral vector (CAdVax) that expresses EBOV and SUDV GP (EBO7) provided protection to NHPs with a single dose, with 3/3 and 2/3 NHPs surviving an aerosol challenge with EBOV or SUDV respectively without any signs of clinical disease(Pratt et al. 2010). Furthermore, a third group of NHPs that received a prime boost immunization with EBO7 saw a 100% survival rate with 3/3 NHPs surviving an aerosol challenge with SUDV(Pratt et al. 2010). In another study, three separate VSV vaccines expressing EBOV, SUDV and MARV GPs were blended together(Geisbert et al. 2009). With a single dosage of the VSV cocktail, 9 NHPs total in 3 groups of 3 NHPs survived challenges with EBOV SUDV or MARV(Geisbert et al. 2009). These two examples demonstrate the strong pre-clinical potential for achieving broad EVD protection using either a bivalent or blended vector based vaccine. This result has also been demonstrated with a protein subunit based vaccine using full length GPs of EBOV, MARV, SUDV purified from insect cells, which generated a strong protective heterologous Filoviridae response in NHPs with three doses with CoVaccine HT™ adjuvant. NHPs vaccinated with a combination of EBOV, MARV, and SUDV GP had full protection from MARV challenge, NHPs vaccinated with EBOV and SUDV survived a SUDV challenge, and NHPs vaccinated with just EBOV were protected from an EBOV challenge(Lehrer et al. 2021)."*

*In summary, the low anti-GP IgG responses observed in the non-assembling 2OBX controls are consistent with both the limits of the ELISA assay and prior literature demonstrating that multivalent I53-50 nanoparticle assembly is a key driver of enhanced antibody responses. We have now highlighted this point in the Discussion and provided additional context in the Introduction to situate our GP constructs within the broader field of filovirus vaccine research. While our immunogens lack the MPER region and further optimization may improve breadth and potency, our data support the conclusion that nanoparticle assembly substantially improves*

*GP-specific immune responses, aligning with established findings across other viral immunogens when displayed on the surface of I53-50 and other multivalent platforms.*

33. Lastly, it is concerning how bare I53-50 immune serum samples (from both mice and guinea pigs) were able to recognize EBOV GP- and/or SUDV GP-specific coating antigens up to nearly a 103 endpoint titer, suggesting that there might non-specific background signals in this ELISA. This is briefly mentioned (lines 232-234) but a explanation is not provided.

Thank you to the reviewer for highlighting this point. The EBOV and SUDV GPs used as coating antigens in the ELISA assays contained His6 tags from their purification by IMAC. Our immunogens, including the I53-50 nanoparticles, also contain His6 tags, so it is possible that the background signals observed in the ELISAs (including recognition of naked I53-50 nanoparticles) are due to the elicitation of anti-His6 antibodies. We have observed this in other studies.

We have now noted this in the limitations paragraph of the Discussion, indicating that future work will utilize tagless constructs. Specifically (lines 421-424):

"*Future research would also benefit from an exploration of the nature of T cell responses to our GP-I53-50 NP immunogens(Walls et al. 2020), the removal of His6 tags from the GP-NP immunogens, and dose range or prime-boost optimization of the mosaic or cocktail NP immunogens (versus their non-NP/assembled counterparts) for improved broad spectrum EBOV - SUDV protection.*"

34. In addition, morbidity is defined as having lost >15% of body weight (line 238). However, how was weight loss calculated in the challenge study with the mouse adapted EBOV? Based on Fig. 4d, it appears that some mice lost over 20% of their body weight.

Thank you for pointing out this discrepancy. After review, we determined that the previous definition was inaccurate. Morbidity was actually defined by clinical disease score, not body weight loss. We have corrected this in the manuscript and added a definition of clinical disease score to the Methods (lines 652-655, 635-637) and the Supplemental Figure 26 legend.

*"The full scoring/euthanasia criteria was as follows: 0 = normal (smooth coat; eyes/nose clear); 1 = reduced grooming/ruffled fur; 2 = subdued response, but normal when stimulated; 3 = lethargic; hunched posture; subdued response, even when stimulated; 4 = (euthanize) unresponsive when provoked, weak, unable to walk."*

35. Major comment #6:

The authors claim that the co-display of additional Ebolavirus GPs such as those from Marburg virus (MARV), Bundibugyo virus (BDBV), or Taï Forest virus (TAFV) could broaden protection (lines 348-350). Additionally, the authors mention that mosaic 2c-NPs may be required to elicit broad spectrum responses (lines 366-368). However, as evidenced by the survival data against both maEBOV and gpaSUDV challenges, the mosaic EBOV-GP-I53-50 + SUDV-GP-I53-50 vaccine 1) always significantly underperformed the challenge-matched GP

I53-50 alone and 2) never significantly outperformed its cocktail counterpart. Hence, there is minimal evidence to suggest that the development of a more complex mosaic NP incorporating additional filovirus GPs would broaden or improve the protection against other filoviruses, much less perform better than a cocktail counterpart. The authors have not adequately addressed this issue in the results or discussion of this manuscript.

Thank you to the reviewer for raising this point. We have revised the Introduction and Discussion to remove speculation about co-display of additional filovirus GPs, since our study was not designed to evaluate that question. However, we would like to clarify that our data does not uniformly support the conclusion that mosaic display underperforms or lacks evidence of cross-reactive benefit. In the maEBOV mouse model, the mosaic and cocktail EBOV / SUDV GP I53-50 NPs did underperform relative to the homologous EBOV-GP-I53-50 NP, and we agree that these findings do not support speculation about expanding the mosaic to additional filovirus GPs in this system and have thus removed reference to this from the text completely.

However, the gpaSUDV guinea pig model yielded a different outcome. In this model, both the mosaic EBOV / SUDV GP I53-50 NPs elicited measurable cross-reactive anti-EBOV GP antibody responses, and with five of six guinea pigs surviving the viral challenge. These results were significantly better than those observed for the mismatched EBOV-GP-I53-50 NP, and were equivalent to the cocktail formulation. Thus, the performance of the mosaic immunogen in this study was model dependent, and thus cannot be generalized from the mouse study alone.

To avoid overinterpretation, we have totally removed all forward-looking statements about adding additional filovirus GPs to the mosaic design. At the same time, we have updated the Discussion to note that our findings show divergent outcomes in mice and guinea pigs, and that mosaic nanoparticles can produce protective responses in at least one of the two BSL-4 rodent models tested.

<u>Other comments:</u>
36.1. Lines 199 – 200: The authors state that they produced “a mosaic I53-50 NP co-displaying EBOV- and SUDV-GP in a 1:1 ratio,” but provide no evidence for this claim. While the authors mixed the EBOV and SUDV subunits in a 1:1 ratio, it does not necessarily follow that the assembled NPs will maintain this ratio, since it is entirely possible that one of the trimeric GP subunits self-assembles more efficiently with the pentameric subunit.

We thank the reviewer for this important point and agree 100% that while we mixed the EBOV and SUDV subunits in a 1:1 ratio, this does not guarantee that each assembled NP will maintain this exact ratio. The surface locations and quantities of GP I53-50A immunogens on individual NPs are randomly determined through stochastic co-assembly. Statistically, one expects an approximately even distribution, with roughly 50% of each GP on average per NP, though the exact composition of any individual particle may vary. However, in previous work, we showed that the antigenic content of purified mosaic nanoparticles displaying influenza virus hemagglutinin trimers matched the ratios of antigen-bearing components in the in vitro

assembly reaction (Boyoglu-Barnum & Ellis et al., *Nature* 2021). We have added a sentence to the Results section (lines 227-230) in the revised manuscript to reference this previous finding.

37.2. Lines 367 – 368: The authors state that their results suggest that "mosaic nanoparticles displaying oligomeric antigens…may be needed to elicit broad-spectrum immune responses." In fact, their results show that a cocktail of EBOV and SUDV NPs and mosaic NPs provide nearly identical protection from challenge. Furthermore, the cocktail elicited vaccine-specific IgG levels that were either similar to or higher than those elicited by mosaic NPs.

We once again thank the reviewer for their astute comments. The following sentence has been removed from the manuscript, as we agree that it does not directly or accurately reflect the data (paragraph 413-424):

"...*mosaic nanoparticles displaying oligomeric antigens…may be needed to elicit broad-spectrum immune responses*."

38.3. In Fig. 4B-C and Fig. 5B-C, the authors compare EBOV-GP- and SUDV-GP-specific IgG titers elicited by EBOV-GP NPs, SUDV-GP NPs, EBOV/SUDV mosaic NPs, and a cocktail of EBOV and SUBV NPs and calculate P values compared to bare NPs. Since the rationale for this study is to develop a vaccine capable of protecting against both EBOV and SUDV, it would be more informative to compare the mosaic and cocktail vaccines to the relevant single-antigen vaccine. For example, in Fig. 4C the mosaic NP elicited (significantly?) lower levels of SUDV-specific IgG than SUDV NPs, while the cocktail vaccine elicited similar levels. Similarly, in Fig. 5C the cocktail vaccine elicited EBOV-specific IgG levels similar to those of the EBOV NP, while the mosaic NP elicited much lower IgG levels which were not significantly higher than those of the negative control. This is not surprising since it is well established that the efficacy of NP vaccines is largely due to the cross-linkage of B cell receptors (BCRs) by the regular and repeated array of fixed antigens. In the context of presentation to B cells, a mosaic NP has approximately half of the antigen avidity (assuming a 1:1 ratio of antigens) as a single-antigen NP.

Thank you for this note, we agree with the motivation of the reviewer, and therefore we prepared Supplemental Figure 22 which provides a statistical analysis of the Kaplan-Meier survival curves for Groups in the maEBOV study (Supplemental Figure 22a) to the survival observed for SUDV-I53-50 NP (Group 2 - Comparator) as the control in the same study. Similarly, we ran the statistical analysis of the Kaplan-Meier survival curves for Groups in the gpaSUDV study (Supplemental Figure 22b) to the survival observed for EBOV-I53-50 NP (Group 1 - Comparator) as the control in the same study. In summary, in both studies the matched GP-I53-50 NP, mosaic, or cocktail NP immunogens all demonstrate statistically significant survival benefit in comparison to the heterologous GP-I3-50 NP "control" immunogen. We believe this additional comparison is the most relevant because the immunogens in these cases are all assembled into NPs and have equivalent masses of GP components; and is based on EVD challenge rather than serology alone. These results demonstrate that mosaic and cocktail NP immunogens confer significant heterologous protection in the guinea pig model, whereas

their performance in the mouse model is more limited. Overall, we found it difficult to make direct, side-by-side comparisons between mosaic and cocktail antibody titers, so we avoided making any strong claims. In mice, mosaic produced higher anti-EBOV titers than cocktail, while cocktail produced higher anti-SUDV titers than mosaic. In guinea pigs, mosaic performed poorly compared to cocktail for anti-EBOV titers, but the two were statistically similar for anti-SUDV titers.

Minor comments:

39. 1. The SEC profile in Figure 1c. should include the unit of X-axis and Y-axis. Especially for X-axis, the manuscript has included both FPLC and HPLC. Retention time was used in some SEC profiles (Figure 2i and 2j), but retention volume might be used in Figure 1c (?). In addition, retention time should use "minute" as the unit, not "milliliter" (Supplemental Figure 2b, 3b, 3c, 3d, 7a, 7c, etc.), which should be used as the unit for retention volume.

Figure 1, 7a, 7c:: mL and mAU labels added to the X-axis and Y-Axis
Supplemental Figure 2b, 3b, 3c, 3d, 7a, 7c: This was double-checked it indeed should be mL. These SEC traces are from an FPLC runs not HPLC runs, a smaller column was used as these are small scale pilot purifications, produced through lentiviral transduction. The column has been specified in the figure legend for clarification. The HPLC column used for characterization was also added to Figure 2 legend.

40. 2. The manuscript mentioned that the HR2 stalk was not built in the cryo-EM model (line 171-172). The authors should clearly state which parts are missing in the structure.

The missing residues have now been specified in the text as residues 598-632 (Lines 192-193).

41. 3. The shoulder to the left of the trimer peak in the SEC profile of SUDV-GP-153-50A (MDT-000869-lenti) indicated that this protein was not homogenous, and this shoulder might correspond to aggregation or other higher molecular-weight GP forms. The authors may need to characterize the sample homogeneity with SEC-MALS.

The lentivirus produced samples were only used as a pilot study to test different constructs. The SUDV-GP-153-50A (MDT-000869-lenti) lentivirus produced material was investigated with negative stain EM (figure 2f-h), at the time the shoulder was not investigated further. The vaccination material was produced by transient transfection using a pCMV/R vector. We have now completed additional nsEM structural characterization on this preparation, which replaces the previous Figure 2 panels that focused on the lentiviral produced material (see updated panels 2j and 2k and lines 222-224). This preparation did not display a shoulder and reflects the exact production method and lot numbers used for the immunization studies, so no further investigation on peak shoulders was performed, as none were observed in these relevant study samples. The previous figure 2 panels representing the lentivirus produced components have now been moved to supplemental figure 7.

42. 4. The authors may want to provide more detailed description of structural detailed. The cryo-EM structure was determined at a resolution of 3.05 Å, which may not be sufficient for visualizing atomic details of the sidechains, especially when they were compared with those from a crystal structure at a resolution of 2.3 Å (Supplemental Figure 6). Sidechain orientation in the cryo-EM structure may not be as accurate as in the crystal structure.

We have added clarification in the Results section (lines 181-199) to explain that the protein backbone and many sidechains are well-defined at the reported map resolution. Readers are now directed to Figures 1h–i, which show the model built into the EM density map. The map resolution range is also now specified: 3.05 Å represents the global resolution, with local values ranging from 2.79 Å to 3.24 Å. Furthermore, we note that the atomic model was built only into regions of the map resolved to better than 3 Å, with a reference to the local resolution map shown in Supplemental Figure 4d.

43. 5. In Figure 2, the authors showed representative 2D classifications and 3D density models derived from the negative-stain EM analysis for EBOV-GP-I53-50A and SUDV-GP-I53-50A, respectively. Both I53-50A-attached ebolavirus GP trimers appeared to exhibit C3 symmetry, judged by the 2D classification images. However, the trimeric GP showed a ring-like shape instead of C3 symmetry in the 3D models. The authors should include more technical details for their negative-stain EM analysis (e.g., 2D classification and 3D modeling) and provide some explanations for the non-trimeric GP in 3D modeling.

We thank the reviewer for this helpful comment. We have expanded the methodological details of our negative stain EM workflow in the revised Results section, including additional information on particle picking, 2D classification, and 3D reconstruction procedures.

We also note that the initial 3D models shown in the previous submission were generated from GP I53-50A components produced in a lentiviral expression system. These reconstructions showed a ring-like appearance for the GP portion of the nanoparticle, which we attribute to a combination of heavy glycosylation, local flexibility within GP, and the presence of the flexible Gly-Ser linker connecting GP to the I53-50A component. As a result, those maps were refined only in C1 and did not resolve with a clear triangular geometry for the GP.

To address this point more rigorously (and as mentioned above), we repeated all negative stain EM analyses using the CMV produced GP I53-50A components that were directly used in the immunization studies. These new datasets produced substantially improved 2D classes and 3D reconstructions that clearly resolve trimeric GP density with the expected triangular density of the GP antigens when processed in C1. These updated reconstructions now replace the earlier lentiviral expression results (lines 222-224) in the main figure 2, and the original lentiviral based reconstructions have been moved to Supplemental Figure 7 for reference.

Importantly, in all lentiviral and pCMV/R produced component datasets, we observed no evidence of monomeric or unfolded GP species during our nsEM analyses. The updated figure 2 panel set now more accurately reflects the structural state of the immunogens directly used in the vaccination studies.

44. 6. The authors should provide more details about the coating GP antigens used in serum ELISA to ensure that the coating antigens do not contain foldon or other non-GP motifs that have been used in the vaccine antigens.

We have added this information to the materials and methods on the preparation of EBOV GP and SUDV GP antigens that were used in the ELISA assays. We added extensive information on production of GPs used in ELISAs (lines 669-705) See SUDV and EBOV GP Protein Production for ELISA in Materials and Methods, and Supplemental Figure 19). No foldons were used in the GP antigens used for ELISA. However, the GP antigens used for ELISA do contain a HIS tag along with some of the immunogens, this has now been mentioned in the results section and is also listed as a limitation in the discussion (lines 413-424).

45. Reviewer #3

In this paper titled "A Multivalent Pan-Ebolavirus Nanoparticle 1 Vaccine Provides Protection in Rodents from Lethal Infection by Adapted Zaire and Sudan Viruses", Brunette et al., report the design, characterization and testing of nanoparticles displaying Ebolavirus surface glycoproteins. The nanoparticles are prepared using the icosahedral I53-32 platform, and characterized using several techniques, including electron microscopy. The protective efficacy of these nanoparticles was demonstrated in mice and guinea pigs. This study addresses an important vaccine need against a viral pathogen.

This review focuses primarily on the EM studies. Overall, the studies are well done, and characterizations are extensive at each stage of the production.

We thank the reviewer for their kind words in regards to the EM work performed in this manuscript.

Comments:

46. A major deficiency in the paper is lack of characterization of the epitopes of the antibodies elicited by vaccination. While the authors note this deficiency in the discussion, given the extensive EM data shown in the paper, which attests to the fact that the authors have access to the technology, this important characterization data for the vaccine outcome should be included in the paper.

We thank the reviewer for this comment and agree that such an analysis would be of great interest. However, the cloning and characterization of vaccine-elicited antibodies are beyond the scope of this study, as limited sera availability in the original experiments precluded this type of analysis. More detailed characterization aimed at identifying SUDV/EBOV cross-neutralizing antibodies is planned for future studies and is noted as a current limitation in the Discussion.

Other comments:

47. In Figure 1: the legend should be revised to include details that aid in understanding the figure panels. For example, for panel g, no description is provided in the legend for how the subunits are visualized and colored.

Figure 1g: Color key text added to figure legend

48. Figure 1d: Bands in the SDS-PAGE should be labeled indicating which band represents each component. Same comment for 2k. The gels in the Supplement are better labeled.

Figure 1d. Bands on gel labeled. Description added to key.
Figure 2k: Bands on gel labeled. Description added to key.

49. Figure 1e: the scale bar in the figure is very hard (or impossible) to decipher.

Figure 1e: Scale bar has been updated.

50. Figure 1h: Add labels to the figure, for example, for the glycan.

Figure 1h: Glycan has been labeled.

51. The fit for the glycan in 1h (left) does not seem optimal and can be improved.

Figure 1H and F have been updated with the published map. The old panel was mistakenly out-of-date. Thank you for bringing this to our attention.

52. Figure 2b and 2f: scale bars are drawn with no indication for what the scales are.

Figure 2b 2f: Scale bars have been added.

53. Minor: Supplemental Figure 13 legend: "..the GP is flexibly attached…"

Supplemental Figure 13: The legend was updated to elaborate: GP is attached to I53-50A with a flexible GS linker.

**WEIDLE & BRUNETTE et al - RESPONSE TO REVIEWERS #1-3**
*A Multivalent Pan-Ebolavirus Nanoparticle Vaccine Provides Protection in Rodents from Lethal Infection by Adapted Zaire and Sudan Viruses*

We thank the reviewers for their continued evaluation of our manuscript and for their feedback. We appreciate the support of Reviewers #2 and #3 and thank Reviewer #1 for their additional comments. We have addressed the remaining points below and revised the manuscript accordingly.

**Reviewer #1**

The revised version of this interesting manuscript now addresses the majority of prior reviewer questions, thank you for working though the long list of topics raised. Unfortunately with some of the more substantial revisions came some inaccuracies in description of relevant prior published work. Some further topics will have to be clarified further specifically related to antigen doses.

The remaining items that should be addressed are the following:

1) it is great that more information is now included to the status of EBOV, SUDV and multivalent filovirus vaccines from various platforms including adjuvanted recombinant subunits. On the latter, in line 124 you inaccurately describe that a EBOV+MARV+SUDV GP vaccine had been tested and shown efficacy against MARV which is not described in this paper (but other combinations were tested successfully). However, another (later) publication describes a combination of SUDV+MARV GP and protective efficacy against both viruses.

We reviewed citation #32 as well as the referenced follow up publication, and have now updated lines 123-126 (123-125 in the previous revision) of the Introduction to reflect the two studies as follows:

> *"**NHPs vaccinated with a combination of EBOV and MARV GPs had full protection from MARV challenge,** NHPs vaccinated with EBOV and SUDV survived a SUDV challenge, and NHPs vaccinated with just EBOV were protected from an EBOV challenge[32]. **A follow up NHP prime-boost study using co-lyophilized SUDV plus MARV GPs afforded protection against both SUDV and MARV challenges.[116]"***

2) Thank you also for including the rationale of why mouse immunizations used the subcutaneous route. But the explanation why guinea pigs were immunized via the intra-peritoneal route is still missing. As this is an unusual route not used clinically for vaccines, a rationale should be given why IM or SC routes were not used. Also, in presenting results (for example in the summarizing lines 344-346) the findings should be relativized as the IP route may have a significantly stronger immune activation resulting in the qualitatively better protection observed over the mouse model data. The same consideration should be used when discussing data, for example in lines 364-366 where you speak about a more robust cross-reactivity which could also be linked (at least partially) to the different route of administration.

Thank you for raising this point. In coordination with USAMRIID, we have confirmed that both the mouse and guinea pig studies used subcutaneous injections. The Methods section has been corrected accordingly (lines 639–644; 631–633 in the previous revision). The main text (lines 314–316) was already accurate and has not been changed. We apologize for the confusion.

> **METHODS ONLY**
> *"Groups of 6 female Hartley guinea pigs (Charles River) per dosing group (except for Group 1 which had 5 animals due to one loss in preparation), weighing 350-500 grams were vaccinated **by the subcutaneous (s.c.) route** with a prime immunization, and three weeks later guinea pigs were boosted with a second vaccination by the **s.c.** route. **The subcutaneous route was selected***

***following guidance from IACUC to avoid potential pain at the injection site that could ensue from an intramuscular route."***

3) While the discussion now presents a more complete picture of the relevant candidates in the space of other VLP, protein and nanoparticle vaccines, it should be noted that the drosophila-expressed GP-subunits (in the context of thermostabilization) have also been extensively characterized in their self-assembled trimerization state and solution stability. Mention and discussion of this fact would be relevant in the context of your added thermostability experiment as well as in the discussion in lines 368-370.

We thank the reviewer for highlighting this work. We have reviewed and cited the additional relevant literature in the revised Results and Discussion (lines 250–253 [349–351 in the previous revision] and 378–383 [376–378 in the previous revision]). We also clarified the opening of the relevant Discussion paragraph to emphasize that it focuses on multivalent GP-based vaccines by explicitly adding the term "multivalent."

**RESULTS**

*"These results demonstrate that all EBOV and SUDV constructs retained structural integrity at temperatures well above physiological levels* ***– results consistent with previous studies of GP and nanoparticle displayed GP immunogens[41,43] –*** *indicating that they ~~are~~ likely ~~to~~ remain structurally stable during* ***immunization*** *~~by subsequent injection and immunization studies~~."*

**DISCUSSION**

*"In our hands, the production EBOV-GP-I53-50A and SUDV-GP-I53-50A components was about four-fold lower (~7.3 and 7.6 mg/L by transient transfection, respectively (Supplemental Figure 9) than that observed for SARS-CoV-2 RBD-I53-50A fusion proteins[35].* ***In addition, the GP-containing components of our EBOV-GP-I53-50 and SUDV-GP-I53-50 immunogens are thermostable up to an initial melting transition of 55°C. Further thermostability could potentially be achieved through stabilizing mutations in GP[42,117], or via drosophila-based expression systems and lyophilization[116,118]."***

4) The revised paragraph summarizing the NP-platform capabilities has been noted, however, the final sentence (especially "promising manufacturable candidates for clinical development...") in lines 393-394 seems to be a bit too optimistic given that a lot more steps of preclinical development are needed before this can be advanced into the clinic.

We have now updated lines 383-385 of our revised manuscript (378-380 in the previous revision) to further de-emphasize manufacturability.

**DISCUSSION**

*"Collectively, these results position the EBOV- and SUDV-GP-I53-50 nanoparticle immunogens as potentially promising ~~manufacturable~~ candidates for* ***the continued*** *~~clinical~~ development of broadly protective ebolavirus vaccines."*

5) In the final paragraph (lines 425-426) there seem to be some overstatements with regards to the ease of achieving a multivalent vaccine (that presumably protects humans). In particular it should be noted that immunogenicity and efficacy will have to be tested using a clinically acceptable injection route in a relevant NHP model first.

We thank the reviewer for this comment. We have de-emphasized the direct translatability of these nanoparticles and revised the concluding sentence of the Discussion to note the value of future nonhuman primate studies.

Lines 426-432 (421-426 in the previous revision)

*"Our results show that rationally designed EBOV and SUDV GP I53-50 nanoparticle immunogens form well-behaved, properly folded prefusion trimers that assemble efficiently and elicit strong protective immunity in homologous challenge models. The mosaic and cocktail formulations induced cross-reactive antibody responses in both mice and guinea pigs and provided clear protection against gpSUDV challenge in guinea pigs, supporting continued development of multivalent GP I53-50 nanoparticles for broader filovirus coverage.* ***Additional refinement of antigen composition, adjuvant formulation, immunization strategy, and evaluation in nonhuman primate models will further inform and strengthen the development of EBOV and SUDV GP I53-50 vaccines."***

In materials and methods the following was noted:

6) for both the mouse and guinea pig vaccinations you make statements that immunogens were mixed 1:1 with Addavax to reach a concentration of 0.01mg/ml. If 0.1ml was given to mice that would have resulted in 1ug doses, the results section, however, states that 3.4ug total antigen were given.

For guinea pigs the calculation would indicated that 250ul injection volume given contained 2.5ug antigen. The test states that 4.3ug total were administered per immunization. Please make this consistent and clarify.

We thank the reviewer for catching this. We had updated this information in the main text during resubmission but overlooked the corresponding Methods section. This has been corrected in the methods (lines 623-627 and 645-647; corresponding to lines 612-628 and 630-645 in the previous revision) .

**Mouse Immunizations and maEBOV Viral Challenge**

***"Final doses were as follows: Groups 1–4 received 1.9 µg of GP antigen (3.4 µg total protein) in a 100 µL injection volume, corresponding to a final GP antigen concentration of 0.019 mg/mL. Groups 5–7 received 1.6 µg of GP antigen (2.5 µg of GP-I53-50A trimer and 0.8 µg of "2OBX") in a 100 µL injection volume, corresponding to a final GP antigen concentration of 0.016 mg/mL. Group 8 received 2.5 µg of I53-50 nanoparticles in a 100 µL injection volume, with no GP antigen (0 mg/mL)."***

**Guinea Pig Immunizations and gpSUDV Viral Challenge**

***"Final doses were as follows: Groups 1–4 received 2.4 µg of GP antigen (4.3 µg total protein) in a 250 µL injection volume, corresponding to a final GP antigen concentration of 0.0096 mg/mL. Group 5 received 2.5 µg of I53-50 nanoparticles in a 250 µL injection volume, with no GP antigen (0 mg/mL)."***

7) for the ELISA section, information about the volume per well used to coat plates is missing and the statement about the dilutions tested needs to be clarified (stating starting dilution and how many subsequent dilutions rather than the current sentence containing "gradient" twice).

We've improved the accuracy of this section to include volumes. Additionally, we've also added the pre-dilution ratio used.

In Lines 660-670 (647-657 in the previous revision)

**Enzyme-Linked Immunosorbent Assay (ELISA)**

*"Endpoint IgG serum ELISAs to detect EBOV or SUDV antibodies were conducted using the following assay. ELISA plates (Falcon) were coated with recombinant SUDV-GP or recombinant EBOV-GP (NCI, Frederick MD)* ***with 50 µL*** *at 0.1 µg/well in phosphate buffer saline (PBS) (Sigma-Aldrich) and stored at 4°C overnight. After blocking with PBS/5% Milk (BioWorld) /0.05% Tween 20 (Sigma) (blocking buffer) at room temperature (RT) for 2 hours,* ***serum was diluted at 1:100 and then serially***

*__in ½ log dilutions__ with positive and negative control samples, all diluted in blocking buffer, were added to wells in duplicate, __50 µL per well__. Following 1 hour incubation at RT, plates were washed with PBS/0.05% Tween 20. The secondary antibody (goat anti-mouse or guinea pig IgG-HRP) (KPL) was diluted in the blocking buffer and was added to the plates at RT for 1 hour. After washing plates with PBS/0.05% Tween 20, 2,2'-azino-bis(3-ethylbenzothiazoline-6-sulfonic acid (ABTS) (SeraCare) was added for 30 minutes at RT and absorbance was measured at 405 nm using a SpectraMax® M5 (Molecular Probes). The reciprocal endpoint dilutions were reported."*

**Reviewer #2**

1) I have no more questions about the revised article

We thank the reviewer for their endorsement of the manuscript.

**Reviewer #3**

1) The authors have adequately addressed my critiques (related to cryo-EM).

We thank the reviewer for their endorsement of the manuscript.
